# Allele Frequency Spectra as a General Tool for Modeling Genetic Diversity

**DOI:** 10.1007/s10441-026-09524-9

**Published:** 2026-05-20

**Authors:** Ola Hössjer, Linda Laikre, Nils Ryman

**Affiliations:** 1 Department of Mathematics, Stockholm University, SE 106 91 Stockholm, Sweden; 2 Department of Zoology, Stockholm University, SE 106 91 Stockholm, Sweden

**Keywords:** Allele frequency spectrum, Bottleneck, Census size, Effective size, Exact matrix-analytic methods, Number of alleles, Grid-based numerical approximations, Gene diversity

## Abstract

In this paper we study genetic variation at a highly polymorphic locus of a monoecious or dioecious population whose census and effective sizes differ and possibly vary independently over time. More specifically, we develop a general framework for the allele frequency spectrum (AFS) at this locus, and functions of the AFS. Examples of such functions are number of alleles, number of common and rare alleles, allelic diversity, gene diversity, higher order gene diversity and Hill numbers. We develop exact recursions for the expected AFS, and its functionals, with particular interest in populations that experience a rapid (a few generations) bottleneck followed by approaching a new equilibrium between mutation and drift. A grid-based numerical algorithm is developed, which is exact for small populations and approximate for large populations. This algorithm is exemplified with exact calculations for small populations that undergo a bottleneck, and approximate calculations for a moderately large population that rapidly decreases in size.

## Introduction

### Measures of Genetic Diversity

An important characteristic of a population is its amount of genetic variation (Frankham et al. 2010; Traill et al. 2010; Hoban et al. 2021; Allendorf et al. 2022; Andersson et al. 2022). For instance, various measures of genetic variation are used in biodiversity conservation to assess and monitor population viability and adaptive potential when the environment changes.

Genetic variation will here be applied to single loci. Whereas a locus with lots of genetic variability typically has many rare alleles with a low frequency, a locus with little genetic variability rather tends to have a few common alleles. The perhaps most general locus-wise notion of genetic variation is the allele frequency spectrum (AFS); the distribution of frequencies of various alleles at a polymorphic locus (Kimura and Crow 1964; Ewens 1964; Nei and Li 1976; Watterson 1984), such as haplotype variation of a portion of DNA (even a whole gene). For such loci, with many alleles, the infinite alleles model (Kimura 1971) serves as a good approximation to describe the dynamics of the allelic composition. This locus-wise notion of AFS should not be confused with the genomewide allele frequency spectrum (Evans et al. 2007; Kaj and Mugal 2016; Jouganous et al. 2017). The genomewide version of the AFS monitors the distribution of the more recent alleles (derived from mutation) at a number of biallelic markers across the genome, based on the infinite sites model (Kimura 1969). Whereas the shape of the genomewide AFS is typically used for inferring demographics and population history (Excoffier et al. 2013), the locus-specific AFS is more appropriate for quantifying genetic variation in conservation biology settings. For instance, Allendorf et al. (2024) give a number of examples of highly polymorphic genes that are important for a species’ long term survival.

### The Present Article

#### Novelties

In this paper we restrict ourselves to the locus-wise version of the AFS. More specifically, for this type of AFS we study the dynamics of its expected form over future generations, the so called expected allele frequency spectrum (EAFS). The novelties of our approach can be summarized as follows: Firstly, previous work on the locus-specific AFS relied on asymptotically large populations over long periods of time. This led to continuous time approximations of the dynamics of the AFS, using either forward-in-time methods that make use of diffusion approximations (Kimura 1964; Nei and Li 1976), or backward-in-time methods based on coalescence theory (Watterson 1984). In contrast, we will use exact, matrix analytic methods to study the dynamics of the EAFS over time. This will allow exploring the dynamics over contemporary timescales which is of relevance for conservation of biodiversity. Secondly, our model incorporates monoecious and dioecious populations whose census and effective sizes are possibly different and vary independently over time, including population trajectories with severe bottlenecks or highly skewed sex-ratios. Thirdly, it is well known that a number of quantifiers of genetic variation (such as gene diversity and number of alleles) are functions of the locus-wise AFS. We demonstrate that not only these measures of genetic variation, but also several others, such as the number of common alleles, the number of rare alleles, allelic diversity (Allendorf 1986), gene diversity for triplets and quadruples of gene copies, and Hill numbers (Hill 1973) of various orders, are different summary statistics of the locus-specific AFS. In order to demonstrate the many applications of the AFS, we build a substantial part of our presentation around these summary statistics. In particular, we emphasize that the AFS concept makes it possible to study the time dynamics of the expected values of a large class of summary statistics of the AFS, in a unified way. Fourthly, since our exact time recursion is computationally infeasible for large populations, we develop a numerical method for computing the EAFS, which is exact for small populations and approximate for large populations. For large populations, this numerical approach does not rely on diffusion or coalescence processes, but on grid-based approximations of the EAFS.

We believe our approach is helpful for studying the effect of bottlenecks on various measures of genetic variation. In particular it leads to exact analyses of intense bottlenecks, where the population is reduced to a very small size. It is well known that a short and intense bottleneck reduces the number of alleles a lot more than it reduces gene diversity (Allendorf 1986; Fuerst and Maruyama 1986; Ryman et al. 1995; England et al. 2003). This is important for genetic monitoring and conservation management, since gene diversity quantifies a population’s short term evolutionary potential, whereas the number of alleles is more important for long time disease resistance and survival (Allendorf et al. 2024).

#### Why is the Locus-wise AFS Needed?

What is the main motivation for analyzing the locus-wise AFS, and its time dynamics, in the first place? The answer to this question is twofold. Firstly, the AFS provides a general framework for studying a number of different quantifiers of genetic variation, as described in Section 1.2.1. Secondly, in order to study the time dynamics of several measures of genetic variation (such as the number of alleles), the whole AFS is needed, not only the diversity measure itself. Since the AFS involves not only the effective size of the population, but also the census size, this explains why both the effective size and census size are needed in order to fully understand how genetic variation evolves over time. It is true that simple and well understood recursions, that only involve the effective size, are available for gene diversity (see for instance pages 101–104 of Crow and Kimura (1970)). But this is rather an exception for this particular measure of genetic variation.

#### Organization

Our paper is organized as follows: The population genetic model is presented in Section 2, with separate treatments of monoecious and dioecious populations. The locus-specific AFS, and a number of its summary statistics, are introduced in Section 3. After deriving the dynamics of the frequency fluctuations of a single allele in Section 4, we use these results to study time recursions for the expected values of the AFS, and its summary statistics, in Sections 5 and 6. Populations that either have constant size or experience a sudden population size change, are treated in Sections 7 and 8, respectively. In particular, we investigate how the expected AFS, and its summary statistics, approach new values when an equilibrium between genetic drift and mutation is attained. The approximate algorithm for computing the EAFS is introduced in Section 9. Numerical illustrations are given in Section 10, using the exact EAFS algorithm for small populations that undergo a bottleneck, and the approximate algorithm for moderately large populations that experience a permanent population size decline. Then a discussion is offered in Section 11. Finally, a number of mathematical results are presented in Appendices A–C. A summary of the most important notation is provided in Table 1.

**Table 1:** Summary of the most important notation

Quantity	Description
*t*	Generation number ($\in \{0,1,2,\ldots \}$)
*N*(*t*)	Census size of generation *t*
$N_e(t)$	Effective population size of generation *t*
*d*(*t*)	Ratio of effective and census sizes in generation *t* ($=N_e(t)/N(t)$)
$N_{m}(t)$	Number of males of generation *t* (for a dioecious population)
$N_{f}(t)$	Number of females of generation *t* (for a dioecious population)
$N_{em}(t)$	Number of male breeders of generation *t* (for a dioecious population)
$N_{ef}(t)$	Number of female breeders of generation *t* (for a dioecious population)
*μ*	Mutation probability per gene copy, in each generation, also referred to as mutation rate
*X*(*t*)	Frequency of one allele (say *a*) at the locus of interest in generation *t*
*x*	Generic notation for the frequency of one allele at a locus that is either polymorphic (*0<x<1*) or not (*x=1*). Lost alleles correspond to *x=0*.
$f_x(t)$	Number of alleles with frequency *x* in generation *t*
${{{\mathcal {X}}}}(t)$	Set of possible allele frequencies of generation *t*
$\boldsymbol{ f}(t)$	Allele frequency spectrum (AFS) of generation *t*, a row vector equal to $(f_x(t);\, x\in {{{\mathcal {X}}}}(t))$
*g*	Function $[0,1]\rightarrow {{\mathbb {R}}}$ assigning weight *g*(*x*) to allele frequency *x*
$\boldsymbol{ g}(t)$	Column vector assigning weights $(g(x); \, x\in {{{\mathcal {X}}}}(t))^\prime$ to all allele frequencies of generation *t*
$S_g(t)$	Summary statistic ($=\sum _{x\in {{{\mathcal {X}}}}(t)} f_x(t)g(x)$) that is a linear function of the AFS of generation *t*, based on weight function *g*
$A_n(t)$	Number of (currently present) distinct alleles of generation *t* at the locus of interest
$A_{n \mathrm{tot}}(t)$	Total number of distinct alleles that either are currently present in generation *t* or were present before generation *t* at the locus of interest
$A_{n \mathrm{co}}(t)$	Number of distinct common alleles of generation *t* at the locus of interest
$A_D(t)$	Allelic diversity of generation *t*
${\bar{x}}(t)$	Average frequency of rare alleles of generation *t*
*H*(*t*)	Gene diversity of generation *t*
$\tilde{H}(t)$	Modified gene diversity of generation *t* (normalized differently than *H*(*t*))
$H_q(t)$	*q*-tuple gene diversity of generation *t* (with *q≥ 2* an integer), having gene diversity ($H_2(t)=H(t)$), triplet gene diversity ($H_3(t)$) and quadruple gene diversity ($H_4(t)$) as special cases
$\tilde{H}_q(t)$	Modified *q*-tuple gene diversity of generation *t* (normalized differently than $H_q(t)$)
$D_q(t)$	Hill number (or effective number of alleles) of order *q* in generation *t*, with *q≥ 0* real-valued
$\boldsymbol{ P}(t)$	Matrix with transition probabilities $P_{xy}(t)$ between all pairs of allele frequencies *x* and *y* of generations *t* and *t+1*, for the Markov process *X*(*t*)
$\boldsymbol{ P}$	Matrix with transition probabilities of the Markov process *X*(*t*) for a population whose census and effective sizes are constant over time
$\lambda _q$	*(q+1)*th largest eigenvalue of the matrix $\boldsymbol{ P}$
$\boldsymbol{ l}_q$	Left eigenvector corresponding to $\lambda _q$
$\boldsymbol{ r}_q$	Right eigenvector corresponding to $\lambda _q$
$F_x(t)$	Expected number of alleles with frequency *x* in generation *t*
$\boldsymbol{ F}(t)$	Expected allele frequency spectrum (EAFS) of generation *t*, a row vector $(F_x(t);\, x\in {{{\mathcal {X}}}}(t))$
*θ (t)*	Census size normalized mutation rate of generation *t* (=*N(t)μ*)
$\theta _e(t)$	Effective size normalized mutation rate of generation *t* (=$N_e(t)\mu$)
*\varepsilon (t)*	Allele frequency corresponding to one single gene copy in generation *t* (*=1/[2N(t)]*)
$\boldsymbol{ \delta }_{\varepsilon (t)}$	Row vector that assigns a value 1 to allele frequency *\varepsilon (t)* and a value zero to all other possible allele frequencies of generation *t*
$\hat{{{{\mathcal {X}}}}}(t)$	Grid-based approximation of the set of allele frequencies of generation *t*
$\hat{\boldsymbol{ F}}(t)$	Grid-based approximation of the expected allele frequency spectrum of generation *t*, a row vector $({\hat{F}}_x(t);\, x\in \hat{{{{\mathcal {X}}}}}(t))$

## Population Genetic Model

Consider a population with non-overlapping generations *t=0,1,2,…* whose census size and inbreeding effective size at time *t* is *N*(*t*) and $N_e(t)\le N(t)$, respectively. Effective size is a crucial parameter in evolutionary and conservation genetic research (Waples 2022). However, in order to study genetic variation it is important to consider not only the effective size, but also the census size (Ryman et al. 2023). We will therefore include both (various versions of) the effective size and the census size when studying genetic variation of a population in terms of allelic variation at a polymorphic locus, referred to as a gene. Each individual carries two copies of the gene, so that 2*N*(*t*) gene copies are available at time *t*. These gene copies are of different types and represent different alleles. It is assumed that the population evolves over time through the forces of genetic drift and mutation, according to the infinite alleles model (Kimura and Crow 1964; Kimura 1971).

We distinguish between a monoecious (one-sex) population and a dioecious (two-sex) population. The dynamics of allelelic variation, for these two populations, is described in Sections 2.1 and 2.2.

### Monoecious Population

The reproduction cycle of a monoecious population between generations *t* and *t+1* is defined through the following three steps:

1. Among the 2*N*(*t*) gene copies of generation *t*, $2N_e(t)$ are selected randomly without replacement as gene copies of $N_e(t)$ breeders, with $N_e(t)\le N(t)$.
2. *2N(t+1)* gene copies are drawn randomly with replacement from the pool of $2N_e(t)$ gene copies in Step 1 (where *N(t+1)* could be equal to or smaller/larger than $N_e(t)$).
3. Each of the *2N(t+1)* gene copies of Step 2 independently, with probability $0\le\mu\le 1,$ mutates to a new allele which has never been present in the population before.

By randomly pairing the 2*N*(*t*) gene copies of each generation *t* into *N*(*t*) diploid individuals, we obtain a monoecious, diploid model with a probability of self-fertilization $1/(2N_e(t))+[1-1/(2N_e(t))]/(2N(t)-1)$ when individuals of generation *t+1* are formed. This monoecious, diploid model is an idealization, where only two time points of the life cycle between two successive generations are included; selection of $N_e(t)$ breeders (Step 1) and formation of *N(t+1)* candidate breeders for the next generation (Steps 2–3). The timing of Steps 2–3 is not obvious; *N(t+1)* could for instance be chosen as the number of zygotes or the number of juveniles just prior to breeding, for the next generation *t+1*. Depending on species and ecological context, the number of juveniles could be much smaller than the number of zygotes.

Note that the population reduces to a Wright-Fisher population (Fisher 1922; Wright 1931) with mutations according to an infinite alleles model when $N_e(t)=N(t)$ (so that essentially, Step 1 can be removed). If additionally the population starts with one single allele at *t=0*, and all subsequent mutations are clumped into a second allele, we obtain a biallelic Wright-Fisher model with one-way mutations (Ewens 2004, p. 95).

The number of breeders in Step 1 corresponds to a haploid inbreeding effective size

1 $$\begin{aligned} N_e(t) = N_{eI}(t) = \frac{1}{2\pi _2(t)}, \end{aligned}$$

where $\pi _2(t)$ is the probability that two distinct gene copies of generation *t+1* originate from the same gene copy of generation *t* (Ewens 2004; Durrett 2008; Hössjer et al. 2014). This haploid inbreeding effective size in (1) is also a diploid effective size, with $\pi _2(t)$ interpreted as the probability that the two distinct gene copies from the same individual of generation *t+1*, originate from the same gene copy of generation *t*. However, for populations that consist of almost isolated subpopulations, the haploid and diploid inbreeding effective sizes may differ a lot, and the latter monitors the rate at which inbreeding coefficients increase between two successive generations (Hössjer et al. 2016; Ryman et al. 2023).

### Dioecious Population

The reproduction cycle of a dioecious population between generations *t* and *t+1* is divided into the following three steps:

1. Among the $2N(t)=2N_m(t)+2N_f(t)$ gene copies of generation *t*, of which $2N_m(t)$ occur among males and $2N_f(t)$ among females, $2N_{em}(t)\le 2N_m(t)$ are selected randomly without replacement as gene copies of $N_{em}(t)$ male breeders, and $2N_{ef}(t)\le 2N_f(t)$ are selected randomly without replacement as gene copies of $N_{ef}(t)$ female breeders, with $N_{em}(t)+N_{ef}(t)\le N(t)$.
2. *N(t+1)* gene copies are drawn randomly with replacement from the male breeding pool of Step 1, and *N(t+1)* gene copies are drawn randomly with replacement from the female breeding pool of Step 1.
3. Each of the *2N(t+1)* gene copies of Step 2 independently, with probability $0\le \mu \le 1,$ mutates to a new allele which has never been present in the population before.

Suppose the 2*N*(*t*) gene copies of each generation *t* are paired into *N*(*t*) diploid individuals, with each individual inheriting one gene copy from a male and female breeder, respectively. Additionally, we may randomly choose $N_m(t)$ of these diploid individuals of generation *t* as males, and the remaining $N_f(t)=N(t)-N_m(t)$ individuals as females. It then follows that the resulting diploid, dioecious population has no self-fertlilization. As for a monoecious population, the population only includes two time points of the reproduction cycle; selection of male and female breeders (Step 1) and formation of candidate male and female breeders for the next generation (Steps 2–3). If $N_{em}(t)+N_{ef}(t)=N(t)$, this is essentially a two-sex Wright-Fisher population with mutations occuring according to the infinite alleles model. Step 1 can then be removed, since all individuals of the population are breeders.

An approximation of the effective size of the population in generation *t* is

2 $$\begin{aligned} N_e(t) = \frac{4N_{em}(t)N_{ef}(t)}{N_{em}(t)+N_{ef}(t)}, \end{aligned}$$

see for instance Section 7.6.2 of Crow and Kimura (1970). Note in particular that the haploid, dioecious inbreeding effective size

$$\begin{aligned} N_{eI}(t) = \frac{1}{2\pi _2(t)} = \frac{N(t)-1/2}{N(t)-1} \cdot N_e(t) \end{aligned}$$

essentially equals $N_e(t)$ for large populations, with $\pi _2(t)$ the probability that two randomly chosen distinct gene copies of generation *t+1* descend from the same gene copy of generation *t* (Ewens 2004, page 123). As in Section 2.1, this haploid, dioecious effective size should not be confused with a diploid, dioecious effective size, which quantifies the rate at which inbreeding coefficients increase between two successive generations (Hössjer et al. 2015).

## The Allele Frequency Spectrum and Its Summary Statistics

For a given polymorphic locus (or gene), an allele that is present in the population in generation *t* appears as *1,2,… ,2N(t)-1* or 2*N*(*t*) gene copies. An allele that was lost before generation *t* will not reenter the population at time *t* or later, because of properties of the infinite alleles model. Hence, the frequency of a (present or lost) allele of generation *t* belongs to the set

3 $$\begin{aligned} {{{\mathcal {X}}}}(t) = \{0,\frac{1}{2N(t)},\ldots ,\frac{2N(t)-1}{2N(t)},1\} \end{aligned}$$

of frequencies, where a positive (null) frequency corresponds to a present (lost) allele.

To avoid confusion with empirical population genetics, we only consider population parameters, not sample statistics. For each $x\in {{{\mathcal {X}}}}(t)$ with *x>0*, $f_x(t)$ refers to the number of alleles in generation *t*, at the locus of interest, whose frequency is *x*. For *x=0* it is assumed that $f_0(0)=0$, whereas $f_0(t)$ for *t>0* refers to the number of alleles that have been present in the population at the studied locus for at least one of generations *0,1,… ,t-1*, but is lost by generation *t*. The locus-wise allele frequency spectrum (AFS) of generation *t* is a row vector

4 $$\begin{aligned} \boldsymbol{ f}(t) = (f_x(t); \, x\in {{{\mathcal {X}}}}(t)) \end{aligned}$$

of dimension *2N(t)+1*, whose first component $f_0(t)$ is the number of alleles that were lost before generation *t*, whereas the remaining 2*N*(*t*) components of $\boldsymbol{ f}(t)$ quantify genetic variation of generation *t*. In principle, (4) can also be used to define the genomwide AFS. However, the interpretation of (4) is very different for the genomewide AFS, with $f_x(t)$ the number of biallelic markers whose derived allele has frequency *x>0* in generation *t*, whereas $f_0(t)$ is the number of alleles that have been lost before generation *t*, at any of the studied loci.

In order to summarize the AFS, we introduce summary statistics

5 $$\begin{aligned} S_g(t) = \sum _{x\in {{{\mathcal {X}}}}(t)} f_x(t)g(x) = \boldsymbol{ f}(t)\boldsymbol{ g}(t) \end{aligned}$$

that are linear functions of the AFS, for various real-valued functions $g:[0,1]\rightarrow {{\mathbb {R}}}.$ Here

6 $$\begin{aligned} \boldsymbol{ g}(t) = (g(x); \, x\in {{{\mathcal {X}}}}(t))^\prime \end{aligned}$$

is a column vector of dimension *2N(t)+1* constructed from *g*, with ${\boldsymbol{v}}^\prime$ referring to matrix transposition of a vector. Below we give several examples of summary statistics of the AFS, each of which is either a linear function (5) of the AFS, or it combines one or two such linear functions of the AFS, as summarized in Table 2.

**Table 2:** Summary statistics of the locus-wise allele frequency spectrum of generation *t* at the locus that this spectrum refers to. These summary statistics include one or two linear statistics $S_g(t)$ of type (5), with possibly different choices of the function *g* in (6). These functions are all defined in the examples of Sect. 3

Name	Notation	Definition
Number of distinct alleles of generation *t*	$A_n(t)$	$S_{g_{A_n}}(t)$
Total number of distinct alleles up to generation *t*	$A_{n\mathrm{tot}}(t)$	$S_{g_1}(t)$
Allelic diversity of generation *t*	$A_D(t)$	$(S_{g_{A_n}}(t)-1)/(S_{g_{A_n}}(0)-1)$
Total allele frequency of generation *t*	1	$S_{g_{\tiny \mathrm{id}}}(t)$
Number of distinct common alleles of generation *t*	$A_{n\mathrm{co}}(t)$	$S_{g_{\tiny \mathrm{co}}}(t)$
Number of distinct rare alleles of generation *t*	$A_{n\mathrm{ra}}(t)$	$S_{g_{\tiny \mathrm{ra}}}(t)$
Total frequency of rare alleles of generation *t*	–	$S_{g_{\tiny \mathrm{id}}g_{\tiny \mathrm{ra}}}(t)$
Average frequency of rare alleles of generation *t*	${\bar{x}}(t)$	$S_{g_{\tiny \mathrm{id}}g_{\tiny \mathrm{ra}}}(t)/S_{g_{\tiny \mathrm{ra}}}(t)$
Gene diversity of generation *t*	*H*(*t*)	$S_{g_H}(t)$
Modified gene diversity of generation *t*	$\tilde{H}(t)$	$S_{g_{\tilde{H}}}(t)$
Gene identity of generation *t*	${\bar{H}}(t)$	$S_{g_{{\bar{H}}}}(t)$
Triplet gene diversity of generation *t*	$H_3(t)$	$S_{g_{H_3}}(t)$
Modified triplet gene diversity of generation *t*	$\tilde{H}_3(t)$	$S_{g_{\tilde{H}_3}}(t)$
Quadruple gene diversity of generation *t*	$H_4(t)$	$S_{g_{H_4}}(t) + 3S^2_{g_{{\bar{H}}}}(t)$
Modified quadruple gene diversity of generation *t*	$\tilde{H}_4(t)$	$S_{g_{\tilde{H}_4}}(t) + 3S^2_{g_{{\tilde{{\bar{H}}}}}}(t)$
Hill number of order *q* of generation *t*	$D_q(t)$	$h_q(S_{g_{\tiny \mathrm{Hill}q}}(t))$
Effective number of distinct alleles of generation *t*	$D_2(t)=A_e(t)$	$1/[1-S_{g_H}(t)]$

### Example 1

*(Number of alleles.)* Let

7 $$\begin{aligned} 1\le A_n(t) \le 2N(t) \end{aligned}$$

refer to the number of distinct alleles present in the population at the locus of interest in generation *t*. The notation $A_n$ is an acronym for “**n**umber (of) **A**lleles” (cf. Table 1 of Allendorf et al. (2024)). Suppose the frequencies of these $A_n(t)$ alleles are $x_k=x_k(t)$ for $k=1,\ldots ,A_n(t)$. The locus-specific AFS of generation *t* then takes the form

8 $$\begin{aligned} \boldsymbol{ f}(t) = \sum _{k=1}^{A_n(t)} \boldsymbol{ \delta }_{x_k}, \end{aligned}$$

where $\boldsymbol{ \delta }_{x_k}$ is a row vector of dimension *2N(t)+1*, with a 1 in the position of (3) that corresponds to $x_k$, and zeros elsewhere. In particular,

$$\begin{aligned} \boldsymbol{ f}(t) = \boldsymbol{ \delta }_1 \end{aligned}$$

is the AFS at a locus where one allele has been fixed ($A_n(t)=1$), whereas

$$\begin{aligned} \boldsymbol{ f}(t) = \boldsymbol{ \delta }_x + \boldsymbol{ \delta }_{1-x} \end{aligned}$$

is the AFS of a biallelic marker with frequencies *0<x<1* and *1-x* of its two alleles ($A_n(t)=2$). Although (8) holds for the genomewide AFS as well, the interpretation is very different, with $A_n(t)$ the number of biallelic markers in generation *t*. In particular, (7) need not hold for the genomewide AFS, since the number of biallelic markers may exceed 2*N*(*t*).

Let

9 $$\begin{aligned} g_{A_n}(x) = \left\{ \begin{array}{ll} 1, & 0 < x \le 1,\\ 0, & x=0, \end{array}\right. \end{aligned}$$

be an indicator for whether an allele with frequency *x* is present in the population or not. The function $g_{A_n}$ in (9) is used for defining a summary statistic of the AFS at generation *t* that corresponds to the number of alleles

10 $$\begin{aligned} A_n(t) = \sum _{\begin{array}{c} x\in {{{\mathcal {X}}}}(t)\\ x>0 \end{array}} f_x(t) = S_{g_{A_n}}(t) \end{aligned}$$

at a specific locus at this generation. Allelic diversity

11 $$\begin{aligned} A_D(t) = \frac{A_n(t)-1}{A_n(0)-1} = \frac{S_{g_{A_n}}(t)-1}{S_{g_{A_n}}(0)-1} \end{aligned}$$

is a variable that quantifies the effect population size changes after generation 0 have on the remaining fraction of excess number $A_n-1$ of alleles at generation *t* (Allendorf 1986; Allendorf et al. 2024). As seen from (11), it can be represented in terms of two summary statistics, both based on (9), but evaluated at different time points 0 and *t*.

### Example 2

*(Total allele frequency is 1.)* Since the sum of the allele frequencies of all $A_n(t)$ alleles at a locus is 1 for any generation *t*, the summary statistic of the identity (id) function $g_{\mathrm{id}}(x)=x$ satisfies

12 $$\begin{aligned} S_{g_{\tiny \mathrm{id}}}(t) = \sum _{x\in {{{\mathcal {X}}}}(t)} x f_x(t) = \sum _{k=1}^{A_n(t)} x_k = 1. \end{aligned}$$

That is, every locus-specific AFS has a summary statistic of 1, when $g_{\mathrm{id}}$ is used to define the statistic. Note also that (12) does not hold for the genomewide AFS.    

### Example 3

*(Number of common and rare alleles.)* Let $0<\eta \le 1$ be a fixed number. When *η <1* we refer to an allele as rare or common depending on whether its frequency is at most or larger than *η*, respectively. Introduce the two functions

$$\begin{aligned} \begin{array}{rcl} g_{\mathrm{co}}(x) & =& 1(x> \eta ),\\ g_{\mathrm{ra}}(x) & =& 1(0 < x \le \eta ) \end{array} \end{aligned}$$

as indicators for a common (co) and a rare (ra) allele, respectively. The summary statistic

13 $$\begin{aligned} A_{n\mathrm{co}}(t) = \sum _{k=1}^{A_n(t)} 1(x_k> \eta ) = \sum _{\begin{array}{c} x\in {{{\mathcal {X}}}}(t)\\ x> \eta \end{array}} f_x(t) = S_{g_{\tiny \mathrm{co}}}(t) \end{aligned}$$

of the locus-specific AFS refers to the number of distinct common alleles, at the studied locus, in the population at time *t*. It follows from (10) and (12) that

14 $$\begin{aligned} 0 \le A_{n\mathrm{co}}(t) \le \min (A_n(t),[\eta ^{-1}]), \end{aligned}$$

where $[\eta ^{-1}]$ is the largest integer smaller than or equal to $\eta ^{-1}$. Note that $A_{n\mathrm{co}}(t)=0$ at a highly polymorphic locus, at which all alleles are rare. For the genomewide AFS, $A_{n\mathrm{co}}(t)\le A_n(t)$ rather refers to the number of biallelic markers whose derived allele is common. This number of biallelic markers need not satisfy (14).    

Analogously, $S_{g_{\tiny \mathrm{ra}}}(t)$ refers to the number of distinct rare alleles at time *t*, at the locus of interest. The average frequency of rare alleles at time *t* is defined (whenever at least one such allele exists) as a ratio

15 $$\begin{aligned} {\bar{x}}(t) = \frac{\sum _{k=1}^{A_n(t)} x_k 1(x_k\le \eta )}{\sum _{k=1}^{A_n(t)} 1(x_k\le \eta )} = \frac{\sum _{x\in {{{\mathcal {X}}}}(t)} x 1(0< x\le \eta ) f_x(t)}{\sum _{x\in {{{\mathcal {X}}}}(t)} 1(0 < x\le \eta ) f_x(t)} = \frac{S_{g_{\tiny \mathrm{id}}g_{\tiny \mathrm{ra}}}(t)}{S_{g_{\tiny \mathrm{ra}}}(t)} \end{aligned}$$

between two summary statistics with functions $g_{\mathrm{id}}(x)g_{\mathrm{ra}}(x) = x 1(0<x\le \eta )$ and $g_{\mathrm{ra}}(x)$, respectively. In particular, if *η =1*, then ${\bar{x}}(t)=1/A_n(t)$ is the average frequency of *all* alleles. In Appendix B.2 we derive an approximate formula for the locus-specific ${\bar{x}}(t)$, for large populations of constant size.

### Example 4

*(Total number of historical and currently present alleles.)* Let $A_{n\mathrm{tot}}(t)$ refer to the total number of distinct alleles that have been part of the population, at the locus of interest, in a least one of generations *0,… ,t*. This includes all $A_n(t)$ alleles that are present in the population in generation *t*, as well as those $f_0(t)$ historic alleles that have been lost at some earlier time point. Consequently,

$$\begin{aligned} A_{n\mathrm{tot}}(t) = f_0(t) + A_n(t) = \sum _{x\in {{{\mathcal {X}}}}(t)} f_x(t) = S_{g_1}(t) \end{aligned}$$

is a summary statistic of the locus-wise AFS, based on $g_1(x) = 1$. For the genomewide AFS, $A_{n\mathrm{tot}}(t)$ rather refers to the number of genomic positions that either harbor a biallelic marker in generation *t*, or have harbored a biallelic marker before generation *t*.

### Example 5

*(Gene diversity)* Let *H*(*t*) refer to the gene diversity of generation *t*, i.e. the probability that two gene copies, drawn randomly *with* replacement, have different alleles (Nei 1973, 1975). Gene diversity is a summary statistic

16 $$\begin{aligned} H(t) = \sum _{k=1}^{A_n(t)} \sum _{l;l\ne k} x_k x_l = \sum _{k=1}^{A_n(t)} x_k(1-x_k) = \sum _{x\in {{{\mathcal {X}}}}(t)} f_x(t) x(1-x) = S_{g_{H}}(t) \end{aligned}$$

of the locus-specific AFS, based on

17 $$\begin{aligned} g_{H}(x) = x(1-x). \end{aligned}$$

Gene diversity is the most frequently used measure of genetic variability, and it is generally assumed that it relates to a population’s short term evolutionary potential (Franklin 1980). For a population in Hardy-Weinberg equilibrium, gene diversity is equivalent to heterozygosity, the fraction of heterozygous genotypes in the population, at the studied locus. For the genomewide AFS, (16) is rather half the *total* gene diversity (or half the total heterozygosity under HW equilibrium) of the $A_n(t)$ biallelic markers that are present in the population in generation *t*.

The gene identity is the probability that two gene copies, drawn randomly *with* replacement, have the same allele at the locus of interest. It is also a summary statistic

$$\begin{aligned} {\bar{H}}(t) = 1 - H(t) = \sum _{k=1}^{A_n(t)} x_k^2 = \sum _{x\in {{{\mathcal {X}}}}(t)} f_x(t) x^2 = S_{g_{{\bar{H}}}}(t) \end{aligned}$$

of the locus-specific AFS, based on $g_{{\bar{H}}}(x) = x^2$. A modified version of gene diversity is $\tilde{H}(t)$, the probability that two gene copies, drawn randomly *without* replacement, have different alleles at time *t*. Among other things, it is a useful statistic for defining the inbreeding effective size (Malécot 1951; Hössjer et al. 2014). It corresponds to a summary statistic

18 $$\begin{aligned} \tilde{H}(t) = \frac{H(t)}{1-\frac{1}{2N(t)}} = S_{g_{\tilde{H}}}(t) \end{aligned}$$

of the AFS, based on $g_{\tilde{H}}(x;t) = x(1-x)/[1-1/(2N(t))]$.

### Example 6

*(Gene diversity for triplets of gene copies.)* Denote by $H_3(t)$ the probability that three gene copies, drawn randomly *with* replacement from the population in generation *t*, have three different alleles. For triploid organisms, with three copies of each chromsome, it gives the probability these three chromosome copies have different alleles at a fixed locus. In analogy with Example 5 we refer to $H_3(t)$ as the gene diversity for triplets of gene copies, abbreviated as triplet gene diversity. Note that

19 $$\begin{aligned} \begin{array}{rcl} H_3(t) & =& \sum _{k=1}^{A_n(t)} \sum _{l; l\ne k} \sum _{m;m\notin \{l,k\}} x_k x_l x_m \\ & =& \sum _{k=1}^{A_n(t)} x_k [(1-x_k)^2 - \sum _{l; l\ne k} x_l^2]\\ & =& \sum _{k=1}^{A_n(t)} x_k [(1-x_k)^2 + x_k^2 - \sum _{l=1}^{A_n(t)} x_l^2]\\ & =& \sum _{k=1}^{A_n(t)} [x_k(1-x_k)^2 - x_k^2(1-x_k)]\\ & =& S_{g_{H_3}}(t) \end{array} \end{aligned}$$

is a summary statistic for the AFS, based on

20 $$\begin{aligned} \begin{array}{rcl} g_{H_3}(x) & =& x(1-x)^2 - x^2(1-x)\\ & =& x(1-x)(1-2x), \end{array} \end{aligned}$$

where in the fourth step of (19) we made use of (12). Since a nonzero value of $H_3(t)$ requires at least three alleles ($A_n(t)\ge 3$), it is more sensitive to large values of the number $A_n(t)$ of alleles than gene diversity *H*(*t*). We may therefore interpret $H_3(t)$ as a summary statistic of the AFS that is intermediate between *H*(*t*) and $A_n(t)$. A modified version of triplet gene diversity is

21 $$\begin{aligned} \tilde{H}_3(t) = \frac{H_3(t)}{(1-\frac{1}{2N(t)})(1-\frac{2}{2N(t)})} = S_{g_{\tilde{H}_3}}(t) \end{aligned}$$

which is only well defined when *N(t)>1*. It is the probability that three gene copies, drawn *without* replacement, are all different. It is also a summary statistic of the AFS, based on

22 $$\begin{aligned} g_{\tilde{H}_3}(x;t) = \frac{g_{H_3}(x) }{(1-\frac{1}{2N(t)})(1-\frac{2}{2N(t)})}. \end{aligned}$$

The triplet gene identity (${\bar{H}}_3(t)$) is the probability that three gene copies, drawn at random *with* replacement in generation *t*, are identical. It corresponds to a symmary statistic of the AFS with $g_{{\bar{H}}_3}(x)=x^3$.

### Example 7

*(q-tuple gene diversity.)* The summary statistics of the previous two examples can be generalized to *q*-tuples of gene copies, where $q\in \{1,2,\ldots ,2N(t)\}$. The *q*-tuple gene identity summary statistic ${\bar{H}}_q(t)=S_{g_{{\bar{H}}_q}}(t)$ of generation *t* corresponds to a function $g_{H_q}(x) = x^q$. It is the probability that *q* gene copies, drawn *with* replacement in generation *t*, all have the same allele. The corresponding probability $H_q(t)$ that the *q* gene copies have different alleles involves one or several summary statistics of the AFS at time *t*. For instance,

23 $$\begin{aligned} \begin{array}{rcl} H_4(t) & =& \sum _{k,l.m,o} x_k x_l x_m x_o\\ & =& 1 - \sum _k x_k^4 - 4\sum _{k,l} x_k^3 x_l - 3\sum _{k,l} x_k^2 x_l^2 - 6\sum _{k,l,m} x_k^2 x_l x_m\\ & =& \sum _k x_k - \sum _k x_k^4 - 4 \sum _{k} x_k^3 (1-x_k) - 3\sum _{k,l} x_k^2 x_l^2\\ & -& 6\sum _{k} x_k^2 [(1-x_k)^2 - \sum _{l} x_l^2]\\ & =& \sum _k [x_k - 6x_k^2 + 8x_k^3 - 6x_k^4] + 3(\sum _k x_k^2)^2\\ & =& S_{g_{H_4}}(t) + 3 S^2_{g_{{\bar{H}}}}(t), \end{array} \end{aligned}$$

where $1\le k,l,m,o \le A_n(t)$ are mutually different integers in all summations. Note that $H_4(t)$ is of relevance for tetraploid organisms, with four copies of each chromsomse, since it gives the probability that these four copies are different in generation *t*, at a particular locus. As seen from (23), $H_4(t)$ involves two summary statistics based on $g_{{\bar{H}}}(x)=x^2$ and

24 $$\begin{aligned} \begin{array}{rcl} g_{H_4}(x) & =& x - 6x^2 + 8x^3 - 6x^4\\ & =& x(1-x)(1-2x)(1-3x) - 3x^3. \end{array} \end{aligned}$$

The larger *q* is, the more conservative $H_q(t)$ is as a measure of genetic variability, in the sense that

$$\begin{aligned} A_n(t) \ge q \Longleftrightarrow H_q(t)> 0, \end{aligned}$$

so that at least *q* different alleles are required for $H_q(t)$ to be positive. When *q* is large, we may therefore interpret $H_q(t)$ as a measure of genetic variation that monitors whether a locus has many (at least *q*) alleles or not. Consider, for instance, the value of $H_4(t)$ at a locus with $A=A_n(t)$ equally frequent alleles, so that

25 $$\begin{aligned} \boldsymbol{ f}(t) = A\boldsymbol{ \delta }_{1/A}. \end{aligned}$$

It follows from (23)-(24) that

$$\begin{aligned} \begin{array}{rcl} H_4(t) & =& A \cdot \frac{1}{A} \left( 1-\frac{1}{A}\right) \left( 1-\frac{2}{A}\right) \left( 1-\frac{3}{A} \right) - 3A\left( \frac{1}{A}\right) ^3 + 3 \left[ A (\frac{1}{A})^2\right] ^2\\ & =& \left( 1-\frac{1}{A}\right) \left( 1-\frac{2}{A}\right) \left( 1-\frac{3}{A} \right) \end{array} \end{aligned}$$

at this particular locus, proving that $H_4(t)>0$ if and only if *A≥ 4*.

A slightly modified version of the *q*-tuplet gene diversity, $\tilde{H}_q(t)$, is the probability that *q* gene copies, drawn randomly *without* replacement from the population at time *t*, have different alleles. In analogy with (18) and (21), it is defined as

26 $$\begin{aligned} \tilde{H}_q(t) = \frac{H_q(t)}{\prod _{r=1}^{q-1}(1-\frac{r}{2N(t)})} =: \frac{H_q(t)}{C_q(t)} \end{aligned}$$

whenever *N(t)≥ q/2*. For instance, it follows from (23) and (26) that the modified quadruple gene diversity is $\tilde{H}_4(t)=S_{g_{\tilde{H}_4}}(t) + 3S^2_{g_{{\tilde{{\bar{H}}}}}}(t)$, with $g_{\tilde{H}_4}(x;t) = g_{H_4}(x)/C_4(t)$ and $g_{{\tilde{{\bar{H}}}}}(x;t) = g_{{\bar{H}}}(x)/C_4(t)^{1/2}$.

### Example 8

*(Hill numbers and effective number of alleles.)* Let *q≥ 0* be a real-valued number. The Hill number of order *q* (Hill*q*) is a measure of diversity (Hill 1973; Jost 2006, 2010; Mergeay 2024) defined as

27 $$\begin{aligned} D_q(t) = h_q(S_{g_{\tiny \mathrm{Hill}q}}(t)) = \left\{ \begin{array}{ll} \left( \sum _{k=1}^{A_n(t)} x_k^q\right) ^{1/(1-q)}, & q \ne 1,\\ \exp \left( -\sum _{k=1}^{A_n(t)} x_k \log (x_k)\right) , & q=1 \end{array}\right. \end{aligned}$$

in generation *t*. It is a function

$$\begin{aligned} h_q(S) = \left\{ \begin{array}{ll} S^{1/(1-q)}, & q\ne 1,\\ \exp (S), & q=1 \end{array}\right. \end{aligned}$$

of the summary statistic $S=S_{g_{\tiny \mathrm{Hill}q}}(t)$ of the AFS in generation *t*, based on

$$\begin{aligned} g_{\mathrm{Hill}q}(x) = \left\{ \begin{array}{ll} x^q, & q \ne 1,\\ - x \log (x), & q=1. \end{array}\right. \end{aligned}$$

Note that $D_0(t)=A_n(t)$ is the number of alleles, also referred to as richness by Mergeay (2024), whereas $D_q(t)$ for *q>0* can be interpreted as an effective number of alleles. The rationale of this name is that $D_q(t)=A$ for an AFS with *A* equally frequent alleles (cf. (25)), regardless of the value of *q*. For an AFS with non-equal allele frequencies $D_q(t)$ upweights alleles with larger frequencies more, the higher the value of *q* is. Note also that $D_2(t) = A_e(t) = 1/[1-H(t)]$ is the most frequently used version of effective number of alleles (Crow and Kimura (1970), p. 323–324, Allendorf et al. (2024)), where $A_e$ is an acronym for “**e**ffective number of **A**lleles”. As a measure of diversity it is equivalent to (a strictly monotone transformation of) the gene diversity *H*(*t*). Hill numbers are frequently used in ecology, with $A_n(t)$ the number of species and $x_k$ the frequency of species *k*.

## The Dynamics of the Frequency of One Allele

In Sect. 5 we will study the dynamics of the expected allele frequency spectrum (EAFS). As a preparation it is helpful to first study the dynamics of the frequency *X*(*t*) of one single allele (say *a*). Assume that *a* is present in the population in generation 0, with a frequency *0<X(0)≤ 1*. It follows from the definitions of the reproduction cycles of Sects. 2.1 and 2.2 that *X*(*t*) is a Markov chain with a time-varying state space ${{{\mathcal {X}}}}(t)$, for monoecious as well as dioecious populations. Let

28 $$\begin{aligned} P_{xy}(t) = P(X(t+1)=y|X(t)=x) \end{aligned}$$

be the transition probability of this Markov chain from generation *t* to generation *t+1*, between states $x\in {{{\mathcal {X}}}}(t)$ and $y\in {{{\mathcal {X}}}}(t+1)$. We will give explicit formulas for these transition probabilities in Appendix A, separately for monoecious and dioecious populations. In absence of mutations (mutation probability *μ =0*), the process *X*(*t*) has two absorbing states 1 and 0, corresponding to *a* getting either fixed or lost. When *μ>0*, because of properties of the infinite alleles model *X*(*t*) has one single absorbing state 0, since eventually *a* will be lost with probability 1. It follows from the calculations of Appendices A.1 and A.2 that

29 $$\begin{aligned} E[X(t+1)|X(t)=x] = \sum _{y\in {{{\mathcal {X}}}}(t+1)} yP_{xy}(t) = (1-\mu )x \end{aligned}$$

for one- and two-sex populations. This means that on average a fraction *1-μ* of the gene copies with allele *a* is lost in each generation. The variance of the allele frequency process satisfies

30 $$\begin{aligned} \begin{array}{rcl} \text{ Var }[X(t+1)|X(t)=x] & =& \sum _{y\in {{{\mathcal {X}}}}(t+1)} [y-(1-\mu )x]^2 P_{xy}(t)\\ & \approx & [1-d(t)] \frac{(1-\mu )^2x(1-x)}{2N_e(t)} + d(t+1)\frac{x(1-\mu )[1-x(1-\mu )]}{2N_e(t+1)}, \end{array} \end{aligned}$$

where

31 $$\begin{aligned} d(t) = \frac{N_e(t)}{N(t)} \end{aligned}$$

is the ratio between the effective and census sizes at time *t*. Exact variance formulas that motivate (30) are provided in Appendices A.1 and A.2. Formula (30) implies that if the mutation probability *μ* is small, the variance effective size $N_{eV}(t)=x(1-x)/\{2\mathrm{Var}[X(t+1)|X(t)=x]\}$ of the population at time *t* satisfies

32 $$\begin{aligned} \frac{1}{2N_{eV}(t)} \approx \frac{1-d(t)}{2N_e(t)} + \frac{d(t+1)}{2N_e(t+1)}. \end{aligned}$$

This reveals that $N_{eV}(t)$ is essentially a weighted harmonic average of $N_e(t)$ and $N_e(t+1)$ when *d(t)=d* stays constant. In particular, the variance effective size lags the haploid inbreeding effective size by one generation ($N_{eV}(t)=N_e(t+1)$) for a Wright-Fisher model with constant *d(t)=d=1*, in agreement with Crow (1954). On the other hand, when *d*(*t*) varies with time we see from (32) that $N_{eV}(t)$ gets smaller and larger if *d(t+1)>d(t)* and *d(t+1)<d(t)*, respectively.

The choice of census size will effect the value of the variance effective size in (32) through *d*(*t*) and *d(t+1)*. If *N*(*t*) refers to the number of zygotes and *N*(*t*) is very large, then *d*(*t*) and *d(t+1)* are both small and $N_{eV}(t)\approx N_e(t)$. On the other hand, if *N*(*t*) is the number of juveniles before fertilization and additionally $N(t)\approx N_e(t)$, then *d*(*t*) and *d(t+1)* are both close to 1 and $N_{eV}(t)\approx N_e(t+1)$.

## Expected Allele Frequency Spectrum (EAFS)

Let

33 $$\begin{aligned} F_x(t) = E[f_x(t)|\boldsymbol{ f}(0)] \end{aligned}$$

refer to the expected number of alleles in generation *t* with frequency *x*. The corresponding expected allele frequency spectrum (EAFS) of generation *t* is a row vector

34 $$\begin{aligned} \boldsymbol{ F}(t) = E[\boldsymbol{ f}(t)|\boldsymbol{ f}(0)] = (F_x(t); \, x\in {{{\mathcal {X}}}}(t)). \end{aligned}$$

We will study the dynamics of *F*(*t*) when *t* increases. Based on work of Kimura (1964); Nei and Li (1976) used Kolmogorov’s forward equation to derive a diffusion approximation of *F*(*t*) for a constant and large population. Watterson (1984) derived formulas that can be used to compute $\boldsymbol{ F}(t)$ for a constant and large population, based on coalescence theory. Here we will rather use exact methods, which makes it possible to derive $\boldsymbol{ F}(t)$ for general population size trajectories. Because of linearity of the expectation operator, a linear recursion of $\boldsymbol{ F}(t)$ can be derived from the transition probabilities (28) of the allele frequency process of a single allele. In more detail we have that

35 $$\begin{aligned} F_y(t+1) = \sum _{x\in {{{\mathcal {X}}}}(t)} F_x(t)P_{xy}(t) + 2\theta (t+1) 1(y=\varepsilon (t+1)), \end{aligned}$$

for any $y\in {{{\mathcal {X}}}}(t+1)$, with

36 $$\begin{aligned} \theta (t) = N(t)\mu \end{aligned}$$

a rescaled mutation rate that reflects a balance between the mutation rate and census size, whereas

37 $$\begin{aligned} \varepsilon (t) = \frac{1}{2N(t)}. \end{aligned}$$

The first and second terms on the right-hand side of (35) refer to the expected number of alleles of generation *t+1* with frequency *y*, that were or were not present in generation *t*, respectively. Note in particular, because of properties of the infinite alleles model, that each new allele of generation *t+1* is only present in one gene copy of this generation, with frequency *\varepsilon (t+1)*.

A time recursion similar to (35)-(36) appears in Evans et al. (2007) for the expected genomewide allele frequency spectrum, where $F_x(t)$ corresponds to the expected number of *biallelic* loci with frequency *x* of a derived allele. In contrast, our definition of $F_x(t)$ refers to the expected number of alleles with frequency *x*, at one single *polymorphic* locus. Additionally, we consider genetic models that allow for separate census and effective size, and give a unified treatment of monoecious and dioecious populations.

The matrix version of recursion (35) is

38 $$\begin{aligned} \boldsymbol{ F}(t+1) = \boldsymbol{ F}(t)\boldsymbol{ P}(t) + 2\theta (t+1)\boldsymbol{ \delta }_{\varepsilon (t+1)}(t+1), \end{aligned}$$

where

39 $$\begin{aligned} \boldsymbol{ P}(t) = (P_{xy}(t); \, x\in {{{\mathcal {X}}}}(t), y\in {{{\mathcal {X}}}}(t+1)) \end{aligned}$$

is the *(2N(t)+1)× (2N(t+1)+1)* transition matrix between generations *t* and *t+1* of the single allele Markov process (28), whereas

40 $$\begin{aligned} \boldsymbol{ \delta }_x(t) = \{1(y=x); \, y\in {{{\mathcal {X}}}}(t)\}, \end{aligned}$$

for each $x\in {{{\mathcal {X}}}}(t)$, is a row vector of length *2N(t)+1* with a 1 in position *x* and zeros elsewhere. Repeated application of (38) gives an explicit formula

41 $$\begin{aligned} \boldsymbol{ F}(t) = \boldsymbol{ f}(0) \boldsymbol{ P}(0;t) + 2\sum _{s=1}^t \theta (s) \boldsymbol{ \delta }_{\varepsilon (s)}\boldsymbol{ P}(s;t) \end{aligned}$$

of the EAFS at time *t*, where

$$\begin{aligned} \boldsymbol{ P}(s;t) = \prod _{r=s}^{t-1} \boldsymbol{ P}(r) = (P_{xy}(s;t);x\in {{{\mathcal {X}}}}(s),y\in {{{\mathcal {X}}}}(t)) \end{aligned}$$

is the collection of transition probabilities

$$\begin{aligned} P_{xy}(s;t) = P(X(t)=y|X(s)=x) \end{aligned}$$

of the allele frequency process *X(· )* between generations *s* and *t*.

## Expected Summary Statistics

Recall from (5) that the summary statistic $S_g(t)$ of the AFS in generation *t* based on function *g*, is a linear function of the elements of $\boldsymbol{ f}(t)$. From this equation, (33)-(34) and linearity of the expectation operator it follows that the corresponding expected summary statistic in generation *t* is

42 $$\begin{aligned} E[S_g(t)] = \sum _{x\in {{{\mathcal {X}}}}(t)} F_x(t)g(x) = \boldsymbol{ F}(t)\boldsymbol{ g}(t), \end{aligned}$$

with $\boldsymbol{ g}(t)$ the column vector of length *2N(t)+1* defined in (6). Insertion of (41) into (42) yields an explicit expression

43 $$\begin{aligned} \begin{array}{rcl} E[S_g(t)] & =& \boldsymbol{ f}(0) \boldsymbol{ P}(0;t) \boldsymbol{ g}(t)+ 2\sum _{s=1}^t \theta (s) \boldsymbol{ \delta }_{\varepsilon (s)}\boldsymbol{ P}(s;t) \boldsymbol{ g}(t)\\ & =& \sum _{x\in {{{\mathcal {X}}}}(0)} f_x(0) E\{g[X(t)]|X(0)=x\}\\ & +& 2\sum _{s=1}^t \theta (s) E\{g[X(t)]|X(s)=\varepsilon (s)\} \end{array} \end{aligned}$$

for the expected summary statistic of the AFS, based on *g*, in generation *t*. In the last step of (43) we made use of the fact that $\boldsymbol{ P}(s;t)$ is the *(t-s)*-step transition matrix of the Markov process *X(· )* between time points *s* and *t*. The recursive formulas (35) and (38) for the EAFS give rise to a corresponding recursion

44 $$\begin{aligned} \begin{array}{rcl} E[S_g(t+1)] & =& \sum _{x\in {{{\mathcal {X}}}}(t)} F_x(t) E\{g[X(t+1)]|X(t)=x\} + 2\theta (t+1) g(\varepsilon (t+1))\\ & =& \boldsymbol{ F}(t)\boldsymbol{ P}(t)\boldsymbol{ g}(t) + 2\theta (t+1) g(\varepsilon (t+1)) \end{array} \end{aligned}$$

for the expected summary statistic.

### Example 9

*alleles that have been present at some time up to generationt, increases(Expected total allele frequency.)* Let $g_{\mathrm{id}}(x)=x$ be the identity function defined in Example 2. It is clear from (12) that the expected total allele frequency in generation *t* is

45 $$\begin{aligned} E[S_{g_{\tiny \mathrm{id}}}(t)]= \sum _{x\in {{{\mathcal {X}}}}(t)} x F_x(t) = 1. \end{aligned}$$

Equation (45) can also be deduced recursively from the time recursion (29) of the expected allele frequency of one single allele. Indeed, since (29) is equivalent to

46 $$\begin{aligned} \boldsymbol{ P}(t)\boldsymbol{ g}_{\mathrm{id}}(t) = (1-\mu )\boldsymbol{ g}_{\mathrm{id}}(t), \end{aligned}$$

and if $E[S_{g_{\tiny \mathrm{id}}}(t)]=1$ is assumed, it follows from (44) that

47 $$\begin{aligned} \begin{array}{rcl} E[S_{g_{\tiny \mathrm{id}}}(t+1)] & =& \boldsymbol{ F}(t)\boldsymbol{ P}(t)\boldsymbol{ g}_{\mathrm{id}}(t) + 2\theta (t+1) g_{\mathrm{id}}(\varepsilon (t+1))\\ & =& (1-\mu )\boldsymbol{ F}(t)\boldsymbol{ g}_{\mathrm{id}}(t) + 2\theta (t+1) \varepsilon (t+1)\\ & =& (1-\mu )E[S_{g_{\tiny \text{id }}}(t)]+ \mu \\ & =& 1. \end{array} \end{aligned}$$

    

### Example 10

*(Expected number of alleles.)* In order to find expressions for the expected number $E[A_n(t)]$ of distinct alleles in generation *t*, we apply formula (43) with $g(x)=g_{A_n}(x)$ given by (9). To this end, we make use of the fact that $g_{A_n}(x)=1-1(x=0)$. Insertion into the lower part of (43) yields

48 $$\begin{aligned} E[A_n(t)] = \sum _{x\in {{{\mathcal {X}}}}(0)} f_x(0)[1-P_{x0}(0;t)] + 2\sum _{s=1}^t \theta (s) [1-P_{\varepsilon (s)0}(s;t)]. \end{aligned}$$

Recall that the Markov chain *X(· )* has one absorbing state 0 when *μ>0*. Consequently, in this case the terms $1-P_{x0}(0;t)$ and $1-P_{\varepsilon (s)0}(s;t)$ that appear in (48) are non-absorption probabilities of this Markov chain. The first sum on the right hand side of (48) corresponds to the expected number of alleles, present in generation 0, that have not yet vanished (have not been absorbed by state 0) at time *t*, whereas the second sum on the right hand side of (48) is the expected number of mutations after generation 0 that still remain (have not yet been absorbed by state 0) at time *t*. In absence of mutations (*μ =0*), formula (48) reduces to

49 $$\begin{aligned} E[A_n(t)] {\mathop {=}\limits ^{\mu =0}} \sum _{x\in {{{\mathcal {X}}}}(0)} f_x(0)[1-P_{x0}(0;t)]. \end{aligned}$$

Since *X(· )* has two absorbing states 0 and 1 when *μ =0*, $P_{x0}(0;t)$ is still the absorption probability of state 0, but $1-P_{x0}(0;t)$ is no longer a non-absorption probability. For a monoecious Wright-Fisher model ($N_e(t)=N(t)$), the absorption probability of state 0 in one generation is $P_{x0}(0;1)=P_{x0}(0)=(1-x)^{2N(1)}$. Then (49) simplifies to

50 $$\begin{aligned} E[A_n(1)] {\mathop {=}\limits ^{\mu =0}} \sum _{x\in {{{\mathcal {X}}}}(0)} f_x(0)[1 - (1-x)^{2N(1)}], \end{aligned}$$

a formula derived by Denniston (1978).

### Example 11

*(Expected gene diversity.)* The dynamics of expected gene diversity has been well studied, see for instance Wright (1931); Malécot (1948), and Nei et al. (1975). Here we will show that such dynamics can be retrieved from general properties of summary statistics of the EAFS. In more detail, in order to find the expected gene diversity *E*[*H*(*t*)] we make use of (44) with $g(x)=g_{H}(x)=x(1-x)$ as in (17). Consider at first the monoecious population of Section 2.1. Utilizing (29) and (A.11), we find that

51 $$\begin{aligned} \begin{array}{rcl} E\{X(t+1)[1-X(t+1)]|X(t)=x\} & =& (1-\mu )x[1-(1-\mu )x]\\ & -& [1-d(t)] c(t) \frac{(1-\mu )^2 x(1-x)}{2N_e(t)} - d(t+1) \frac{x(1-\mu )[1-x(1-\mu )]}{2N_e(t+1)}\\ & =& (1-\mu )\left( 1 - \frac{d(t+1)}{2N_e(t+1)}\right) \cdot x[1-(1-\mu )x] \\ & -& \frac{[1-d(t)] c(t) (1-\mu )^2}{2N_e(t)} \cdot x(1-x), \end{array} \end{aligned}$$

with *d*(*t*) and *c*(*t*) defined in (31) and (A.12), respectively. Insertion into (44) yields

52 $$\begin{aligned} \begin{array}{rcl} E[H(t+1)] & =& (1-\mu )\left( 1 - \frac{d(t+1)}{2N_e(t+1)}\right) \sum _{x\in {{{\mathcal {X}}}}(t)} F_x(t) x[1-(1-\mu )x]\\ & -& \frac{[1-d(t)] c(t) (1-\mu )^2}{2N_e(t)} \sum _{x\in {{{\mathcal {X}}}}(t)} F_x(t) x(1-x)\\ & +& 2\theta (t+1)\varepsilon (t+1)[1-\varepsilon (t+1)]\\ & =& (1-\mu )\left( 1 - \frac{d(t+1)}{2N_e(t+1)}\right) (\mu + (1-\mu )(1-E[H(t)]))\\ & -& \frac{[1-d(t)] c(t) (1-\mu )^2}{2N_e(t)} E[H(t)] + \mu [1-\varepsilon (t+1)]\\ & =& \left( 1 - \frac{d(t+1)}{2N_e(t+1)} - \frac{[1-d(t)] c(t)}{2N_e(t)} \right) (1-\mu )^2 E[H(t)] \\ & +& \mu \left[ (1-\mu ) ( 1 - \frac{1}{2N(t+1)}) + 1-\frac{1}{2N(t+1)}\right] \\ & =:& e(t+1) (1-\mu )^2 E[H(t)] + h(t+1)\mu (2-\mu ), \end{array} \end{aligned}$$

where

53 $$\begin{aligned} \begin{array}{rcl} e(t+1) & =& 1 - \frac{d(t+1)}{2N_e(t+1)} - \frac{[1-d(t)] c(t)}{2N_e(t)},\\ h(t+1) & =& 1 - \frac{1}{2N(t+1)} \end{array} \end{aligned}$$

are factors close to 1. Repeated application of (52) yields

54 $$\begin{aligned} \begin{array}{rcl} E[H(t)] & =& (1-\mu )^{2t} \prod _{s=1}^t e(s) \cdot H(0)\\ & +& \mu (2-\mu ) \sum _{s=1}^t h(s) (1-\mu )^{2(t-s)} \prod _{r=s+1}^t e(r). \end{array} \end{aligned}$$

Expected gene diversity formulas are analogous for a dioecious population. The only difference is the expression for *e(t+1)* in formula (53), where *d*(*t*) is replaced by $\tilde{d}(t)$, defined in (A.27).

## Equilibrium

Consider a population for which the census and effective sizes *N(t)=N* and $N_e(t)=N_e$ are constant over time. Let also ${{{\mathcal {X}}}}$, $\boldsymbol{ g}$, $\boldsymbol{ P}$, $\boldsymbol{ \delta }_x$ and *\varepsilon* refer to the constant values of ${{{\mathcal {X}}}}(t)$, $\boldsymbol{ g}(t)$, *\varepsilon (t)*, $\boldsymbol{ P}(t)$, and $\boldsymbol{ \delta }_x(t)$ in equations (3), (6), (37), (39), and (40). The frequency process *X(· )* of a single allele is now a stationary Markov process with state space ${{{\mathcal {X}}}}$ and transition matrix $\boldsymbol{ P}$. For a constant population, the expected value (41) of the AFS in generation *t*, and of its summary statistic $S_g(t)$ in (42), simplify to

55 $$\begin{aligned} \boldsymbol{ F}(t) = \boldsymbol{ f}(0) \boldsymbol{ P}^t + 2\theta \boldsymbol{ \delta }_\varepsilon \sum _{s=0}^{t-1} \boldsymbol{ P}^s \end{aligned}$$

and

56 $$\begin{aligned} E[S_g(t)]= \boldsymbol{ f}(0) \boldsymbol{ P}^t \boldsymbol{ g}+ 2\theta \boldsymbol{ \delta }_\varepsilon \sum _{s=0}^{t-1} \boldsymbol{ P}^s \boldsymbol{ g}, \end{aligned}$$

respectively.

### Example 12

*(Total number of alleles.)* Recall from Example 4 that the total number $A_{n\mathrm{tot}}(t)$ of alleles that are present, or have been lost, at time *t*, is a summary statistic of the AFS based on $g_1(x)=1$. Since $\boldsymbol{ P}$ is the transition matrix of a Markov chain its row sums equal 1, and consequently $\boldsymbol{ P}\boldsymbol{ g}_1 = \boldsymbol{ g}_1$. It follows from (56), and the identity $\boldsymbol{ \delta }_\varepsilon \boldsymbol{ g}_1=1$, that

$$\begin{aligned} E[A_{n\mathrm{tot}}(t)] = \boldsymbol{ f}(0)\boldsymbol{ g}_1 + 2\theta \boldsymbol{ \delta }_\varepsilon t \boldsymbol{ g}_1 = A_n(0) + 2\theta t. \end{aligned}$$

Hence the expected number of alleles that have been present at some time up to generation *t*, increases linearly with *t* at rate $2\theta = 2N\mu$.

We will look at the limits of (55) and (56) when $t\rightarrow \infty$ and *μ>0*, corresponding to an equilibrium between genetic drift and mutation. It is clear from Example 12 that these limits will not exist when lost alleles are taken into account. The limit of $F_x(t)$ in (55) only exists for frequences $x\in {{{\mathcal {X}}}}$ with *x>0*, and the limit of $E[S_g(t)]$ in (56) only exists for functions *g* such that *g(0)=0*.

### Example 13

*(Equilibrium EAFS.)* For any row vector $\boldsymbol{ v}= (v_x; \, x\in {{{\mathcal {X}}}})$ denote by $\boldsymbol{ v}_{(-0)} = (v_x; x\in {{{\mathcal {X}}}}, x>0)$ the subvector of $\boldsymbol{ v}$ that excludes its first component $v_0$. In particular,

57 $$\begin{aligned} \boldsymbol{ F}_{(-0)}(t) = (F_x(t); \, x\in {{{\mathcal {X}}}}, x>0) \end{aligned}$$

refers to the EAFS without lost alleles. The equilibrium EAFS is obtained by taking the $t\rightarrow \infty$ limit in (57). When *μ =0*, no new alleles arise through mutations and eventually one single allele gets fixed as $t\rightarrow \infty$, i.e.

58 $$\begin{aligned} \boldsymbol{ F}_{(-0)}(\infty ) = (F_x(\infty ); \, x\in {{{\mathcal {X}}}}, x>0) {\mathop {=}\limits ^{\mu =0}} \boldsymbol{ \delta }_{1(-0)}, \end{aligned}$$

with $\boldsymbol{ \delta }_{1(-0)}=(0,\ldots ,0,1)$ a row vector of length 2*N* with zeros in the first *2N-1* positions and a one in the last position. Formula (58) follows by taking the $t\rightarrow \infty$ limit for the first term of (55), first making use of the fact that $((\boldsymbol{ P}^t)_{xy}; \, y\in {{{\mathcal {X}}}}, y>0) \rightarrow x\boldsymbol{ \delta }_{1(-0)}$, and then combining the effects of different allele frequencies *x* of generation 0, making use of (12). When *μ>0* only the second term of (55) contributes as $t\rightarrow \infty$, with a limiting equilibrium EAFS that equals

59 $$\begin{aligned} \boldsymbol{ F}_{(-0)}(\infty ) &= (F_x(\infty ); \, x\in {{{\mathcal {X}}}}, x>0) {\mathop {=}\limits ^{\mu>0}} 2\theta \sum _{t=0}^\infty \left( \boldsymbol{ \delta }_\varepsilon \boldsymbol{ P}^t \right) _{(-0)} \nonumber \\ & = 2\theta \boldsymbol{ \delta }_{\varepsilon (-0)} (\boldsymbol{ I}-\boldsymbol{ P}_{(-0)})^{-1}. \end{aligned}$$

Here $\boldsymbol{ \delta }_{\varepsilon (-0)} = (1,0,\ldots ,0)$ is a row vector of length 2*N* with a one in the first position (corresponding to allele frequency *\varepsilon =1/(2N)*) and zeros elsewhere, $\boldsymbol{ P}_{(-0)}=(P_{xy}, x,y\in {{{\mathcal {X}}}}\setminus \{0\})$ is the submatrix of $\boldsymbol{ P}$ corresponding to non-lost alleles, and $\boldsymbol{ I}$ is the identity matrix of order 2*N*.

We will assume that the transition matrix of *X(· )* admits a spectral representation

60 $$\begin{aligned} \boldsymbol{ P}= \sum _{q=0}^{2N} \lambda _q \boldsymbol{ r}_q \boldsymbol{ l}_q, \end{aligned}$$

where $\lambda _q$ is the *(q+1)*th largest eigenvalue of $\boldsymbol{ P}$, with left and right eigenvectors $\boldsymbol{ l}_q=(l_{q0},\ldots ,l_{q,2N})$ and $\boldsymbol{ r}_q=(r_{q0},\ldots ,r_{q,2N})^\prime$, respectively. It is assumed in (60) that the left and right eigenvectors are normalized so that $\boldsymbol{ l}_q \boldsymbol{ r}_q=1$, and moreover $\boldsymbol{ l}_q \boldsymbol{ r}_m = 0$ for *q≠ m*. Since *μ>0*, we have a unique stationary distribution $\boldsymbol{ l}_0=\boldsymbol{ \delta }_0=(1,0,\ldots ,0)$, with eigenvalue

61 $$\begin{aligned} \lambda _0=1, \end{aligned}$$

and a corresponding right eigenvector $\boldsymbol{ r}_0=\boldsymbol{ g}_1=(1,\ldots ,1)^\prime$. The other eigenvalues are listed in decreasing order $1>\lambda _1> \lambda _2> \ldots> \lambda _{2N}>0$. Inserting (60) into (59) we find that

62 $$\begin{aligned} \boldsymbol{ F}_{(-0)}(\infty ) = 2\theta [\boldsymbol{ \delta }_{\varepsilon (-0)} + \sum _{q=1}^{2N} \frac{\lambda _q}{1-\lambda _q} (\boldsymbol{ \delta }_\varepsilon \boldsymbol{ r}_q) \boldsymbol{ l}_{q(-0)}], \end{aligned}$$

where $\boldsymbol{ l}_{q(-0)}$ is the *q*th left eigenvector of $\boldsymbol{ P}$ without its first component $\boldsymbol{ l}_{q0}$. The eigenvalues of a monoecious population satisfy

63 $$\begin{aligned} \lambda _q = (1-\mu )^q \prod _{k=1}^{q-1} \left( 1-\frac{k}{2N_e}\right) \end{aligned}$$

for *q=1,2,… 2N* (see for instance equation (3.40) of Ewens (2004)). Indeed, it follows from (29) and (46) that $\boldsymbol{ r}_1=\boldsymbol{ g}_{\mathrm{id}}$ (or $r_1(x)=x$) is the right eigenvector of the second largest eigenvalue

64 $$\begin{aligned} \lambda _1 = 1-\mu . \end{aligned}$$

This is the long term multiplicative rate at which alleles vanish from the populaton, whereas $\boldsymbol{ l}_{1(-0)}$, appropriately normalized, is the quasi equilibrium distribution, i.e. the distribution of an a allele that has been around (not been lost) in the population for a long time. Generally, the right eigenvector $\boldsymbol{ r}_q$ of the *(q+1)*th largest eigenvalue corresponds to a polynomial $r_q$ of degree *q*, with $r_q(0)=0$ for *q>0*. If follows from (44) that the summary statistic of the EAFS based on $\boldsymbol{ r}_q$ satisfies the recursion

65 $$\begin{aligned} E[S_{r_q}(t+1)] = \lambda _q E[S_{r_q}(t)] + 2\theta r_q(\varepsilon ). \end{aligned}$$

The simple form of (65) suggests using a summary statistic of the AFS based on $\boldsymbol{ r}_q.$ However, $\boldsymbol{ r}_q$ will generally have quite a complicated form, depending on *μ*, *N* and $N_e$. For instance, it is shown in Appendix B.1 that the right eigenvector $\boldsymbol{ r}_2$ associated with the third largest eigenvalue

66 $$\begin{aligned} \lambda _2=(1-\mu )^2\left( 1-\frac{1}{2N_e}\right) \end{aligned}$$

corresponds to any second order polynomial proportional to $r_2(x) = x^2 + cx$, with *c* a function of *μ*, *N*, and $N_e$ defined in (B.5).  

### Example 14

*(Diffusion approximation of EAFS)* When $N_e$ is large and *μ>0* is small, it is possible to use diffusion processes and Kolmogorov’s forward equation to find explicit approximations of the components of (59). Based on work of Wright (1938), Kimura and Crow (1964) found that

67 $$\begin{aligned} F_x(\infty ) \approx F_x^{\mathrm{appr}}(\infty ) = \frac{1}{2N} \cdot 4\theta _e (1-x)^{4\theta _e - 1} x^{-1} \end{aligned}$$

for $$x\in {{{\mathcal {X}}}}\setminus \{0\}$$, with

68 $$\begin{aligned} \theta _e = N_e\mu \end{aligned}$$

a variable that quantifies a balance between genetic drift and mutation (see also formula 9.6.11 of Crow and Kimura (1970), p. 455). It is shown in Hössjer et al. (2016) that for a Wright-Fisher model ($N=N_e$) there is a diffusion approximation

69 $$\begin{aligned} \lambda _q \approx \lambda _q^{\mathrm{appr}} = \exp \left[ -\frac{q(q-1+4\theta _e)}{4N_e}\right] =\exp \left[ -\frac{q(q-1)}{2}\cdot \frac{1}{2N_e} - q\mu \right] \end{aligned}$$

of the eigenvalues in (60), for *q=0,1,2,…*. Note that (69) aproximates (63) when *μ>0* is small and $N_e$ is large.

### Example 15

*(Equilibrium expected number of alleles.)* By letting $t\rightarrow \infty$ in (48) or (56), it follows that the equilibrium expected number of alleles is

70 $$\begin{aligned} E[A_n(\infty )] = 2\theta \sum _{t=0}^\infty (1-P^t_{\varepsilon 0}) = 2\theta E(T|X(0)=\varepsilon ) \end{aligned}$$

when *μ>0*, with $P_{xy}^t=P(X(t)=y|X(0)=x)$ the *t* step transition probability of the stationary Markov process *X(· )* between *x* and *y*, whereas *T* is the extinction time of an allele that is present in the population at time 0, with frequency *X*(0) (see Ewens (1964), for a special case of (70) for a Wright-Fisher model). Hence, $E[A_n(\infty )]$ is the rescaled mutation rate *θ* times the expected exinction time of an allele that exists in one single copy in generation 0. We may also use Ewens’ sampling formula (Ewens 1972) to derive an approximation

71 $$\begin{aligned} E[A_n(\infty )] \approx 4\theta _e\sum _{k=0}^{2N-1} \frac{1}{4\theta _e+ k} \end{aligned}$$

of the expected number of alleles at equilibrium, for a monoecious population. Ewen’s sampling formula is based on a large population assumption, and it can be proved using coalescence theory (see for instance Theorem 1.11 of Durrett (2008)). Note that (70)-(71) provide an explicit, approximate formula for the expected extinction time

72 $$\begin{aligned} E[T|X(0)=\varepsilon ] \approx \frac{2N_e}{N} \sum _{k=0}^{2N-1} \frac{1}{4\theta _e+ k} \end{aligned}$$

of an allele that is present in one single copy of generation 0. From this it follows that $E[T|X(0)=\varepsilon ]\rightarrow \infty$ as $\mu \rightarrow 0$. The reason is that for small *μ>0*, there is a small probability of order 1/(2*N*) that *X(· )* gets trapped, for a long time, in the semi-absorbing state 1, before it eventually goes extinct.

A diffusion approximation

73 $$\begin{aligned} E[A_n(\infty )] = \sum _{\begin{array}{c} x\in {{{\mathcal {X}}}}\\ x>0 \end{array}} F_x(\infty ) \approx 4\theta _e \int _{1/(2N)}^1 (1-x)^{4\theta _e - 1} x^{-1}dx \end{aligned}$$

of (70) is obtained from (67). It is also based on a large population assumption, and an integral approximation of the sum that defines $E[A_n(\infty )]$ (cf. page 455 of Crow and Kimura (1970)). Ewens (1964) establishes (73), for a Wright-Fisher model, through (70) and an approximation of *E[T|X(0)=\varepsilon ]* that is slightly different from (72).

By changing the lower and upper limits of integration respectively in (73), to *η*, we obtain asymptotic expressions for the expected number or common and rare alleles (cf. Example 3) at equilibrium, which in turn makes it possible to find approximations for the expected average frequency $E[{\bar{x}}(\infty )]$ of rare alleles at equilibrium.

In Appendix B.2 we establish some properties of $E[A_n(\infty )]$ and $E[{\bar{x}}(\infty )]$, based on the two approximate formulas (71) and (73). In particular, it is shown that these two methods of deriving $E[A_n(\infty )]$ and $E[{\bar{x}}(\infty )]$ are consistent for large populations, as expected

### Example 16

*(Equilibrium expected gene diversity)* In order to find the equilibrium expected gene diversity *E[H(∞ )]* of a monoecious population we start by simplifying recursion (52) for the *E*[*H*(*t*)] when the population is constant. It can be seen from (31), (53) and (A.12) that *c(t)=1*, *d(t)=d(t+1)* and

74 $$\begin{aligned} \begin{array}{rcl} e(t+1) & =& 1 - 1/(2N_e),\\ f(t+1) & =& 1 - 1/(2N). \end{array} \end{aligned}$$

Insertion into (52) yields a recursion

75 $$\begin{aligned} E[H(t+1)] = \left( 1-\frac{1}{2N_e}\right) (1-\mu )^2 E[H(t)] + \mu (2-\mu ) \left( 1-\frac{1}{2N}\right) \end{aligned}$$

for the expected gene diversity of a constant population. It follows from (63) that *E*[*H*(*t*)] decreases at rate $\lambda _2$ in (66), in each generation, due to drift and mutation of old alleles (the first term of (75)). At equilibrium, this is balanced by an increased gene diversity due to new alleles (the second term of (75)). Letting $t\rightarrow \infty$ in (75) we obtain an explicit expression

76 $$\begin{aligned} E[H(\infty )] &= \frac{\mu (2-\mu ) \left( 1-\frac{1}{2N}\right) }{1 - \left( 1-\frac{1}{2N_e}\right) (1-\mu )^2} = \frac{4N_e\mu \left( 1-\frac{\mu }{2}\right) \left( 1-\frac{1}{2N}\right) }{1 + 4N_e\mu - \mu (2N_e\mu + 2 -\mu )} \nonumber \\ & \approx \frac{4N_e \mu }{1 + 4N_e \mu } \end{aligned}$$

for the equilibrium expected gene diversity, where the approximation in the last step of (76) holds for large $N_e$ and small *μ*. Recall that the product $\theta _e=N_eu$ of (76) represents a balance between genetic drift and mutation. It influences the expected gene diversity at equilibrim so that it gets higher (lower) the larger the mutation rate *μ* (the rate $1/N_e(t)$ of genetic drift) is. Following the argument from page 455 of Crow and Kimura (1970), we find that the right-hand side of (76) is consistent with diffusion approximation (67), since

$$\begin{aligned} \begin{array}{rcl} E[H(\infty )] & =& \sum _{\begin{array}{c} x\in {{{\mathcal {X}}}}\\ x>0 \end{array}} x(1-x) F_x(\infty )\\ & \approx & \int _{1/(2N)} x(1-x) \cdot 4\theta _e (1-x)^{4\theta _e - 1} x^{-1}dx\\ & =& 4\theta _e\int _{1/(2N)} (1-x)^{4\theta _e}dx\\ & \approx & 4N_e\mu / (1 + 4N_e\mu ). \end{array} \end{aligned}$$

The equilibrium expected modified gene diversity, $E[\tilde{H}(\infty )]$, can be obtained by a direct argument, without making use of the EAFS. We have that

77 $$\begin{aligned} E[\tilde{H}(\infty )] = \left( 1-\frac{1}{2N_e}\right) (1-\mu )^2 E[\tilde{H}(\infty )] + \mu (2-\mu ). \end{aligned}$$

Indeed, if two gene copies are drawn randomly from a population in equilibrium, the probability that they have different alleles is $E[\tilde{H}(\infty )]$, the left-hand size of (77). The right-hand side of (77) is obtained by conditioning on whether none of these two gene copies mutated (probability $(1-\mu )^2$) or at least one of the two gene copies mutated (probability $\mu (2-\mu )$). If none of the two gene copies mutated, they have different alleles if they descend from different breeding gene copies (probability $1-1/(2N_e)$) and additionally, these two breeding gene copies have different alleles (probability $E[\tilde{H}(\infty )]$). If at least one of the two gene copies mutated, they have different alleles with probability 1, due to properties of the infinite-alleles model. See also page 323 of Crow and Kimura (1970) for a similar argument. If follows from (77) that the expected equilibrium of the modified gene diversity equals

78 $$\begin{aligned} E[\tilde{H}(\infty )] = \frac{4N_e\mu (1-\frac{\mu }{2})}{1 + 4N_e\mu - \mu (2N_e\mu + 2 -\mu )} \approx \frac{4N_e \mu }{1 + 4N_e \mu } \end{aligned}$$

Note that (77) and (78) are equivalent to (75) and (76), respectively, since gene diversity of a constant populaton is obtained by multiplying the modified gene diversity with *1-1/(2N)*, cf. (18).

Formulas for the equilibrium expected gene diversity are analogous in a dioecious population. For instance, the derivation of the expected gene diversity of a dioecious population only differs in that

$$\begin{aligned} e(t+1) = 1 - \frac{1}{2N_e} \left( 1-\frac{1}{2N-1}\right) \end{aligned}$$

replaces the expression for *e(t+1)* in (74). Consequently,

79 $$\begin{aligned} \begin{array}{rcl} E[H(\infty )] & =& \frac{\mu (2-\mu )(1-\frac{1}{2N})}{1-[1-\frac{1}{2N_e}\left( 1-\frac{1}{2N-1}\right) ](1-\mu )^2}\\ & =& \frac{4N_e \mu (1-\frac{\mu }{2})(1-\frac{1}{2N})}{1 + 4N_e\mu - \frac{1}{2N-1} - \mu [2N_e\mu + \left( 1-\frac{1}{2N-1}\right) (2-\mu )]}\\ & \approx & \frac{4N_e\mu }{1+4N_e\mu }. \end{array} \end{aligned}$$

The expected modified gene diversity of a dioecious population satisifies a recursion

80 $$\begin{aligned} E[\tilde{H}(\infty )] = \left[ 1-\frac{1}{2N_e}\left( 1-\frac{1}{2N-1}\right) \right] (1-\mu )^2 E[\tilde{H}(\infty )] + \mu (2-\mu ) \end{aligned}$$

that is slightly different from (80), in that the expression for drawing two gene copies that do not mutate and have different alleles, is different. Indeed, when two gene copies are drawn, without replacement, the probability that they descend from different parental gene copies is

81 $$\begin{aligned} & Q = 1 - \left[ \frac{1}{2}\cdot \frac{N-1}{2N-1}\cdot \frac{1}{2N_{ef}} + \frac{1}{2}\cdot \frac{N-1}{2N-1}\cdot \frac{1}{2N_{em}}\right] \nonumber \\ & \quad = 1 - \frac{1}{2N_e}\left( 1-\frac{1}{2N-1}\right) , \end{aligned}$$

where in the last step we made use of (2). Therefore, the probabiliy that none of the two gene copies mutates, and they have different alleles, is $(1-\mu )^2 Q E[\tilde{H}(\infty )]$ in equilibrium, in agreement with the first term on the right-hand side of (80). Solving the recursion (80) we find that the equilibrium expected modified gene diversity of a dioecious population is $[1-1/(2N)]^{-1}$ times the expression for the equilibrium expected gene diversity (79), as it should.

### Example 17

*(Equilibrium triplet gene diversity.)* We will find an expression for the expected triplet modified gene diversity $E[\tilde{H}_3(\infty )]$. Recall from Example 6 that this quantity is only defined for *N>1*. It is the probability that three gene copies, drawn at random from a population in equilibrium without replacement, have different alleles. For a monecious population, in analogy with (77) we obtain a recursion

82 $$\begin{aligned} \begin{array}{rcl} E[\tilde{H}_3(\infty )] & =& (1-\mu )^3 \left( 1-\frac{1}{2N_e}\right) \left( 1-\frac{2}{2N_e}\right) E[\tilde{H}_3(\infty )]\\ & +& 3\mu (1-\mu )^2 \left( 1-\frac{1}{2N_e}\right) E[\tilde{H}(\infty )]\\ & +& \mu ^2(3-2\mu ), \end{array} \end{aligned}$$

where on the right-hand side we conditioned on whether none, exactly one or at least two of the three gene copies, that were drawn from the equilibrium population, mutate. It follows from (63) and (82) that $\lambda _3$ is the rate at which the expected triplet modified gene diversity decreases due to drift and mutation of old alleles, and at equilibrium this is balanced by emergence of new alleles. Solving for $E[\tilde{H}_3(\infty )]$ we find that

83 $$\begin{aligned} \begin{array}{rcl} E[\tilde{H}_3(\infty )] & =& \frac{3\mu (1-\mu )^2 \left( 1-\frac{1}{2N_e}\right) E[\tilde{H}(\infty )] + \mu ^2(3-2\mu )}{1 - (1-\mu )^3 \left( 1-\frac{1}{2N_e}\right) (1-\frac{2}{2N_e})}\\ & \approx & \frac{3\mu E[\tilde{H}(\infty )]}{3\mu + \frac{3}{2N_e}}\\ & \approx & \frac{2N_e\mu }{1+2N_e\mu } \cdot \frac{4N_e \mu }{1+4N_e\mu } \end{array} \end{aligned}$$

where in the second step we ignored all terms that are asymptotically negligible in the limit of small *μ* and $1/N_e$, and in the last step we utilized (78). In order to show that (83) is consistent with the diffusion approximation (67), we make use of (22). This gives rise to an approximation

84 $$\begin{aligned} \begin{array}{rcl} E[\tilde{H}_3(\infty )] & =& \frac{1}{(1-\frac{1}{2N})(1-\frac{2}{2N})} \sum _{x\in {{{\mathcal {X}}}}; x>0} [x(1-x)^2 - x^2(1-x)] F_x(\infty )\\ & \approx & \sum _{x\in {{{\mathcal {X}}}}; x>0} [x(1-x)^2 - x^2(1-x)] F_x(\infty )\\ & \approx & \int _0^1 [x(1-x)^2 - x^2(1-x)] 4\theta _e (1-x)^{4\theta _e-1} x^{-1}dx\\ & =& 4\theta _e [\int _0^1 (1-x)^{4\theta _e+1}dx - \int _0^1 (1-x)^{4\theta _e} x dx]\\ & =& 4\theta _e [\frac{1}{2+4\theta _e} - \frac{1}{(2+4\theta _e)(1+4\theta _e)}]\\ & =& \frac{8\theta _e^2}{(1+2\theta _e)(1+4\theta _e)} \end{array} \end{aligned}$$

of the equilibrium value of the expected triplet modified gene diversity. Since $\theta _e$ is given by (68), this approximation agrees with the right-hand size of (83).

For a dioecious population, a recursion

85 $$\begin{aligned} E[\tilde{H}_3(\infty )] = (1-\mu )^3 Q_3 E[\tilde{H}_3(\infty )] + 3\mu (1-\mu )^2 Q E[\tilde{H}(\infty )] + \mu ^2(3-2\mu ), \end{aligned}$$

for the expected triplet modified gene diversity is found analogously to (82), with *Q* the probability (81) that two gene copies, drawn randomly without replacment, descend from different parental gene copies, whereas

$$\begin{aligned} \begin{array}{rcl} Q_3 & =& \frac{(N-1)(N-2)}{2(2N-1)(2N-2)} \left[ \left( 1-\frac{1}{2N_{ef}}\right) \left( 1-\frac{2}{2N_{ef}}\right) + \left( 1-\frac{1}{2N_{em}}\right) \left( 1-\frac{2}{2N_{em}}\right) \right] \\ & +& \frac{3N(N-1)}{2(2N-1)(2N-2)}\left( 2-\frac{1}{2N_{ef}}-\frac{2}{2N_{ef}}\right) \end{array} \end{aligned}$$

is the analogous probability when three gene copies are drawn without replacment. Inserting (78) into the right hand side of (85), we can solve this equation with respect to $E[\tilde{H}_3(\infty )]$, and obtain an explicit expression for the expected triplet modified gene diversity of a dioecous population.

## Sudden Population Size Change

In this section we will apply formulas from Section 5 to study how the expected value $E[S_g(t)]$ of a summary statistic of the AFS changes after a sudden population size change, with

86 $$\begin{aligned} N(t) = \left\{ \begin{array}{ll} N_0; & t\le 0,\\ N; & t>0, \end{array}\right. \end{aligned}$$

and

87 $$\begin{aligned} N_e(t) = \left\{ \begin{array}{ll} N_{e0}; & t\le 0,\\ N_e; & t>0, \end{array}\right. \end{aligned}$$

respectively. It can be seen from (86)-(87) that the population size change occurs after generation 0, with values $N_0$ ($N_{e0}$) and *N* ($N_e$) of the census (effective) size before and after the change. It is assumed that the ratio (31) between the effective and census sizes has the same value, before and after the population size change, so that

88 $$\begin{aligned} d = \frac{N_e(t)}{N(t)} \end{aligned}$$

does not depend on *t*. The mutation rate *μ* is not affected by the population size change. We will use short-hand notation

89 $$\begin{aligned} \boldsymbol{ P}(t) = \left\{ \begin{array}{ll} \boldsymbol{ P}_0=(P_{0xy}), & t<0,\\ \boldsymbol{ P}_{\mathrm{c}}=(P_{\mathrm{c}xy}), & t=0,\\ \boldsymbol{ P}=(P_{xy}), & t>0, \end{array}\right. \end{aligned}$$

for the transition matrices between two consequtive generations *t* and *t+1* of the Markov chain *X(· )*. Since the transition matrix between generations *t* and *t+1* depends on the population sizes of both generations *t* and *t+1*, it will depend on whether the census size stays at $N_0$ (index 0), *changes* from $N_0$ to *N* (index c), or stays at *N* (no index), respectively. We also write ${{{\mathcal {X}}}}(0)={{{\mathcal {X}}}}_0$, ${{{\mathcal {X}}}}(t)={{{\mathcal {X}}}}$ for *t>0*, $\theta (0)=\theta _0 = N_0\mu$, $\theta (t)=\theta = N\mu$ for *t>0*, $\varepsilon (0)=\varepsilon _0 = 1/(2N_0)$, and $\varepsilon (t)=\varepsilon = 1/(2N)$ for *t> 0*.

Watterson (1984) obtained approximations of the EAFS from a sample of a large population with *d=1* that undergoes a population size change (86)-(88), whereas Muruyama and Fuerst (1985) obtained numerical approximations of the EAFS for such a model. An important application of the sudden population size change model (86)-(88) are bottlenecks, with $N<N_0$ the size of a smaller population that temporarily lasts for *t* generations. Bottlenecks are a well known cause of loss of genetic variation and increased inbreeding (Allendorf 1986; Frankham et al. 2010). It is also known that a short and severe (small $N/N_0$) bottleneck has a more detrimental effect on the expected number of alleles than a long and diffuse (larger $N/N_0$) bottleneck, whereas the gene diversity is similarly affected by these two bottleneck scenarios (Nei et al. 1975; England et al. 2003). For this reason it is of interest to give explicit, analytical expressions of how the expected number of alleles and the gene diversity change after a population size reduction.

### Example 18

*(Expected number of alleles.)* It follows from (48) that the expected number of alleles, *t* generations after the population change, equals

90 $$\begin{aligned} E[A_n(t)] = \sum _{x\in {{{\mathcal {X}}}}_0} f_x(0)[1-(\boldsymbol{ P}_{\mathrm{c}}\boldsymbol{ P}^{t-1})_{x0}] + 2\theta \sum _{s=1}^t [1-P_{\varepsilon 0}^{t-s}] \end{aligned}$$

for *t>0*, with $P_{\varepsilon 0}^{t-s} = (\boldsymbol{ P}^{t-s})_{\varepsilon 0}$. The first term of (90) is transient and tends to zero as $t\rightarrow \infty$, whereas the second term converges to a new equilibrium value (70) of $E[A_n(t)]$. Of particular interest is the case when $\boldsymbol{ f}(0)$ is chosen according the old equilibrium, before the population change. This corresponds to choosing $\boldsymbol{ f}_{(-0)}(0)$ as in (59), with $\boldsymbol{ P}_0$, $\theta _0 = N_0\mu$ and $\varepsilon _0=1/(2N_0)$ in place of $\boldsymbol{ P}$, *θ*, and *\varepsilon*. When this $\boldsymbol{ f}(0)$ is inserted into (90), the resulting formula

91 $$\begin{aligned} E[A_n(t)] = 2\theta _0 \sum _{s=0}^\infty [1-(\boldsymbol{ P}_0^s\boldsymbol{ P}_{\mathrm{c}}\boldsymbol{ P}^{t-1})_{\varepsilon _0,0}] + 2\theta \sum _{s=1}^t [1-P_{\varepsilon 0}^{t-s}] \end{aligned}$$

for *t>0* reveals how the expected number of alleles transitions from one equilibrium value (70) to another. In Appendix B.3 we use branching process approximations of the number of copies of one allele, for monoecious populations, to motivate that $E[A_n(t)]$ transitions from the old equilibrium $E[A_n(0)]$ to the new equilibrium $E[A_n(\infty )]$ in $O(\mu ^{-1})$ generations (by this we mean at most $C\mu ^{-1}$ generations, for some constant *C* independent of *μ*). This can be explained by the fact that

92 $$\begin{aligned} E[A_n(t)]-E[A_n(\infty )] = 2\theta _0 \sum _{s=0}^\infty [1-(\boldsymbol{ P}_0^s\boldsymbol{ P}_{\mathrm{c}}\boldsymbol{ P}^{t-1})_{\varepsilon _0,0}] - 2\theta \sum _{s=0}^\infty [1-P_{\varepsilon 0}^{t+s}]. \end{aligned}$$

Formula (92) involves non-absorption probabilities $1-(\boldsymbol{ P}_0^s\boldsymbol{ P}_{\mathrm{c}}\boldsymbol{ P}^{t-1})_{\varepsilon _0,0}$ and $1-P_{\varepsilon 0}^{t+s}$ over *t+s* generations, for *s=0,1,2,…*. These non-absorption probabilities tend to zero at a multiplicative rate

$$\begin{aligned} O(\lambda _1^{t+s})=O[(1-\mu )^{t+s}]\approx O[\exp (-(t+s)\mu )] \end{aligned}$$

when *t* is kept fixed and *s* grows, with $\lambda _1$ the second largest eigenvalue of $\boldsymbol{ P}$ (cf. (64)). From this it follows that

93 $$\begin{aligned} \frac{E[A_n(t)]-E[A_n(\infty )]}{E[A_n(0)]-E[A_n(\infty )]} = O[\exp (-t\mu )]. \end{aligned}$$

Note however that Nei and Li (1976) use diffusion approximations to obtain a larger rate of convergence towards the new equilibrium, based on the third largest eigenvalue $\lambda _2$ of *P* (cf. (66)) rather than $\lambda _1$. This larger rate of convergence not only incorporates the mutation rate *μ*, as in (93), but also the effect of genetic drift.

Recall from (11) that allelic diversity $A_D(t)$ quantifies the effect a population change after generation 0 has on the fraction of excess number of alleles of generation *t*. Regarding $\boldsymbol{ f}(0)$ and $A_n(0)$ as fixed, it follows from (10), (11), and (90) that the expected allelic diversity of generation *t*, is

94 $$\begin{aligned} & E[A_D(t)] = \frac{E[A_n(t)]-1}{A_n(0)-1} \nonumber \\ & \quad = \frac{\sum _{x\in {{{\mathcal {X}}}}_0} f_x(0)[1-(\boldsymbol{ P}_{\mathrm{c}}\boldsymbol{ P}^{t-1})_{x0}] + 2\theta \sum _{s=1}^t [1-P_{\varepsilon 0}^{t-s}] - 1}{\sum _{x\in {{{\mathcal {X}}}}_0\setminus \{0\}} f_x(0) - 1}. \end{aligned}$$

The mutation rate *μ* is often ignored when changes of allelic diversity are studied over short time frames. This corresponds to assuming that *θ* is small, and dropping the second sum of the numerator of (94).

### Example 19

*(Expected gene diversity.)* Let us derive a formula for the expected gene diversity of a monoecious population after a population size change. We make use of (54), with census and effective population sizes as in (86) and (87). This yields

95 $$\begin{aligned} \begin{array}{rcl} E[H(t)] & =& e_0 [1-1/(2N_e)]^{t-1} (1-\mu )^{2t} H(0)\\ & +& \mu (2-\mu )(1-1/(2N))\sum _{s=1}^t [(1-1/(2N_e))(1-\mu )^2)]^{t-s}, \end{array} \end{aligned}$$

with

$$\begin{aligned} \begin{array}{rcl} e_0 & =& e(1) = 1-\frac{d}{2N_e}-\frac{(1-d)c_0}{2N_{e0}},\\ c_0 & =& c(0) = \frac{1-1/(2N)}{1-1/(2N_0)}. \end{array} \end{aligned}$$

Note that the first term on the right-hand side of (95) is transient, whereas the second term converges to the new equilibrium value (76) of the expected gene diversity, as $t\rightarrow \infty$. It follows from (66) and (95) that the time required for the transient terms to be negligible, and obtain a new equilibrium for gene diversity, is of the order $O[(1-\lambda _2)^{-1}]$. It accounts for loss of alleles through genetic drift (rate $1/N_e$) and mutations (rate *μ*), and emergence of new alleles through mutations (rate *μ*). This time to reach equilibrium is smaller than the order $O[(1-\lambda _1)^{-1}]=O(\mu ^{-1})$ reported in Nei (1975), which only incorporates mutations. The larger the rate of genetic drift is in comparison to the mutation rate (that is, the smaller $\theta _e = N_e\mu$ is) the larger is the difference between the lengths of the two time intervals to reach equilibrium.

## Grid-Based Approximations of the EAFS

It is currently infeasible to implement the EAFS recursion in (38) from generation *t* to *t+1*, for populations of size larger than 100–1000, since the sizes *2N(t)+1* and *2N(t+1)+1* of the involved sets of allele frequencies (${{{\mathcal {X}}}}(t)$ and ${{{\mathcal {X}}}}(t+1)$) then become too large. For this reason we divide ${{{\mathcal {X}}}}(t)$ into a disjoint and ordered union of *K*(*t*) intervals, with *K*(*t*) much smaller than *2N(t)+1*. It is assumed that intervals with allele frequencies close to 0 or 1 have unit length, whereas intervals of allele frequencies of intermediate size have length *q*, where *q≥ 1* is an odd number. The rationale for this construction is that higher resolution is typically needed for finding the EAFS at low and high allele frequencies. Note that *q=1* corresponds to an exact algorithm ($\hat{{{{\mathcal {X}}}}}(t)={{{\mathcal {X}}}}(t)$), whereas larger values of *q* correspond to a coarser grid of allele frequencies ($|\hat{{{{\mathcal {X}}}}}(t)|<|{{{\mathcal {X}}}}(t)|$).

Let *I* be one of the *K*(*t*) intervals that ${{{\mathcal {X}}}}(t)$ is divided into, and denote the center point of *I* by *a=a(I)*. Then

96 $$\begin{aligned} \hat{{{{\mathcal {X}}}}}(t) = \{a; \, a=a(I) \text{ for } \text{ some } I\subset {{{\mathcal {X}}}}(t)\} \end{aligned}$$

is a sub-grid of ${{{\mathcal {X}}}}(t)$ that consists of the midpoints of all *K*(*t*) intervals that ${{{\mathcal {X}}}}(t)$ is divided into. A grid-based approximation of the EAFS of generation *t* is defined as a row vector

97 $$\begin{aligned} \hat{\boldsymbol{ F}}(t) = ({\hat{F}}_a(t); \, a\in \hat{{{{\mathcal {X}}}}}(t)) \end{aligned}$$

of length $|\hat{{{{\mathcal {X}}}}}(t)|$ on (96), where

98 $$\begin{aligned} {\hat{F}}_a(t) \approx \sum _{x\in I_a} F_x(t) \end{aligned}$$

is the approximate expected allele frequency at $a\in \hat{{{{\mathcal {X}}}}}(t)$ and $I_a\subset {{{\mathcal {X}}}}(t)$ is the interval that surrounds *a*. This gives rise to a grid-based analogoue

99 $$\begin{aligned} \hat{\boldsymbol{ F}}(t+1) = \hat{\boldsymbol{ F}}(t)\hat{\boldsymbol{ P}}(t) + 2\theta (t+1)\hat{\boldsymbol{ \delta }}_{\varepsilon (t+1)}(t+1) \end{aligned}$$

of matrix recursion (38), where

100 $$\begin{aligned} \hat{\boldsymbol{ P}}(t) = ({\hat{P}}_{ab}(t); \, a\in \hat{{{{\mathcal {X}}}}}(t), b\in \hat{{{{\mathcal {X}}}}}(t+1)) \end{aligned}$$

is a transition matrix with elements

101 $$\begin{aligned} {\hat{P}}_{ab}(t) \approx \frac{1}{|I_a|} \sum _{x\in I_a,y\in J_b} P_{xy}(t), \end{aligned}$$

where $J_b\subset {{{\mathcal {X}}}}(t+1)$ is the interval that surrounds $b\in \hat{{{{\mathcal {X}}}}}(t+1)$, whereas

$$\begin{aligned} \hat{\boldsymbol{ \delta }}_a(t+1) = (1(b=a); \, b\in \hat{{{{\mathcal {X}}}}}(t+1)) \end{aligned}$$

defines a row vector of length $|\hat{{{{\mathcal {X}}}}}(t+1)|$ for any $a\in \hat{{{{\mathcal {X}}}}}(t+1)$. Algorithms for computing the grid-based matrix recursion (99) of the EAFS are defined in Appendix C, separately for monoecious and dioecious populations. Making use of one of these algorithms, it is possible to approximate the expected value (42) of a summary statistic of the EAFS, as

102 $$\begin{aligned} {\hat{E}}[S_g(t)] = \sum _{a\in \hat{{{{\mathcal {X}}}}}(t)} {\hat{F}}_a(t)g(a) = \hat{\boldsymbol{ F}}(t)\hat{\boldsymbol{ g}}(t), \end{aligned}$$

with $\hat{\boldsymbol{ g}}(t) = (g(a); a\in \hat{{{{\mathcal {X}}}}}(t))^\prime$ a column vector of length $|\hat{{{{\mathcal {X}}}}}(t)|$, with values of the function *g* at the allele frequencies of $\hat{{{{\mathcal {X}}}}}(t)$.

## Numerical Illustrations

In this section we illustrate how a number of summary statistics of the expected allele frequency spectrum changes over time. In Section 10.1 we consider a small population and use the exact recursion (38) of the EAFS, whereas in Section 10.2 we study a somewhat larger population and use the approximate recursion (99).

### Small Populations

**Fig. 1:**
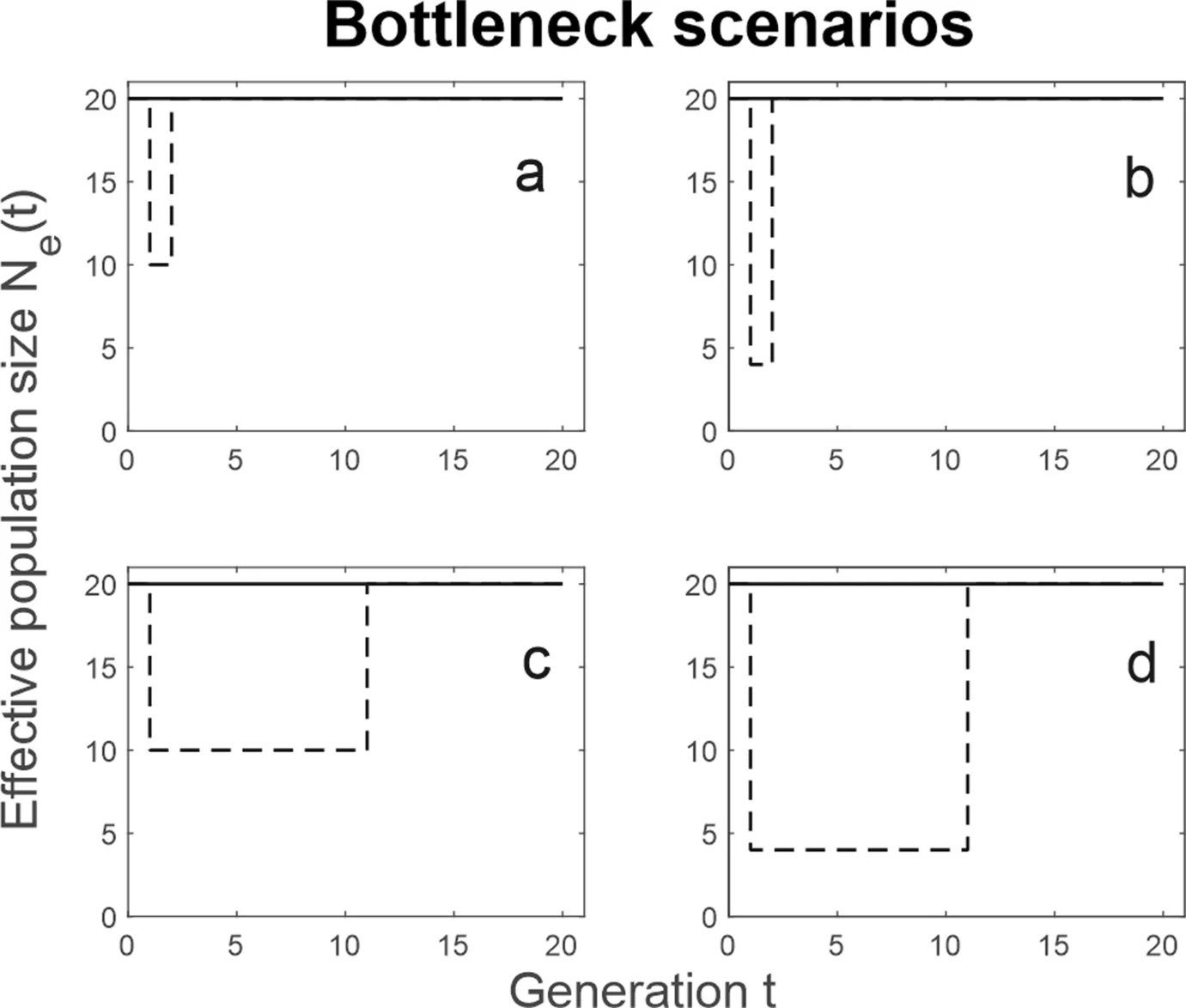
Population size variations over 20 generations (*t=0,1,… ,20*) for the four subplots of Figs. 2–5; two for a monoecious population (Fig. 2 and 3) and two for a dioecious population (Fig. 4 and 5). The census size *N(t)=20* is constant (solid lines), whereas the effective population size $N_e(t)$ varies according to a bottleneck (dashed lines). The bottleneck lasts for $\tau _2-\tau _1+1$ generations ($t=\tau _1,\ldots ,\tau _2$) with effective size $N_e(t)=20$ before and after the bottleneck. The bottleneck is short (length 1, $\tau _1=\tau _2=1$) for the upper subplots (a,b) and long (length 10, $\tau _1=1$, $\tau _2=10$) for the lower subplots (c,d). The bottleneck is shallow ($N_e(t)=10$, with $N_{em}=N_{ef}(t)=5$ for the dioecious population) for the left subplots (a,c) and deep for the right subplots (b,d). The value of the effective size for the deep bottleneck is $N_e(t)=4$ for the monoecious population and $N_e(t)=3.8$ for the dioecious population ($N_{em}(t)=1$, $N_{ef}(t)=19$)

**Fig. 2:**
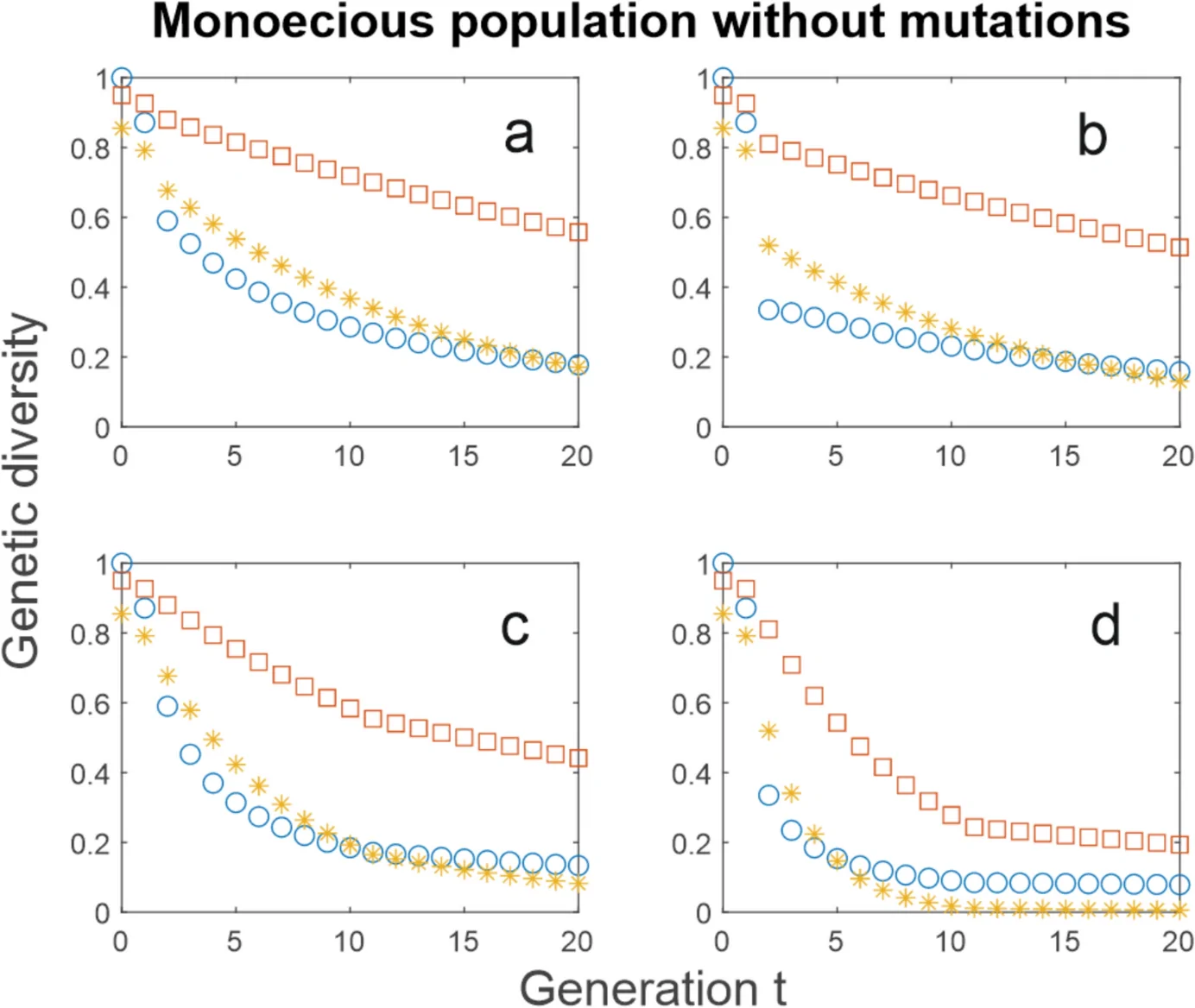
Plots of three measures of genetic variation; the standardized expected number of alleles $E[A_n(t)]/A_n(0)$ (circles), expected gene diversity *E*[*H*(*t*)] (squares), and expected triplet gene diversity $E[H_3(t)]$ (stars) over 20 generations *t=0,1,… ,20*, for a monoecious population of constant census size *N(t)=20* without mutations (*μ =0*). The population starts with $A_n(0)=20$ alleles with 2 copies each (frequency *x=2/40=0.05*) and then experiences a bottleneck. The four subplots a-d correspond to the four bottleneck scenarios of Fig. 1

**Fig. 3:**
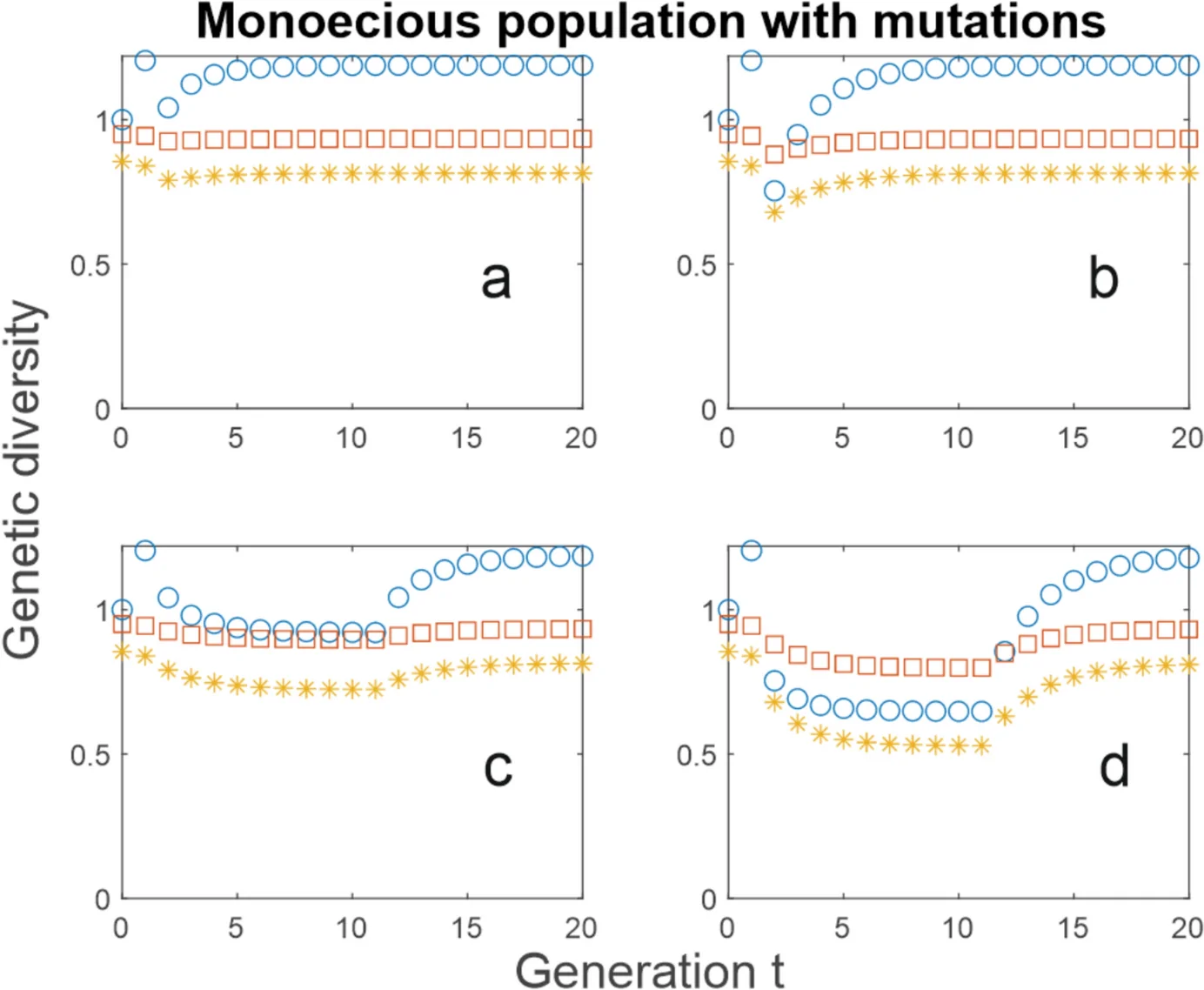
Plots of three measures of genetic variation; the standardized expected number of alleles $E[A_n(t)]/A_n(0)$ (circles), expected gene diversity *E*[*H*(*t*)] (squares), and expected triplet gene diversity $E[H_3(t)]$ (stars) over 20 generations *t=0,1,… ,20*, for a monoecious population of constant census size *N(t)=20* with mutations. The initial allele frequency distribution at *t=0* and the different bottleneck scenarios of the four subplots **a**–**d** are the same as in Fig. 2, as illustrated in Fig. 1. A large value of the mutation rate (*μ =0.2*) was chosen to illustrate how equilibrium is attained during the long bottleneck (right subplots **b**,**d**) and after the bottleneck (all subplots a-d)

**Fig. 4:**
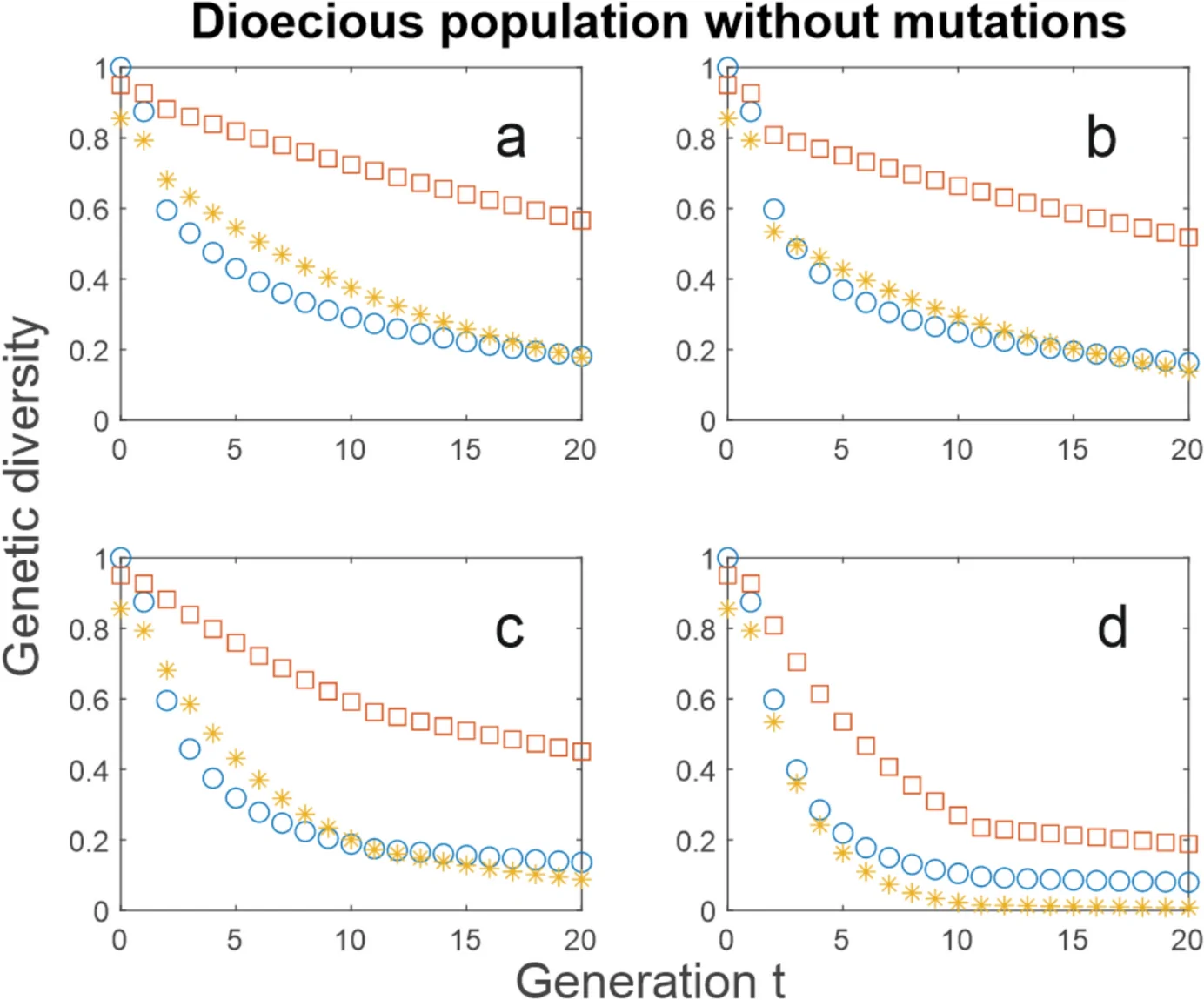
Plots of three measures of genetic variation; the standardized expected number of alleles $E[A_n(t)]/A_n(0)$ (circles), expected gene diversity *E*[*H*(*t*)] (squares), and expected triplet gene diversity $E[H_3(t)]$ (stars) over 20 generations *t=0,1,… ,20*, for a dioecious population of constant census size *N(t)=20* without mutations (*μ =0*). The population starts with $A_n(0)=20$ alleles with 2 copies each (frequency *x=2/40=0.05*) and then experiences a bottleneck. The four subplots a-d correspond to the four bottleneck scenarios of Fig. 1

**Fig. 5:**
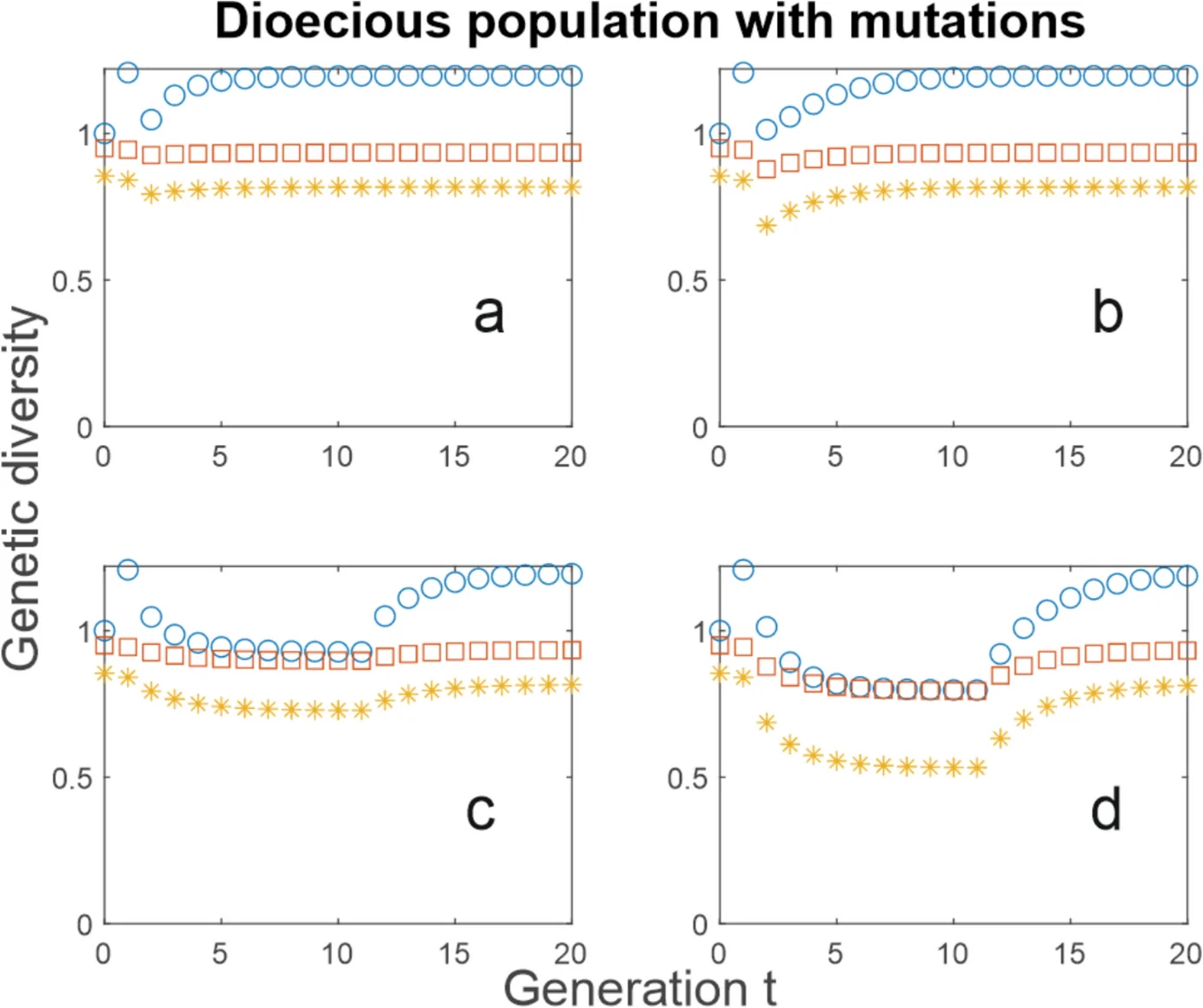
Plots of three measures of genetic variation; the standardized expected number of alleles $E[A_n(t)]/A_n(0)$ (circles), expected gene diversity *E*[*H*(*t*)] (squares), and expected triplet gene diversity $E[H_3(t)]$ (stars) over 20 generations *t=0,1,… ,20*, for a dioecious population of constant census size *N(t)=20* with mutations. The initial allele frequency distribution at *t=0* and the different bottleneck scenarios of the four subplots a-d are the same as in Fig. 4, as illustrated in Fig. 1. A large value of the mutation rate (*μ =0.2*) was chosen to illustrate how equilibrium is attained during the long bottleneck (right subplots b,d) and after the bottleneck (all subplots a-d)

Consider a small monoecious or dioecious population of constant size *N(t)=20* over 20 generations, for which mutations are either absent or present. The population experiences a bottleneck where the effective size $N_e(t)$ declines. There are four types of bottlenecks a-d; a long or short one, and a shallow or deep one (see Figure 1). In Figures 2-5 we illustrate how three different measures of genetic variation vary over time. These measures of genetic variation are the expected gene diversity *E*[*H*(*t*)], the expected triplet gene diversity $E[H_3(t)]$ and a scaled version

103 $$\begin{aligned} \frac{E[A_n(t)]}{A_n(0)} = \frac{1}{A_n(0)} + \left( 1-\frac{1}{A_n(0)}\right) E[A_D(t)] \end{aligned}$$

of the expected number $E[A_n(t)]$ of alleles, which is closely related to the expected allelic diversity $E[A_D(t)]$ (cf. Equation (11)).

It can be seen from these figures that $E[A_n(t)]$ is affected more strongly by any type of bottleneck than *E*[*H*(*t*)], with $E[H_3(t)]$ in between. The effective size variation of each type of bottleneck is essentially the same for the monoecious and dioecious populations. And since changes of *E*[*H*(*t*)] and $E[H_3(t)]$ mainly depend on effective size, this explains why these two measures of genetic variation have a similar behaviour for the monoecious and dioecious populations, for each type of bottleneck. On the other hand, since changes of $E[A_n(t)]$ is affected not only by effective size, but also by census size and total number of breeders ($N_e(t)$ breeders for a monoecious population and $N_{em}(t)+N_{ef}(t)$ breeders for a dioecious population), $E[A_n(t)]$ behaves differently from *E*[*H*(*t*)] and $E[H_3(t)]$ in Figures 2-5.

For the two shallow bottlenecks (subplots a and c of Fig. 1–5), not only the effective size but also the number of breeders is the same for the monoecious and dioecious populations ($N_e(t)=N_{em}(t)+N_{ef}(t)$ in (2)), due to an even sex ratio of the latter, among those that reproduce. This implies that $E[A_n(t)]$ drops by the same amount for the monoecious and dioecious populations, for these two bottlenecks. On the other hand, for the two deep bottlenecks (subplots b and d of Fig. 1–5) the number of breeders of the monoecious population is smaller than for the dioecious population ($N_e(t)<N_{em}(t)+N_{ef}(t)$ in (2)), due to a skewed sex ratio of the latter. As a consequence, $E[A_n(t)]$ drops less for the dioecious population compared to the monoecious population, for these two types of bottlenecks.

The fact that not only *N*(*t*) and $N_e(t)$, but also the number of breeders, affect some quantities of genetic variation, is reinforced in Table 3. In this table the expected allele frequency spectrum (EAFS) and some of its summary statistics are compared at genetic drift-mutation equilibrium, for three pairs of monoecious and dioecious populations, each population pair having the same census size, and the same, or almost the same, effective size. For the first pair (columns 2–3) and the second pair (columns 5–6) the number of breeders of the monoecious and dioecious populations are also the same, and consequently their EAFSs are very similar. In contrast, for the third pair (columns 8–9) the number of breeders of the monecious population is smaller than for the dioecious population, due to a skewed sex ratio of the latter. This implies that the EAFSs of these two populations differ. In particular, the expected number $F_{1/40}(\infty )$ of alleles with only one gene copy, as well as the expected total number $E[A_n(t)]$ of alleles, is larger for the dioecious population. It can also be seen that the asymptotic approximations of the equilibrium EAFSs (columns 4, 7, and 10) underestimate the expected number of alleles with only one gene copy, as well as the expected total number of alleles, when $N_e$ is small, particularly so for a dioecious population with a skewed sex ratio. This is due to the fact that the asympotic formula only includes census and effective sizes, not the number of breeders.

**Table 3:** Expected allele frequency spectra $\{F_x(\infty );\, x=1/(2N),\ldots , (2N-1)/2N,1\}$ of existing (non-lost) alleles for monoecious and dioecious diploid populations with constant census size *N(t)=N=20*, constant effective size $N_e(t)=N_e$ and mutation rate *μ =0.2*. Consequently there are *2N=40* genes in each generation, and an allele with *n* copies has a frequency of *x=n/(2N)*. All populations are in genetic drift-mutation equilibrium (cf. (58))

Allele frequency	*N=20*,$N_e=20$			*N=20*,$N_e=10$			*N=20*,$N_e=3.8$or 4		
*x=n/(2N)*	Mono	Dio	Appr	Mono	Dio	Appr	Mono	Dio	Appr
0.025	15.6408	15.7861	14.4241	10.8799	10.9190	8.0345	8.2168	11.8913	3.4369
0.050	4.2000	4.2683	4.4645	2.6488	2.7253	3.0895	0.4731	1.1584	1.5054
0.075	1.9700	1.9745	1.9613	1.7925	1.8331	1.6842	0.6768	0.4253	0.9343
0.100	0.9430	0.9324	0.9690	1.0868	1.0950	1.0372	0.7212	0.2626	0.6568
0.125	0.4780	0.4673	0.5065	0.6668	0.6646	0.6795	0.6240	0.2191	0.4928
0.150	0.2513	0.2428	0.2728	0.4296	0.4256	0.4616	0.4750	0.2132	0.3848
0.175	0.1344	0.1283	0.1493	0.2866	0.2822	0.3208	0.3465	0.2227	0.3087
0.200	0.0725	0.0683	0.0823	0.1944	0.1899	0.2262	0.2597	0.2291	0.2523
0.225	0.0392	0.0365	0.0454	0.1327	0.1286	0.1609	0.2047	0.2204	0.2091
0.250	0.0212	0.0195	0.0250	0.0909	0.0873	0.1151	0.1669	0.1960	0.1750
0.275	0.0114	0.0104	0.0137	0.0623	0.0594	0.0825	0.1379	0.1639	0.1477
0.300	0.0061	0.0055	0.0074	0.0427	0.0403	0.0592	0.1144	0.1325	0.1253
0.325	0.0032	0.0029	0.0040	0.0292	0.0273	0.0423	0.0951	0.1060	0.1067
0.350	0.0017	0.0015	0.0021	0.0199	0.0184	0.0302	0.0792	0.0850	0.0912
0.375	0.0009	0.0008	0.0011	0.0135	0.0124	0.0214	0.0659	0.0687	0.0781
0.400	0.0005	0.0004	0.0006	0.0091	0.0083	0.0151	0.0548	0.0562	0.0669
0.425	0.0002	0.0002	0.0003	0.0060	0.0055	0.0105	0.0454	0.0471	0.0573
0.450	0.0001	0.0001	0.0001	0.0040	0.0036	0.0073	0.0375	0.0404	0.0491
0.475	0.0001	0.0000	0.0001	0.0026	0.0023	0.0050	0.0309	0.0351	0.0420
0.500	0.0000	0.0000	0.0000	0.0017	0.0015	0.0034	0.0253	0.0306	0.0358
0.525	0.0000	0.0000	0.0000	0.0011	0.0009	0.0022	0.0207	0.0265	0.0305
0.550	0.0000	0.0000	0.0000	0.0007	0.0006	0.0015	0.0168	0.0226	0.0258
0.575	0.0000	0.0000	0.0000	0.0004	0.0003	0.0009	0.0135	0.0191	0.0218
0.600	0.0000	0.0000	0.0000	0.0002	0.0002	0.0006	0.0108	0.0158	0.0183
0.625	0.0000	0.0000	0.0000	0.0001	0.0001	0.0004	0.0086	0.0128	0.0152
0.650	0.0000	0.0000	0.0000	0.0001	0.0001	0.0002	0.0067	0.0100	0.0126
0.675	0.0000	0.0000	0.0000	0.0000	0.0000	0.0001	0.0052	0.0076	0.0103
0.700	0.0000	0.0000	0.0000	0.0000	0.0000	0.0001	0.0040	0.0054	0.0083
0.725	0.0000	0.0000	0.0000	0.0000	0.0000	0.0000	0.0030	0.0036	0.0066
0.750	0.0000	0.0000	0.0000	0.0000	0.0000	0.0000	0.0022	0.0023	0.0052
0.775	0.0000	0.0000	0.0000	0.0000	0.0000	0.0000	0.0015	0.0013	0.0040
0.800	0.0000	0.0000	0.0000	0.0000	0.0000	0.0000	0.0011	0.0007	0.0030
0.825	0.0000	0.0000	0.0000	0.0000	0.0000	0.0000	0.0007	0.0003	0.0022
0.850	0.0000	0.0000	0.0000	0.0000	0.0000	0.0000	0.0004	0.0001	0.0015
0.875	0.0000	0.0000	0.0000	0.0000	0.0000	0.0000	0.0002	0.0000	0.0010
0.900	0.0000	0.0000	0.0000	0.0000	0.0000	0.0000	0.0001	0.0000	0.0006
0.925	0.0000	0.0000	0.0000	0.0000	0.0000	0.0000	0.0000	0.0000	0.0003
0.950	0.0000	0.0000	0.0000	0.0000	0.0000	0.0000	0.0000	0.0000	0.0001
0.975	0.0000	0.0000	0.0000	0.0000	0.0000	0.0000	0.0000	0.0000	0.0000
1.000	0.0000	0.0000	0.0000	0.0000	0.0000	0.0000	0.0000	0.0000	0.0000
$E[A_n(\infty )]$	23.7747	23.9458	22.9297	18.4027	18.5317	16.0924	12.9464	15.9317	9.3236
*E[H(∞ )]*	0.9335	0.9345	0.9306	0.8954	0.8973	0.8797	0.7977	0.7940	0.7546
$E[H_3(\infty )]$	0.8147	0.8173	0.8072	0.7233	0.7275	0.6872	0.5286	0.5324	0.4524

The number of breeders of the monoecious population is $N_e=20$ (left), $N_e=10$ (middle) and $N_e=4$ (right). The number of male and female breeders of the dioecious population, and the effective size, is either $N_{em}=N_{ef}=10, N_e=20$ (left), $N_{em}=N_{ef}=5, N_e=10$ (middle) and $N_{em}=1,N_{ef}=19, N_e=3.8$ (right), with $N_e$ computed as in (2). Note the similarity between the second/third as well as between the fifth/sixth columns. This is because the number of breeders of the monoecious and dioecious populations is the same, when the latter has an even sex ratio. On the other hand, there is a large difference between the eighth/ninth columns, because the number of breeders of the monoecious and dioecious populations are not the same, when the latter has a skewed sex ratio. For comparison, an asymptotic approximation $F_x^{\mathrm{appr}}(\infty )=\int _{I_x} 4\theta _e (1-y)^{4\theta _e-1}y^{-1}dy$ of the EAFS at drift-mutation equilibrium based on (67) (with $I_x$ an interval surrounding *x*) is provided as well for $N_e=20$ (left), $N_e=10$ (middle) and $N_e=4$ (right). This approximate EAFS is normalized to have a total allele frequency of 1, cf. (C.23)

### Moderately Large Populations

**Fig. 6:**
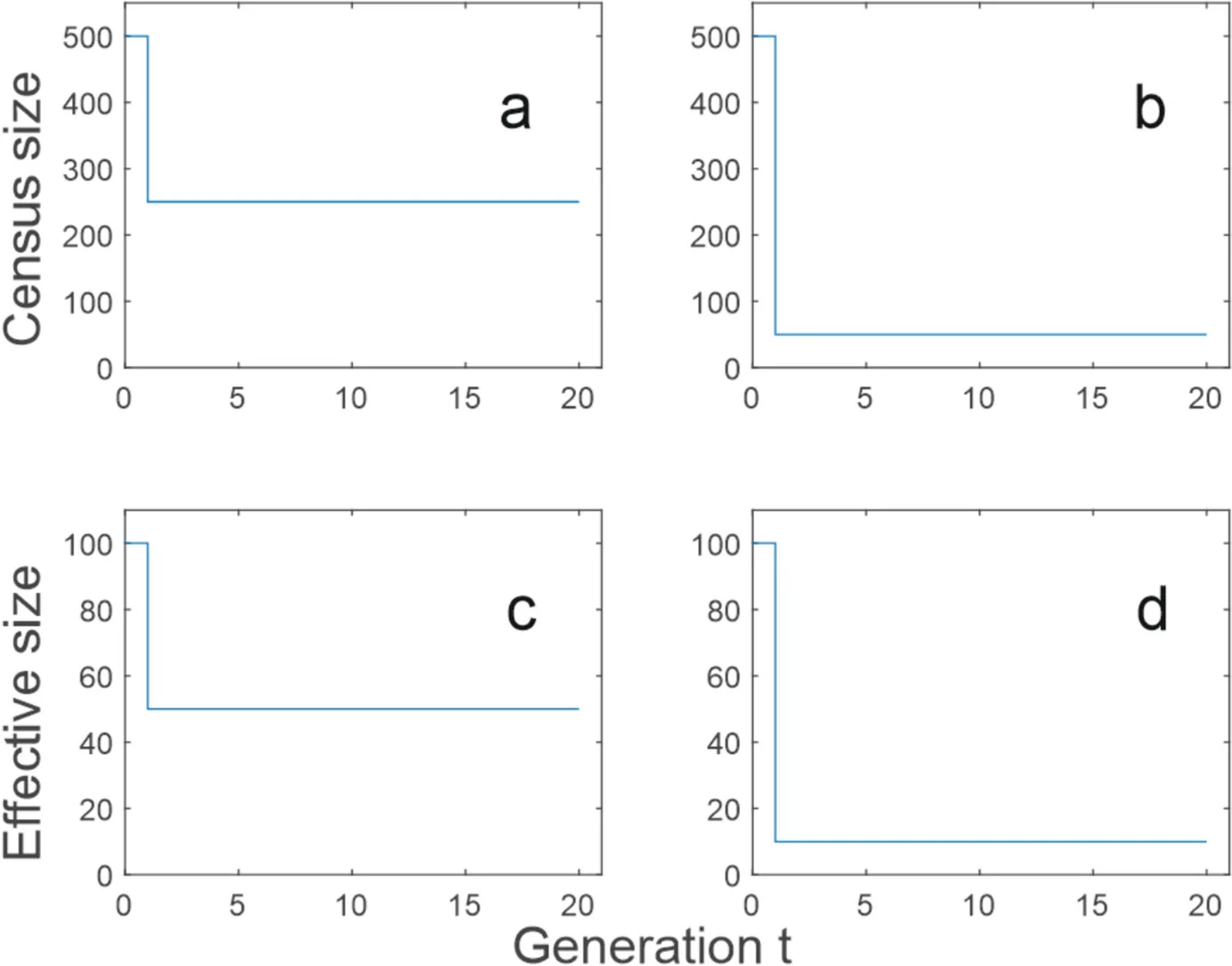
Two scenarios of population size decrease for a monecious population over 20 generations (*t=0,1,… ,20*). For both scenarios the population starts with census size *N(0)=500* and effective size $N_e(0)=100$ in generation 0. In the first scenario (left subplots a,c) the census and effective sizes reduce to 50% after one generation, whereas in the second scenario (right subplots b,d) the census and effective sizes reduce to 10% after one generation. These two scenarios correspond to the left and right subplots of Fig. 7

**Fig. 7:**
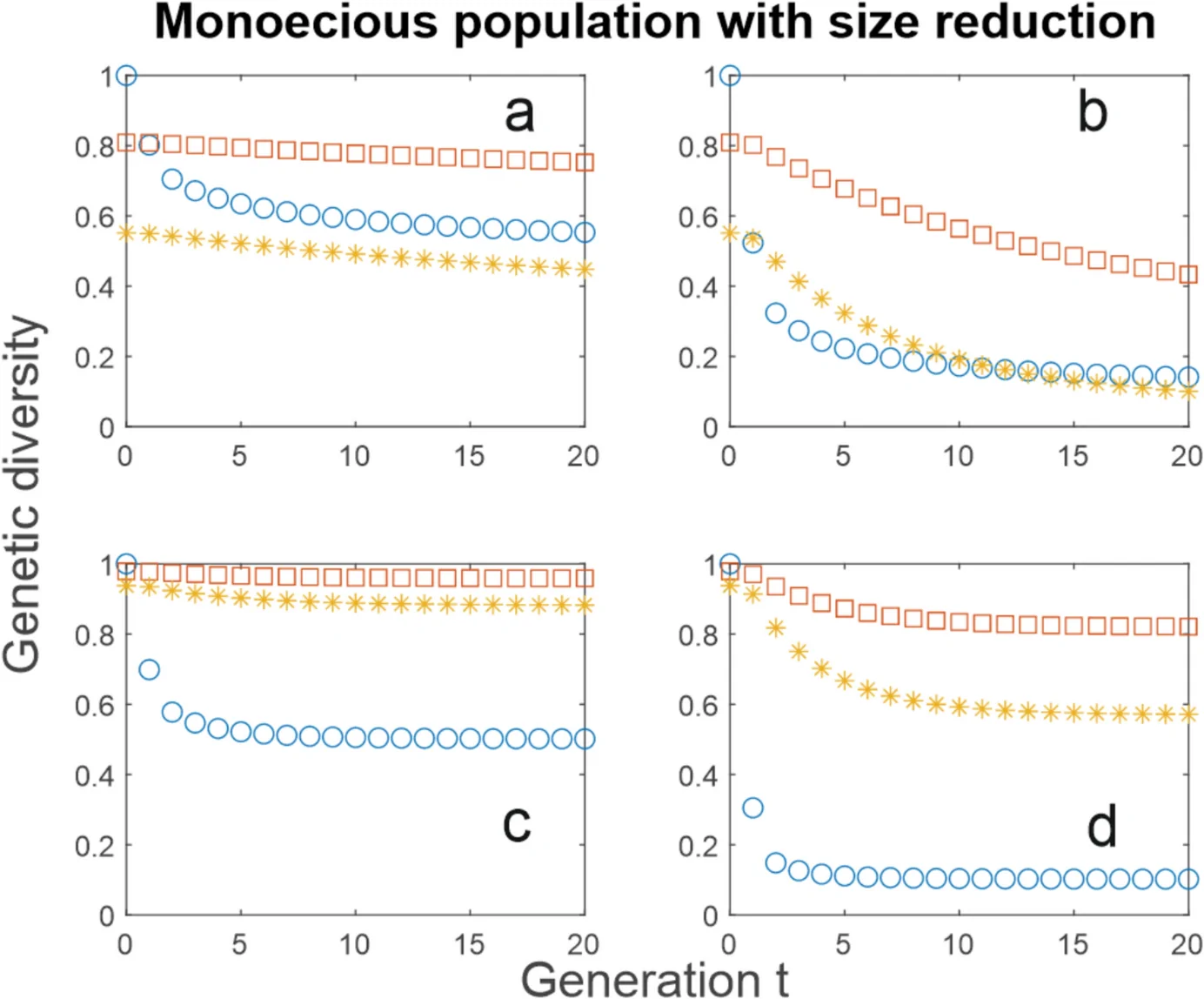
Plots of three measures of genetic variation; the standardized expected number of alleles ${\hat{E}}[A_n(t)]/{\hat{E}}[A_n(0)]$ (circles), the expected gene diversity ${\hat{E}}[H(t)]$ (squares), and the expected triplet gene diversity ${\hat{E}}[H_3(t)]$ (stars) over 20 generations *t=0,1,… ,20*, for a monoecious population that undergoes a sudden population size decline after one generation, where the census and effective size either reduce to 50% (left subplots a,c of this figure and of Figure 6) or to 10% (right subplots b,d of this figure and of Figure 6). The mutation rate is either *μ =0.01* (upper subplots a,b) or *μ =0.1* (lower subplots c,d). The expected number of alleles in equilibrium, before the population size decline, is ${\hat{E}}[A_n(0)]=27.5$ for *μ =0.01* and ${\hat{E}}[A_n(0)]=180$ for *μ =0.1*

In this section we study how the genetic variability of a moderately large, monoecious population with mutations decreases over 20 generations, after a sudden drop of the population size (see Fig. 6 and 7). We consider three different measures of genetic variation; the standardized expected number of alleles ${\hat{E}}[A_n(t)]/{\hat{E}}[A_n(0)]$ analogously to (103), the expected gene diversity ${\hat{E}}[H(t)]$ and the expected triplet gene diversity ${\hat{E}}[H_3(t)]$. Initially at time *t=0* the population has census size $N(0)=N_0=500$, effective size $N_e(0)=N_{e0}=100$, and equilibrium (59) between genetic drift and mutations. The population then undergoes a sudden change according to (86)–(87), so that the census size drops to $N(t)=N<N_0$ and the effective size drops to $N_e(t)=N_e<N_{e0}$ for *t>0*. This population size decline is either moderate ($N/N_0=N_e/N_{e0}=0.5$) or large ($N/N_0=N_e/N_{e0}=0.1$), as illustrated in subplots a,c and b,d of Fig. 6, respectively.

Because of the large population size, the approximate recursive algorithm (99) for the EAFS is used, and the three expected summary statistics of the EAFS are computed from (102). The EAFS recursion (99) is based on an approximate grid for which the interior allele frequency intervals have length *q=3* whenever the census size is 500 or 250, whereas a full grid of allele frequencies, with *q=1*, is used for smaller census sizes and all effective sizes. The transition probabilities in (100) are computed exactly for allele frequencies close to 0 or 1, and based on the normal approximations in (C.14) for intermediate allele frequencies. We use normal approximations since the exact transition probabilities require binomial coefficients that are too large to compute for intermediate allele frequencies and large populations. The EAFS of each generation is normalized to a total allele frequency of 1, according to (C.23). See Appendix C for more details.

Figure 7 has four subplots a-d, corresponding to the moderate or large population size declines of Fig. 6, and two different mutation rates. Note in particular that the expected number of alleles changes much more abruptly after the population size decline, than the expected gene diversity and the expected triplet gene diversity. This is also true for the smaller mutation rate *μ =0.01*, in spite of the fact that it takes a long time ($O(\mu ^{-1})$ generations, cf. (93)) before a new equilibrium between genetic drift and mutation is attained.

## Discussion

### Summary of the article

In this paper we present a general framework for the dynamics of the expected locus-wise allele frequency spectrum (EAFS) at a polymorphic portion of DNA, for a spatially homogeneous monoecious or dioecious population. More specifically, we develop four novelties. First, we study the dynamics of several functionals of the EAFS that represent various measures of genetic variability, within a unified framework (Sections 3 and 6). This includes the expected number of alleles, the expected gene diversity, the expected triplet gene diversity, as well as the expected Hill numbers. Second, whereas previous work on allele frequency spectra mainly used diffusion approximations for large populations, we focus on exact matrix analytical methods to evaluate how the EAFS, and its functionals, change over time (Sections 4-5 and Appendix A). Since these exact methods do not rely on asymptotic approximations, they are reliable also for very small populations. Third, we consider monoecious as well as dioecious populations with separate census and effective sizes (Section 2). This is important, since most summary statistics of the EAFS are functions not only of the effective size, but also of the census size. Fourth, in order to make our methodology feasible also for large populations we use numerical approximations for the dynamics of the EAFS, based on a grid of allele frequencies of smaller size than for the exact EAFS (Section 9 and Appendix C). Our approach is illustrated for populations that reach an equilibrium between genetic drift and mutations (Section 7 and Appendix B), experience a sudden population change (Section 8), and numerically for small and moderately large populations (Section 10).

An important feature of our model is to divide the reproduction cycle between generations *t* and *t+1* into two time-periods, where $N_e(t)$ breeding individuals are chosen at first, and then *N(t+1)* individuals are formed for generation *t+1*. These *N(t+1)* individuals are also candidate breeders for the next reproduction cycle between generations *t+1* and *t+2*. This type of reproduction cycle makes it possible to have different census and effective sizes *N*(*t*) and $N_e(t)$, a feature that is particularly useful when studying how diversity measures such as the expected number of alleles $E[A_n(t)]$ vary over time. In contrast, the classical Wright-Fisher (WF) model only has one time-period per generation, with no distinction between census and effective sizes. This makes it difficult to use the WF model for studying $E[A_n(t)]$ and other measures of genetic variation that include the census as well as the effective size. On the other hand, the dynamics of the expected gene diversity *E*[*H*(*t*)] is mainly determined by effective size, at least to a first approximation. This can be seen from the explicit formula for *E*[*H*(*t*)] in Equation (54), where historical census sizes have a small impact on *E*[*H*(*t*)] unless the census size is very small, whereas effective sizes have a much stronger, cumulative, effect on *E*[*H*(*t*)]. For this reason the expected gene diversity can be studied with good accuracy for all but very small populations, using the simpler WF model.

### Possible extensions

#### Computational Developments

The computational methods presented in this article can be extended in several ways. We computed the EAFS exactly for small populations (Section 10.1) and approximately for moderately large populations (Section 10.2). Computation of the EAFS is more challenging for very large populations, since i) we expect the quality of the approximate EAFS algorithm to decrease with population size, and ii) it is difficult to study equilibrium between genetic drift and mutations, due to the very long burn-in times to reach such equilibria. In order to check the quality of the analytical approximations of the EAFS for very large populations, these analytical EAFS must be compared with simulation-based estimates of the EAFS, obtained by taking the average of a large number of simulated AFS. It is also of interest to make use of modern computational methods to further improve the grid-based algorithm, both in terms of accuracy and speed.

Computational refinements are also needed to analyze the dynamics of the distribution of the AFS, not only how the expected AFS changes over time. While it is challenging to obtain explicit analytical expressions for the distribution of the AFS, simulations can be used to approximate this distribution. Fast population genetic simulators, such as the forwards-in-time algorithm SLiM 3 (Haller and Messer 2019), or the backward-in-time algorithm msprime (Baumdicker et al. 2022), are both applicable to genomwide simulations. These simulators are therefore well suited for approximating the distribution of the genomewide AFS, whereas less complicated simulators such as EASYPOP (Balloux 2001) might be sufficient to approximate the distribution of the locus-specific AFS in some situations.

#### Model Developments

The models of Section 2 can also be generalized in various ways. The reproduction cycles of these models do not take diploidy into account, and this is no severe limitation when studying measures of genetic variation that are functions of the AFS. However, for populations not in Hardy-Weinberg (HW) equilibrium, it is important to consider measures of genetic variation, such as inbreeding, that more explicitly incorporate genotypes. This includes heterozygosity (the probability of a genotype having different alleles) and the fixation index $F_{IS}$ (Wright 1943; Nei 1977; Hössjer et al. 2015). Since these measures are functions of genotype frequencies rather than allele frequencies, it is of interest to study the genetoype frequency spectrum (GFS) for populations that are not in HW equilibrium.

Another diploid extension of the dioecious model of Section 2.2 is to include the effect of recombination. In this article we analyzed the AFS of a polymorphic locus that is large enough to be polymorphic, with an infinite alleles model for mutations, but small enough to neglect the effect of intralocus recombinations. If intralocus recombinations are to be added, it is more natural to study the variation of genotype frequencies over time, where each genotype consists of two haplotypes, one inherited from the father and one from the mother.

The models of Section 2 can also be extended to incorporate subdivided populations. The dynamics of genetic variability in such populations includes studying gene diversities in the total population and in subpopulations, as well as measures of subpopulation differentiation such as $G_{ST}$ (Nei 1977; Chakraborty 1993; Nagylaki 1998). Other measures of genetic variation focus on rare alleles. For instance, Barton and Slatkin (1986) and Caballero and Garcia-Dorado (2013) analyzed the distribution of rare alleles and the expected number of alleles of an island model in equilibrium. Hill et al. (2023) used simulations to conclude that the expected number of alleles and gene diversity change in different ways for metapopulations whose subpopulations change periodically between being isolated and connected through migration. Pinto et al. (2024) also used simulations to find out that the effect of population decline can be severe for a metapopulation that experiences fragmentation into smaller subpopulations, although this effect is dependent on which measure of genetic variation is used. A general approach for analyzing the dynamics of various measures of genetic variability of subdivided populations, whose subpopulations are in HW equilibriium, could be a) to view these measures as different summary statistics of the expected multipopulation allele frequency spectrum (EMAFS) of a polymorphic loci, and b) to study how the EMAFS vary over time when for instance the census and/or effective sizes of the subpopulations change their values. This is analogous to studying the dynamics of the EMAFS of genomewide collections of biallelic markers (cf. e.g. Gutenkunst et al. (2009); Gravel et al. (2011); Seehausen et al. (2014); Schmutz et al. (2014) and Jouganous et al. 2017)).

It would also be of interest to extend our selectively neutral models of Section 2 to incorporate various forms of natural selection. For instance, Evans et al. (2007) and Kaj and Mugal (2016) incorporated selection parameters in their models for the dynamics of the genomewide AFS. The genomewide AFS has also been used to define various tests of selection (Zeng et al. 2006). Moreover, it is well known that negative selection against alleles as well as transmission bias (in particular biased gene conversion) affect the genomewide AFS (Pouyet et al. 2018). However, it is likely that such selection mechanisms have more impact on the long term rather than the short term dynamics of the locus-wise and genomewide AFS, in particular for diploid models when harmful recessive mutations accumulate.

### Conservation Biology Applications

Various measures of genetic variation are used in biodiversity conservation to assess and monitor population viability and adaptive potential when the environment changes (Andersson et al. 2022; Hoban et al. 2022). Effective size, for instance, is highly relevant for international policy for biodiversity and its implementation. It is now a Headline indicator within the UN Convention on Biological Diversity’s Global Biodiversity Framework and its associated monitoring framework (Mastretta-Yanes and da Silva et al. 2024). But other summary statistics of the AFS are also of interest for conservation. It is well known, for instance, that the number of alleles at a locus is important for quantifying disease resistance and long time fitness (Allendorf 1986; Fuerst and Maruyama 1986; Allendorf et al. 2024). For these reasons, the theoretical approach for the AFS elaborated here is a relevant tool for conservation of genetic variability.

An important aspect of conservation is the effects of bottlenecks on the number of alleles. It is well known that both the census size *N* and the effective size $N_e$ are affected by a bottleneck, but the dynamics of allelic retention under non-equilibrium conditions has been difficult to model previously. Indeed, the present research was initiated to address a question directly relevant for conservation management: what is the evolutionary trajectory of allele loss from the original equilibrium to the next, when a large population (expected to harbor very many alleles at equilibrium) experiences a population size reduction (but still remains large)? This is a question relevant to e.g. harvested fish populations where collapsed fisheries may occur when population sizes go from say 25 billion to 5 billion due to overharvest. Under equilibrium conditions such a reduction is expected to result in a loss of 75% of the alleles while gene diversity is not affected (Ryman et al. 1995; Allendorf et al. 2024). What is unclear is the trajectory of this loss over the generations from the original equilibrium to the new equilibrium. The present theoretical development provides a basis for addressing such questions and many others of relevance for conservation of genetic variability of small, moderately large and large populations. We intend to make use of the insights from the present work to address genetic effects of bottlenecking marine fish populations such as that of the herring of the Baltic Sea. In this context we intend to apply the approximate analytic EAFS algorithm of Section 9 to large populations, and compare the accuracy of the approximation with simulations.

The results of this article will also allow exploring if currently applied indicators to monitor contemporary trends in genetic variation (Laikre et al. 2020; Andersson et al. 2022; Hoban et al. 2023; Mastretta-Yanes and da Silva et al. 2024) need to be modified with respect to other summary statistics of the AFS, such as allelic diversity.

## Data Availability

No datasets were generated or analysed during the current study.

## A Frequency Dynamics for a Single Allele

In this appendix we describe in more detail the Markov process $\{X(t);\, t\ge 0\}$ for the frequency fluctuations of a single allele *a*, that was introduced in Section 4. The monoecious and dioecious populations of Sections 2.1 and 2.2 are treated separately in Appendices A.1 and A.2, respectively.

### A.1 Monoecious Population

It is helpful to introduce *Z*(*t*), the fraction of allele *a* in the breeding pool that is formed in Step 1 of the reproduction cycle of Section 2.1 between generations *t* and *t+1*. Suppose *X(t)=x*. Since $2N_e(t)$ gene copies are drawn randomly without replacement, we find that the number $2N_e(t)Z(t)$ of these gene copies with allele *a*, has a hypergeometric distribution

A.1 $$\begin{aligned} 2N_e(t)Z(t)|X(t)=x \sim \mathrm{Hyp}(2N(t);2N_e(t),x). \end{aligned}$$

Moreover, if *Z(t)=z*, it follows from Steps 2 and 3 of the same reproduction cycle that *2N(t+1)X(t+1)* is the number of the *2N(t+1)* gene copies, drawn with replacement from the breeding pool, that have allele *a* in Step 2 and then do not mutate in Step 3. Since the probability of drawing an *a* allele that does not mutate is *(1-μ )z*, it follows that

A.2 $$\begin{aligned} 2N(t+1)X(t+1) | Z(t)=z \sim \mathrm{Bin}(2N(t+1);(1-\mu )z) \end{aligned}$$

has a binomial distribution. From (A.1) and (A.2) we deduce that the transition probabilities (28) of *X(· )* between generations *t* and *t+1* can be written as a sum

A.3 $$\begin{aligned} P_{xy}(t) = \sum _{z\in {{{\mathcal {Z}}}}(t)} P_{1xz}(t) P_{23,zy}(t), \end{aligned}$$

where

A.4 $$\begin{aligned} P_{1xz}(t) = P(Z(t)=z|X(t)=x) = \frac{{2N(t)x\atopwithdelims ()2N_e(t)z}\cdot {2N(t)(1-x)\atopwithdelims ()2N_e(t)(1-z)}}{{2N(t)\atopwithdelims ()2N_e(t)}} \end{aligned}$$

refers to a transition probability in Step 1 of the reproduction cycle of Section 2.1, whereas

A.5 $$\begin{aligned} P_{23,zy}(t) &= P(X(t+1)=y|Z(t)=z) \nonumber \\ & = {2N(t+1)\atopwithdelims ()2N(t+1)y} [z(1-\mu )]^{2N(t+1)y} [1-z(1-\mu )]^{2N(t+1)(1-y)} \end{aligned}$$

is a transition probability of Steps 2 and 3 combined. Moreover,

A.6 $$\begin{aligned} {{{\mathcal {Z}}}}(t) = \{0,\frac{1}{2N_e(t)},\ldots ,\frac{2N_e(t)-1}{2N_e(t)},1\} \end{aligned}$$

is the set of possible frequencies of *a* in the breeding pool formed in Step 1. It follows from (A.1), (A.2), and formulas for expected values and variances of hypergeometric and binomial distributions, that

A.7 $$\begin{aligned} & E[Z(t)|X(t)=x] = x, \end{aligned}$$

A.8 $$\begin{aligned} & \mathrm{Var}[Z(t)|X(t)=x] = \frac{x(1-x)}{2N_e(t)} \cdot \frac{2N(t)-2N_e(t)}{2N(t)-1}, \end{aligned}$$

A.9 $$\begin{aligned} & E[X(t+1)|Z(t)=z] = z(1-\mu ), \end{aligned}$$

and

A.10 $$\begin{aligned} \mathrm{Var}[X(t+1)|Z(t)=z] = \frac{z(1-\mu ) [1-z(1-\mu )]}{2N(t+1)}. \end{aligned}$$

After some computations, this leads to

$$\begin{aligned} \begin{array}{rcl} E[X(t+1)|X(t)=x] & =& E\{E[X(t+1)|Z(t)]|X(t)=x\}\\ & =& (1-\mu )E[Z(t)|X(t)=x]\\ & =& (1-\mu )x, \end{array} \end{aligned}$$

in agreement with (29), and

A.11 $$\begin{aligned} \begin{array}{rcl} \mathrm{Var}[X(t+1)|X(t)=x] & =& \mathrm{Var}\{E[X(t+1)|Z(t)]|X(t)=x\}\\ & +& E\{\mathrm{Var}[X(t+1)|Z(t)]|X(t)=x\}\\ & =& \frac{(1-\mu )^2 x(1-x)}{2N_e(t)} \cdot \frac{2N(t)-2N_e(t)}{2N(t)-1} \left( 1-\frac{1}{2N(t+1)}\right) \\ & +& \frac{x(1-\mu )[1-x(1-\mu )]}{2N(t+1)}\\ & =& [1-d(t)] c(t) \frac{(1-\mu )^2 x(1-x)}{2N_e(t)} \\ & +& d(t+1) \frac{x(1-\mu )[1-x(1-\mu )]}{2N_e(t+1)}, \end{array} \end{aligned}$$

with

A.12 $$\begin{aligned} c(t) = \frac{1-1/[2N(t+1)]}{1-1/[2N(t)]} \end{aligned}$$

and *d*(*t*) as defined in (31). Since *c(t)≈ 1* for all but very small populations, (A.11) is consistent with (30).

### A.2 Dioecious Population

For a dioecious populaton, we introduce $\boldsymbol{ Z}(t)=(Z_m(t),Z_f(t))$, where $Z_m(t)$ and $Z_f(t)$ is the fraction of gene copies with allele *a* in the male and female breeding pools, formed in Step 1 of the reproduction cycle of Section 2.2. Suppose *X(t)=x*. Since gene copies are drawn randomly without replacement when the male and female breeding pools are formed, it follows that the numbers $2N_{em}Z_m(t)$ and $2N_{ef}Z_f(t)$ of gene copies among the male and female breeders with allele *a*, have a joint hypergeometric distribution

A.13 $$\begin{aligned} (2N_{em}Z_m(t),2N_{ef}Z_f(t))|X(t)=x \sim \mathrm{Hyp}(2N(t);(2N_{em}(t),2N_{ef}(t)),x). \end{aligned}$$

Let $\boldsymbol{ X}(t+1)=(X_m(t+1), X_f(t+1))$, where $X_m(t+1)$ and $X_f(t+1)$ refer to the fraction of gene copies drawn from the male and female breedings pools in Step 2 of the reproduction cycle, that have allele *a* after the mutation events in Step 3. Suppose $Z_m(t)=z_m$ and $Z_f(t)=z_f$. Since gene copies are drawn independently from the male and female gene pools, and then mutate independently, the number of gene copies $N(t+1)X_m(t+1)$ and $N(t+1)X_f(t+1)$ with allele *a* are independent, conditionally on $z_m$ and $z_f$, with binomial distributions

A.14 $$\begin{aligned} \begin{array}{rcl} N(t+1)X_m(t+1)|Z_m(t)=z_m & \sim & \mathrm{Bin}(N(t+1);z_m(1-\mu )),\\ N(t+1)X_f(t+1)|Z_f(t)=z_f & \sim & \mathrm{Bin}(N(t+1);z_f(1-\mu )). \end{array} \end{aligned}$$

Once the gene copies have been drawn from the male and female breeding pools, and possiby mutated, the frequency

A.15 $$\begin{aligned} X(t+1) = \frac{1}{2}[X_m(t+1)+X_f(t+1)] \end{aligned}$$

of *a* in generation *t+1* is the average of the frequency of *a* among the gene copies drawn from the male and female breeding pools.

In order to find expressions for the transition probabilities (28) of *X(· )* for a dioecious population, it is convenient to introduce

A.16 $$\begin{aligned} \begin{array}{rcl} {{{\mathcal {Z}}}}(t) & =& {{{\mathcal {Z}}}}_m(t)\times {{{\mathcal {Z}}}}_f(t)\\ & =& \{0,\frac{1}{2N_{em}(t)},\ldots ,\frac{2N_{em}(t)-1}{2N_{em}(t)},1\} \times \{0,\frac{1}{2N_{ef}(t)},\ldots ,\frac{2N_{ef}(t)-1}{2N_{ef}(t)},1\} \end{array} \end{aligned}$$

for the possible values that the fraction of allele *a* can take in the two breeding pools in Step 1 of the reproduction cycle of Section 2.2, and

A.17 $$\begin{aligned} {{{\mathcal {Y}}}}(t) = {{{\mathcal {Y}}}}_m(t)\times {{{\mathcal {Y}}}}_f(t) = \{0,\frac{1}{N(t+1)},\ldots ,\frac{N(t+1)-1}{N(t+1)},1\}^2. \end{aligned}$$

for the set of values that the fraction of allele *a* can take among the paternally and maternally inherited gene copies, after Step 3 of the reproduction cycle. It follows from (A.13)-(A.15) that the transition probabilities (28) satisfy

A.18 $$\begin{aligned} P_{xy}(t) = \sum _{{\boldsymbol{z}}\in {{{\mathcal {Z}}}}(t)} \sum _{{\boldsymbol{y}}\in {{{\mathcal {Y}}}}(t)} P_{1x{\boldsymbol{z}}}(t)P_{23,{\boldsymbol{z}}{\boldsymbol{y}}}(t)P_{3{\boldsymbol{y}}y}(t) \end{aligned}$$

where $$\boldsymbol{ z}=(z_m,z_f)$$ and

A.19 $$\begin{aligned} \begin{array}{rcl} P_{1x{\boldsymbol{z}}}(t) & =& P(\boldsymbol{ Z}(t)=\boldsymbol{ z}|X(t)=x)\\ & =& \frac{{2N(t)x\atopwithdelims ()2N_{em}(t)z_m}\cdot {2N(t)(1-x)\atopwithdelims ()2N_{em}(t)(1-z_m)} \cdot {2N(t)x-2N_{em}(t)z_m\atopwithdelims ()2N_{ef}(t)z_f} \cdot {2N(t)(1-x) - 2N_{em}(1-z_m)\atopwithdelims ()2N_{ef}(t)(1-z_f)}}{{2N(t)\atopwithdelims ()2N_{em}(t)}\cdot {2N(t)-2N_{em}(t)\atopwithdelims ()2N_{ef}(t)}} \end{array} \end{aligned}$$

are transition probabilities from Step 1 of the reproduction cycle, when breeding pools are formed, whereas $\boldsymbol{ y}=(y_m,y_f)$ and

A.20 $$\begin{aligned} \begin{array}{rcl} P_{23,{\boldsymbol{z}}{\boldsymbol{y}}}(t) & =& P(\boldsymbol{ X}(t+1)=\boldsymbol{ y}|\boldsymbol{ Z}(t)=\boldsymbol{ z})\\ & =& {N(t)\atopwithdelims ()N(t)y_m} [(1-\mu )z_m]^{N(t)y_m} [1 - (1-\mu )z_m]^{N(t)(1-y_m)}\\ & \cdot & {N(t)\atopwithdelims ()N(t)y_f} [(1-\mu )z_f]^{N(t)y_f} [1 - (1-\mu )z_f]^{N(t)(1-y_f)} \end{array} \end{aligned}$$

are transition probabilities of Steps 2 and 3 combined, when gene copies are drawn from the male and female breeding pools and then mutate. Finally,

A.21 $$\begin{aligned} P_{3{\boldsymbol{y}}y}(t) = P(X(t+1)=y|\boldsymbol{ X}(t+1)=\boldsymbol{ y}) = 1(y=\frac{y_m+y_f}{2}) \end{aligned}$$

are transition probabilities for the frequency of allele *a* in Step 3 of the reproduction cycle that correspond to merging the paternally and maternally inherited gene copies into one population, according to (A.15).

It is possible to verify formula (29) for the expectation of *X(t+1)*, conditionally on *X(t)=x*, analogously as for a monoecious population (cf. Appendix A.1). In order to find a formula for the variance of *X(t+1)*, conditionally on *X(t)=x*, for a dioecious population, we notice that

A.22 $$\begin{aligned} \begin{array}{rcl} \mathrm{Var}[X(t+1)|X(t)=x] & =& \frac{1}{4} \mathrm{Var}[X_m(t+1)|X(t)=x]\\ & +& \frac{1}{4}\mathrm{Var}[X_f(t+1)|X(t)=x]\\ & +& \frac{1}{2}\mathrm{Cov}[X_m(t+1),X_f(t+1)|X(t)=x], \end{array} \end{aligned}$$

and in order to proceed we need to find expressions for the three terms on the right-hand side of (A.22). Starting with the first two terms, we repeat the argument that was used to derive formula (A.11) of Appendix A.1, and obtain

A.23 $$\begin{aligned} \begin{array}{rcl} \mathrm{Var}[X_m(t+1)|X(t)=x] & =& \frac{(1-\mu )^2x(1-x)}{2N_{em}(t)}\cdot \frac{2N(t)-2N_{em}(t)}{2N(t)-1}\left( 1-\frac{1}{N(t+1)}\right) \\ & +& \frac{x(1-\mu )[1-x(1-\mu )]}{N(t+1)},\\ \mathrm{Var}[X_f(t+1)|X(t)=x] & =& \frac{(1-\mu )^2x(1-x)}{2N_{ef}(t)}\cdot \frac{2N(t)-2N_{ef}(t)}{2N(t)-1}\left( 1-\frac{1}{N(t+1)}\right) \\ & +& \frac{x(1-\mu )[1-x(1-\mu )]}{N(t+1)}. \end{array} \end{aligned}$$

It is more delicate to find a formula for the third term on the right-hand side of (A.22). After some computations, it follows from (A.13) that

A.24 $$\begin{aligned} \mathrm{Cov}[Z_m(t),Z_f(t)|X(t)=x] = -\frac{x(1-x)}{2N(t)-1}. \end{aligned}$$

Together with (A.14), this gives

A.25 $$\begin{aligned} \begin{array}{l} \mathrm{Cov}[X_m(t+1),X_f(t+1)|X(t)=x]\\ \,\,\,\,\,\,\,\, = \mathrm{Cov}[E(X_m(t+1)|Z_m(t)),E(X_f(t+1)|Z_f(t))|X(t)=x]\\ \,\,\,\,\,\,\,\, + E[\mathrm{Cov}(X_m(t+1),X_f(t+1)|Z_m(t),Z_f(t))|X(t)=x]\\ \,\,\,\,\,\,\,\, = (1-\mu )^2 \mathrm{Cov}[Z_m(t),Z_f(t)|X(t)=x] + 0\\ \,\,\,\,\,\,\,\, = - (1-\mu )^2 \frac{x(1-x)}{2N(t)-1}. \end{array} \end{aligned}$$

Inserting (A.23) and (A.25) into (A.22), and making use of (2) and (31), we obtain

A.26 $$\begin{aligned} \begin{array}{rcl} \mathrm{Var}[X(t+1)|X(t)=x] & =& \frac{(1-\mu )^2x(1-x)}{2N(t)-1} \left\{ \frac{1}{4} \left[ \frac{N(t)-N_{em}(t)}{N_{em}(t)} + \frac{N(t)-N_{ef}(t)}{N_{ef}(t)}\right] (1-\frac{1}{N(t+1)}) - \frac{1}{2}\right\} \\ & +& \frac{x(1-\mu )[1-x(1-\mu )]}{2N(t+1)}\\ & =& \frac{(1-\mu )^2x(1-x)}{2N(t)-1}\left[ \left( \frac{N(t)}{N_e(t)} - \frac{1}{2}\right) (1-\frac{1}{N(t+1}) - \frac{1}{2}\right] \\ & +& \frac{x(1-\mu )[1-x(1-\mu )]}{2N(t+1)}\\ & =& [1-\tilde{d}(t)]c(t) \frac{(1-\mu )^2 x(1-x)}{2N_e(t)}\\ & +& d(t+1) \frac{x(1-\mu )[1-x(1-\mu )]}{2N_e(t+1)}, \end{array} \end{aligned}$$

with *c*(*t*) and *d*(*t*) defined in (A.12) and (31), respectively, and

A.27 $$\begin{aligned} \tilde{d}(t) = d(t) + \frac{1}{2N(t+1)-1}. \end{aligned}$$

Note that (A.26) is very similar to the variance formula (A.11) for the allele frequency change of a monoecious population. The only difference is that *d*(*t*) in (A.11) is replaced by $\tilde{d}(t)$ in (A.26).

## B Populations Under Equilibrium Conditions

### B.1 Spectral properties of $\boldsymbol{ P}$

It is assumed in this section that the population is monoecious and of constant size, as in Section 7. We will verify formula (63) for the third largest eigenvalue $\lambda _2$ of $\boldsymbol{ P}$, with an associated right eigenvector $\boldsymbol{ r}_2$ that corresponds to the function $r_2(x)=x^2+cx$, with *c* defined below. The argument for obtaining explicit expressions for $\boldsymbol{ r}_q$ and $r_q(x)$ when *q>2* is similar, but more complicated.

Recall that $\boldsymbol{ r}_1=\boldsymbol{ g}_{\mathrm{id}}$ (or $r_1(x)=g_{\mathrm{id}}(x)=x$) is the right eigenvector corresponding to the second largest eigenvalue $\lambda _1=1-\mu$ of $\boldsymbol{ P}$. We will also make use of the functions $g_1(x)=1$, $g_{{\bar{H}}}(x)=x^2$ and $g_{H}(x)=x(1-x)=g_{\mathrm{id}}(x)-g_{{\bar{H}}}(x)$, defined in Examples 4 and 5. Given two column vectors $\boldsymbol{ g}$ and $\boldsymbol{ h}$ of length *2N+1* we write $\boldsymbol{ g}\boldsymbol{ h}$ for the column vector obtained by componentwise multiplication of the elements of $\boldsymbol{ g}$ and $\boldsymbol{ h}$.

Our argument is based on the fact that $\boldsymbol{ P}\boldsymbol{ g}_H$ corresponds to a time recursion for expected gene diversity. It follows from formula (51), for the time recursion of the expected gene diversity of a monoecious population, and the simpifying expressions (74) for the coefficients of this recursion when the population is of constant size and in equilibriium, that

B.1 $$\begin{aligned} \begin{array}{rcl} \boldsymbol{ P}\boldsymbol{ g}_{H} & =& \boldsymbol{ P}\boldsymbol{ g}_{\mathrm{id}} - \boldsymbol{ P}\boldsymbol{ g}_{{\bar{H}}}\\ & =& (1-\mu )[1-d/(2N_e)] \boldsymbol{ g}_{\mathrm{id}} [\boldsymbol{ g}_1 - (1-\mu )\boldsymbol{ g}_{\mathrm{id}}]\\ & -& (2N_e)^{-1}(1-d)(1-\mu )^2 \boldsymbol{ g}_{\mathrm{id}} (\boldsymbol{ g}_1 - \boldsymbol{ g}_{\mathrm{id}})\\ & =& (1-\mu ) \boldsymbol{ g}_{\mathrm{id}} - a \boldsymbol{ g}_{{\bar{H}}} - b \boldsymbol{ g}_{\mathrm{id}}, \end{array} \end{aligned}$$

with

$$\begin{aligned} \begin{array}{rcl} a & =& (1-\mu )^2 (1-1/(2N_e)),\\ b & =& (1-\mu ) [1-\mu (1-d)]/(2N_e). \end{array} \end{aligned}$$

In the last step of (B.1) we made use of the fact that $\boldsymbol{ g}_{\mathrm{id}}\boldsymbol{ g}_1 = \boldsymbol{ g}_{\mathrm{id}}$ and $\boldsymbol{ g}_{\mathrm{id}}\boldsymbol{ g}_{\mathrm{id}} = \boldsymbol{ g}_{{\bar{H}}}$. It follows from (29) and (63), with *q=1*, that

B.2 $$\begin{aligned} \boldsymbol{ P}\boldsymbol{ g}_{\mathrm{id}} = \lambda _1 \boldsymbol{ g}_{\mathrm{id}} = (1-\mu ) \boldsymbol{ g}_{\mathrm{id}}. \end{aligned}$$

Hence we can rewrite (B.1) as

B.3 $$\begin{aligned} \boldsymbol{ P}\boldsymbol{ g}_{{\bar{H}}} = a \boldsymbol{ g}_{{\bar{H}}} + b \boldsymbol{ g}_{\mathrm{id}}. \end{aligned}$$

Suppose the right eigenvector of the third largest eigenvalue $\lambda _2$ is $\boldsymbol{ r}_2 = \boldsymbol{ g}_{{\bar{H}}} + c \boldsymbol{ g}_{\mathrm{id}}$ for some constant *c*, or $r_2(x)=x^2+cx$. Formulas (B.2) and (B.3) imply that

B.4 $$\begin{aligned} \begin{array}{rcl} \boldsymbol{ P}\boldsymbol{ r}_2 & =& \boldsymbol{ P}(\boldsymbol{ g}_{{\bar{H}}} + c \boldsymbol{ g}_{\mathrm{id}})\\ & =& a \boldsymbol{ g}_{{\bar{H}}} + [b + (1-\mu )c] \boldsymbol{ g}_{\mathrm{id}}\\ & =& a [ \boldsymbol{ g}_{{\bar{H}}} + a^{-1}(b + (1-\mu )c ) \boldsymbol{ g}_{\mathrm{id}}]\\ & =& a (\boldsymbol{ g}_{{\bar{H}}} + c \boldsymbol{ g}_{\mathrm{id}})\\ & =& a \boldsymbol{ r}_2, \end{array} \end{aligned}$$

if *c=[b+(1-μ )c]/a*, or equivalently

B.5 $$\begin{aligned} c = -\frac{b}{1-\mu - a} = -\frac{1-\mu (1-d)}{1 + 2\theta _e - \mu }. \end{aligned}$$

This proves that $\boldsymbol{ r}_2$ is a right eigenvector of $\boldsymbol{ P}$ with eigenvalue $\lambda _2 = a$ as in (66), if *c* is chosen according to (B.5). Note that *c ≈ 0* when $\theta _e$ is large (mutations dominate over genetic drift) whereas *c≈ -1* when $\theta _e$ is small (genetic drift dominates over mutations). This correspond to scenarios when $r_2(x)\approx g_{{\bar{H}}}(x)=x^2$ and $r_2(x)\approx -g_{H}(x) =-x(1-x)$, respectively.

### B.2 Expected Number of Alleles Under Equilibrium

In this section we will investigate more closely the properties of $E[A_n(\infty )]$, the expected number of alleles at equilibrium, as a function of the rescaled mutation rate $\theta _e= N_e\mu$. To this end we will make use of two approximations of $E[A_n(\infty )]$. The first one is Ewens’ sampling formula

B.6 $$\begin{aligned} E[A_n(\infty )] \approx 4\theta _e\sum _{k=0}^{2N-1} \frac{1}{4\theta _e+ k} := s(\theta _e;N) = s(\theta _e), \end{aligned}$$

introduced in (71), and the second one is

B.7 $$\begin{aligned} E[A_n(\infty )] \approx 4\theta _e \int _{1/(2N)}^1 (1-x)^{4\theta _e - 1} x^{-1}dx := m(\theta _e;N) = m(\theta _e), \end{aligned}$$

introduced in (73). These two functions $s(\theta _e)$ and $m(\theta _e)$ are defined for $0<\theta _e \le N_e\mu _{\text{ max }}$, where $\mu _{\text{ max }}$ is an upper bound on the mutation probability, specified below. The following two propositions give some properties of *s(· )* and *m(· )*, respectively.

#### Proposition 1

Suppose the equilibrium conditions of Section 7 hold, with a mutation probability that satisfies

B.8 $$\begin{aligned} 0<\mu \le 1 = \mu _{\text{ max }}. \end{aligned}$$

The approximate expected number of alleles $s(\theta _e)$ at equilibrium, defined in (B.6), is then a strictly increasing function of $\theta _e$ on $(0,N_e]$ for each fixed *N*. It satisfies

B.9 $$\begin{aligned} \begin{array}{rcl} s(\theta _e) & =& 1 + 4\theta _e\sum _{k=1}^{2N-1} k^{-1} + o(\theta _e)\\ & \approx & 1 + 4\theta _e\log (2N) + o(\theta _e)\\ & =: & 1 + c\theta _e+ o(\theta _e) \end{array} \end{aligned}$$

as $\theta _e\rightarrow 0$ in such a way that *N* is kept fixed, and the approximation of the second step holds for large *N*. Moreover

B.10 $$\begin{aligned} \begin{array}{rcl} s(\theta _e) & \sim & 4\theta _e\log [(2N + 4\theta _e)/(4\theta _e)]\\ & \approx & 4\theta _e\log [(2d\mu )^{-1}]\\ & =: & C\theta _e\end{array} \end{aligned}$$

as $\theta _e\rightarrow \infty$ in such a way that *μ* and $d=N_e/N$ in (88) are kept fixed. The meaning of (B.10) is that $s(\theta _e)/(C\theta _e)\rightarrow 1$ as $\theta _e\rightarrow \infty$, and the approximation of the second step of this equation requires that $2d\mu \ll 1$.

#### Proof of Proposition 1

The proposition follows easily from the definition of $s(\theta _e)$ in (B.6), viewing it as a Riemann sum approximation of an integral. *□*

#### Proposition 2

Suppose the equilibrium conditions of Section 7 hold, with a mutation probability that satisfies

B.11 $$\begin{aligned} 0<\mu \le \frac{e^{-2}}{4d} = \mu _{\text{ max }}, \end{aligned}$$

and with $d=N_e/N$ as in (88). The diffusion approximation $m(\theta _e)$ of the expected number of alleles at equilibrium, defined in (B.7), is then a strictly increasing function of $\theta _e$ on $(0,N_e\mu _{\text{ max }}]$ for each fixed *N*. It satisfies

B.12 $$\begin{aligned} m(\theta _e) = 1 + 4\theta _e\int _{1/(2N)}^1 x^{-1}e^{-x}dx + o(\theta _e) =: 1 + c\theta _e+ o(\theta _e) \end{aligned}$$

as $\theta _e\rightarrow 0$ in such a way that *N* is kept fixed. Moreover,

B.13 $$\begin{aligned} m(\theta _e) \sim 4\theta _e\int _{2\mu d}^\infty y^{-1}e^{-y}dy =: C\theta _e \end{aligned}$$

as $\theta _e\rightarrow \infty$ in such a way that *μ* and $d=N_e/N$ are kept fixed, meaning that $m(\theta _e)/(C\theta _e)\rightarrow 1$ as $\theta _e\rightarrow \infty$.

#### Proof of Proposition 2

In order to prove that *m(· )* is strictly increasing we find its derivative by differentiate under the integral sign in (B.7). This yields

B.14 $$\begin{aligned} \begin{array}{rcl} m^\prime (\theta _e) & =& \int _{1/(2N)}^1 4x^{-1}(1-x)^{4\theta _e-1} [1 + 4\theta _e\log (1-x)]dx\\ & =& \frac{m(\theta _e)}{\theta _e} + 16\theta _e\int _{1/(2N)}^1 x^{-1}(1-x)^{4\theta _e-1} \sum _{n=1}^\infty \frac{(-x)^n}{n} dx\\ & =& \frac{m(\theta _e)}{\theta _e} + \sum _{n=1}^\infty (-1)^n I_n, \end{array} \end{aligned}$$

where in second step we Taylor expanded *łog (1-x)*, and in the last step we introduced

B.15 $$\begin{aligned} I_n = \frac{16\theta _e}{n} \int _{1/(2N)}^1 x^{n-1}(1-x)^{4\theta _e-1}dx. \end{aligned}$$

The the last term of the right-hand side of (B.14) is an alternating series, with $I_n$ decreasing with *n*, it follows that

B.16 $$\begin{aligned} \begin{array}{rcl} m^\prime (\theta _e) & \ge & \frac{m(\theta _e)}{\theta _e} - I_1 \\ & =& \frac{m(\theta _e)}{\theta _e} - 4 (1-\frac{1}{2N})^{4\theta _e}\\ & \ge & \frac{m(\theta _e)}{\theta _e} - 4. \end{array} \end{aligned}$$

Hence in order to prove that *m(· )* is increasing it suffices to verify that

B.17 $$\begin{aligned} k(\theta _e) = \frac{m(\theta _e)}{4\theta _e} \ge 1. \end{aligned}$$

By a variable substitution $y=4\theta _ex$ in (B.7), it follows that

B.18 $$\begin{aligned} k(\theta _e) = \int _{2\mu d}^{4\theta _e} y^{-1}(1-\frac{y}{4\theta _e})^{4\theta _e- 1} dy. \end{aligned}$$

If $0<\theta _e\le 1/4$, formula (B.17) follows from (B.18), since

$$\begin{aligned} k(\theta _e) \ge \int _{2\mu d}^{4\theta _e} y^{-1}dy = \log \left( \frac{4\theta _e}{2\mu d}\right) = \log (2N) \ge 1. \end{aligned}$$

We then make use of (B.18) a second time, when $\theta _e\ge 1/4$, and find that

$$\begin{aligned} \begin{array}{rcl} k(\theta _e) & \ge & \int _{2\mu d}^{4\theta _e} y^{-1}(1-y)dy\\ & \ge & \frac{1}{2}\int _{2\mu d}^{1/2} y^{-1}dy\\ & \ge & \frac{1}{2}\log [1/(4\mu d)] \\ & \ge & 1, \end{array} \end{aligned}$$

where in the last step we inserted (B.11). This completes the proof that *m(· )* is increasing. Formula (B.13) follows from (B.18), since

$$\begin{aligned} \begin{array}{rcl} m(\theta _e) & =& 4\theta _e\int _{2\mu d}^{4\theta _e} y^{-1}(1-\frac{y}{4\theta _e})^{4\theta _e- 1} dy\\ & \sim & 4\theta _e\int _{2\mu d}^{4\theta _e} y^{-1}e^{-y}dy\\ & \sim & 4\theta _e\int _{2\mu d}^\infty y^{-1}e^{-y}dy \end{array} \end{aligned}$$

as $\theta _e\rightarrow \infty$. It remains to prove (B.12). To this end we insert the Taylor expansion

$$\begin{aligned} x^{-1} = \frac{1}{1-(1-x)} = \sum _{n=0}^\infty (1-x)^n, \quad 0< x \le 1, \end{aligned}$$

into (B.7) and find for small $\theta _e>0$ that

B.19 $$\begin{aligned} \begin{array}{rcl} m(\theta _e) & =& \sum _{n=0}^\infty \int _{1/(2N)}^1 (1-x)^n 4\theta _e(1-x)^{4\theta _e-1}dx\\ & =& 4\theta _e\sum _{n=0}^\infty \frac{(1-\frac{1}{2N})^{4\theta _e+ n}}{4\theta _e+ n}\\ & =& (1-\frac{1}{2N})^{4\theta _e} \left[ 1 + 4\theta _e\sum _{n=1}^\infty \frac{(1-\frac{1}{2N})^n}{4\theta _e+n}\right] \\ & \approx & 1 + 4\theta _e\sum _{n=1}^\infty \frac{(1-\frac{1}{2N})^n}{n}\\ & \approx & 1 + 4\theta _e\int _{1/(2N)}^\infty x^{-1} (1-\frac{1}{2N})^{2Nx}dx\\ & \approx & 1 + 4\theta _e\int _{1/(2N)}^\infty x^{-1}e^{-x}dx\\ & =& 1 + c\theta _e, \end{array} \end{aligned}$$

where the three approximations $a(\theta _e)\approx b(\theta _e)$ of (B.19) mean that $[a(\theta _e)-1]/[b(\theta _e)-1]$ converges to 1 as $\theta _e\rightarrow 0$. *□*

The definitions of the constants *c=c(N)* and *C=C(μ d)* of Propositions 1 and 2 are somewhat different. However, it is easy to see that the two definitions of *c*(*N*) agree in the limit of large *N*, whereas the two definitions of *C(μ d)* agree in the limit of small *μ d*. The parts of Propositions 1 and 2 that concern the behaviour of $E[A_n(\infty )]$ for small $\theta _e$, together with the properties of expected gene diversity *E[H(∞ )]* at equilibrium for small $\theta _e$ (cf. Example 16), have interesting consequences for the average frequency of rare alleles (cf. (15) of Example 3), as the following corollary shows:

#### Corollary 1

The average frequency ${\bar{x}}={\bar{x}}(\infty )$ of rare alleles (frequency $\le \eta$ for a fixed *0<η <1*) under equilibrium, when at least one such allele exists ($S_{g_{\tiny \mathrm{ra}}}(\infty )\ge 1$), satisfies

B.20 $$\begin{aligned} E({\bar{x}}(\infty )|S_{g_{\tiny \mathrm{ra}}}(\infty )\ge 1) = \frac{2}{c} + o(\theta _e) \end{aligned}$$

as $\theta _e\rightarrow 0$ in such a way that *N* is kept fixed, and with *c=c(N)* the constant defined in (B.9) or (B.12), depending on whether the approximation of Proposition 1 or 2 is used.

**Proof.** Assume $\theta _e>0$ is small. Let $A=A_n(\infty )$ refer to the number of alleles at equilibrium. Since $\theta _e$ is small, with a high probability the most abundant allele has a frequency that either equals 1 (if *A=1*) or is close to 1 (if *A>1*), and in the latter case all remaining *A-1* alleles have a small frequency. Therefore, with a high probability one of the *A* alleles is common whereas the other $S_{g_{\tiny \mathrm{ra}}}(\infty )=A-1$ alleles (if *A>1*) are rare. We number allele frequencies $x_1,\ldots ,x_A$, so that $x_A$ is the frequency of the common allele, whereas $x_1,\ldots ,x_{A-1}$ are the frequencies of the rare alleles. Define the average allele frequency among the rare alleles as

B.21 $$\begin{aligned} {\bar{x}}= \frac{1}{A-1}\sum _{k=1}^{A-1} x_k, \quad \text{ when } A>1. \end{aligned}$$

Let $p=P(A>1)=p(\theta _e)$ be the probability of having at least one rare allele. Then

B.22 $$\begin{aligned} E(A) = (1-p)\cdot 1 + p E(A|A>1) = 1 + p E(A-1|A>1) = 1 + c\theta _e+ o(\theta _e) \end{aligned}$$

as $\theta _e\rightarrow 0$, where in the last step we made use of (B.9) or (B.12). The expected gene diversity at equilibrium equals

B.23 $$\begin{aligned} \begin{array}{rcl} E[H(\infty )] & =& p E\left[ \sum _{k=1}^A x_k(1-x_k)|A>1\right] \\ & =& p \{E\left[ \sum _{k=1}^{A-1} x_k(1-x_k)|A>1\right] + E[x_A(1-x_A)|A>1]\}\\ & \sim & p\{E\left[ \sum _{k=1}^{A-1} x_k|A>1\right] + E\left[ \sum _{k=1}^{A-1} x_k|A>1\right] \} \\ & =& p E[2(A-1){\bar{x}}|A>1]\\ & \sim & 4\theta _e\end{array} \end{aligned}$$

as $\theta _e\rightarrow 0$, where in the last step we made use of (76), and with $a(\theta _e)\sim b(\theta _e)$ referring to $a(\theta _e)/b(\theta _e)\rightarrow 1$ as $\theta _e\rightarrow 0$. Given that at least one rare allele exists (*A>1*), the conditional probability of having more than one rare allele (*A>2*) converges to 0 as $\theta _e\rightarrow 0$. This implies that $E[(A-1){\bar{x}}|>A-1]\sim E(A-1|A>1)E({\bar{x}}|A-1)$ as $\theta _e\rightarrow 0$. It then follows from (B.22) and (B.23) that

$$\begin{aligned} \begin{array}{rcl} E({\bar{x}}|A>1) & =& \frac{E[A-1|A>1]E({\bar{x}}|A>1)}{E[A-1|A>1]}\\ & \sim & \frac{E[(A-1){\bar{x}}|A>1]}{E(A-1|A>1)}\\ & \sim & \frac{2\theta _e/ p}{c\theta _e/ p} \\ & =& \frac{2}{c}, \end{array} \end{aligned}$$

in agreement with (B.20). *□*

### B.3 Transition of Expected Number of Alleles from One Equilibrium to Another

In this appendix we study a population that experiences a sudden population change, as described in Section 8. In more detail, we will use a branching process approximation of the absolute frequency fluctuation $2N(\cdot )X(\cdot )$ of one allele *a*, to motivate that the expected number $E[A_n(t)]$ of alleles in (91) transitions from one equilibrium $E[A_n(0)]$ to another, $E[A_n(\infty )]$, in $O(\mu ^{-1})$ generations.

Let *Z*(*t*) refer to the number of offspring of one (arbitrarily chosen) individual of generation *t*. The approximate distribution

B.24 $$\begin{aligned} Z(t) {\mathop {\sim }\limits ^{\approx }} \left\{ \begin{array}{ll} (1-d)\delta _0 + d \mathrm{Po}(m), & t\ne 0,\\ (1-d)\delta _0 + d \mathrm{Po}(m_c), & t= 0, \end{array}\right. \end{aligned}$$

of this number of offspring, for a large monoecious population, is a mixture of a point mass $\delta _0$ at 0, and a Poisson distribution with expected value

$$\begin{aligned} \begin{array}{rcll} m & =& (1-\mu )/d, & t\ne 0,\\ m_c & =& (1-\mu )\theta /(\theta _0 d), & t=0. \end{array} \end{aligned}$$

The mixing fraction $d=N_{e0}/N_0=N_e/N$ is the ratio between the effective and census sizes, having the same value before and after the population size change, whereas $\theta _0=N_0\mu$ and $\theta =N\mu$ are rescaled mutation rates before and after the population size change. It follows from (B.24) that the probability generating function of *Z*(*t*) is approximated by

B.25 $$\begin{aligned} \sum _{z=0}^\infty P[Z(t)=z] s^z \approx \left\{ \begin{array}{ll} G(s) = 1-d + d\exp [m(s-1)], & t\ne 0,\\ H(s) = 1-d + d\exp [m_c(s-1)], & t= 0. \end{array}\right. \end{aligned}$$

Since *E[Z(t)]=1-μ < 1* for *t≠ 0*, the number of copies 2*N*(*t*)*X*(*t*) of allele *a* at time *t = 0,1,…* can be approximated by a subcritical branching process that gets extinct with probability 1. From this it follows that absorption probabilities of *X(· )* that appear in (91) can be approximated by extinction probabilities of the branching process. This corresponds to putting

B.26 $$\begin{aligned} \begin{array}{rcl} (\boldsymbol{ P}^k)_{\varepsilon 0} & =& P_{\varepsilon 0}^k \approx G_k(0),\\ (\boldsymbol{ P}_0^l \boldsymbol{ P}_c \boldsymbol{ P}^k)_{\varepsilon _0 0} & \approx & G_l \circ H \circ G_k (0) \end{array} \end{aligned}$$

in this equation, where *∘* refers to function composition, whereas $G_k$ is the *k*-fold composition of *G*. The corresponding non-absorption probabilities are approximated by

B.27 $$\begin{aligned} \begin{array}{rcl} 1 - P_{\varepsilon 0}^k & \approx & 1 - G_k(0) = {\bar{G}}_k(1),\\ 1 - (\boldsymbol{ P}_0^l \boldsymbol{ P}_c \boldsymbol{ P}^k)_{\varepsilon _0 0} & \approx & 1 - G_l \circ H \circ G_k (0) = {\bar{G}}_l \circ {\bar{H}}\circ {\bar{G}}_k (1), \end{array} \end{aligned}$$

where

$$\begin{aligned} \begin{array}{rcl} {\bar{G}}(s) & =& 1 - G(1-s) = d[1-\exp (-ms)],\\ {\bar{H}}(s) & =& 1 - H(1-s) = d[1-\exp (-m_c s)] = {\bar{G}}(\theta s/\theta _0), \end{array} \end{aligned}$$

and ${\bar{G}}_k$ is the *k*-fold composition of ${\bar{G}}$. The approximations in (B.26) and (B.27) are only accurate when *2Nμ* is not too small, so that the branching process is not close to critical. Inserting (B.27) into (91) we find that

B.28 $$\begin{aligned} \begin{array}{rcl} E[A_n(t)] & \approx & 2\theta _0 \sum _{k=0}^\infty {\bar{G}}_k \{ {\bar{G}}[\frac{\theta }{\theta _0} {\bar{G}}_{t-1}(1)]\} + 2\theta \sum _{k=0}^{t-1} {\bar{G}}_k(1)\\ & =& 2\theta _0 \sum _{k=1}^\infty {\bar{G}}_k [\frac{\theta }{\theta _0} {\bar{G}}_{t-1}(1)] + 2\theta \sum _{k=0}^{t-1} {\bar{G}}_k(1)\\ & =& S_1(t;\theta _0,\theta ) + S_2(t;\theta ), \end{array} \end{aligned}$$

where $S_1(t;\theta _0,\theta )\rightarrow 0$ and $S_2(t;\theta )\rightarrow E[A_n(\infty )]$ as $t\rightarrow \infty$. Since

$$\begin{aligned} E[A_n(\infty )] \approx S_1(t;\theta ,\theta ) + S_2(t;\theta ), \end{aligned}$$

it follows that

B.29 $$\begin{aligned} \frac{|E[A_n(t)]-E[A_n(\infty )]|}{E[A_n(\infty )]} \approx \frac{|S_1(t;\theta _0,\theta ) -S_1(t;\theta ,\theta )|}{S_1(t;\theta ,\theta ) + S_2(t;\theta )} \le \eta \end{aligned}$$

for any *η>0*, and $t\ge t_0 = t_0(\eta ,\theta _0,\theta ,d)$. Hence $t_0$ tells how many generations it takes for the expected number of alleles to reach the new equilibrium, with a relative error of at most *η*. In order to find an upper bound of $t_0$, we make use of the fact that ${\bar{G}}$ is a concave function with ${\bar{G}}(0)=0$ and ${\bar{G}}^\prime (0)=1-\mu$, so that

$$\begin{aligned} \frac{{\bar{G}}(s)}{s} \nearrow 1-\mu \text{ as } s\searrow 0. \end{aligned}$$

Now pick $t_0$ so large that

B.30 $$\begin{aligned} \frac{{\bar{G}}(s)}{s}> 1 - \mu - \xi \text{ for } s\le \max (1,\theta /\theta _0){\bar{G}}_{t_0-1}(1), \end{aligned}$$

where *ξ>0* is a small number, to be chosen below. From this it follows that the values of $S_1(t_0;\theta _0,\theta )$ and $S_1(t_0;\theta ,\theta )$ are between two geometric series with ratios $1-\mu -\xi$ and *1-μ*, as

B.31 $$\begin{aligned} \begin{array}{rcl} S_1(t_0;\theta _0,\theta ), S_1(t_0;\theta ,\theta ) & \in & \left( 2\theta _0 \frac{(\theta /\theta _0) {\bar{G}}_{t_0-1}(1)(1-\mu -\xi )}{1 - (1-\mu -\xi )}, 2\theta _0 \frac{(\theta /\theta _0) {\bar{G}}_{t_0-1}(1)(1-\mu )}{1 - (1-\mu )}\right) \\ & =& \left( 2\theta {\bar{G}}_{t_0-1}(1) \frac{1-\mu -\xi }{\mu +\xi }, 2\theta {\bar{G}}_{t_0-1}(1) \frac{1-\mu }{\mu }\right) . \end{array} \end{aligned}$$

Since ${\bar{G}}_k(1)$ decreases with *k*, we also have that

B.32 $$\begin{aligned} S_2(t_0;\theta ) \ge 2\theta t_0 {\bar{G}}_{t_0-1}(1). \end{aligned}$$

Inserting (B.31)-(B.32) into (B.29), it suffices to choose *ξ* so small that

B.33 $$\begin{aligned} \eta = \frac{(1-\mu )/\mu - (1-\mu -\xi )/(\mu +\xi )}{(1-\mu -\xi )/(\mu +\xi ) + t_0} = \frac{(\mu + \xi )(1-\mu )-\mu (1-\mu -\xi )}{\mu (1-\mu -\xi )+t_0 \mu (\mu +\xi )}. \end{aligned}$$

Assuming that *μ>0* and *ξ>0* are both small, we can replace the terms *1-μ* and $1-\mu -\xi$ on the right-hand side of (B.33) by 1. This gives a simplified condition

B.34 $$\begin{aligned} \frac{\xi }{\mu + t_0\mu (\mu +\xi )} = \eta \end{aligned}$$

on *ξ*. Solving for *ξ* we find that

B.35 $$\begin{aligned} \xi = \frac{\eta \mu (1+t_0\mu )}{1-\eta \mu t_0}. \end{aligned}$$

Inserting (B.35) into (B.30), it follows that $t_0$ should be chosen so large that

B.36 $$\begin{aligned} \frac{{\bar{G}}(s)}{s}> 1 - \mu - \frac{\eta \mu (1+t_0\mu )}{1-\eta \mu t_0} \text{ for } s\le \max (1,\theta /\theta _0){\bar{G}}_{t_0-1}(1). \end{aligned}$$

By a second order Taylor expansion of ${\bar{G}}(s)$ around *s=0*, we find that

B.37 $$\begin{aligned} \frac{{\bar{G}}(s)}{s} \approx 1-\mu - \frac{s}{2}|{\bar{G}}^{\prime \prime }(0)| \end{aligned}$$

for small *s>0*, since ${\bar{G}}^{\prime \prime }(0)=-(1-\mu )^2/d< 0$. Comparing (B.37) with (B.36), it follows that $t_0$ approximately must satisfy

B.38 $$\begin{aligned} f(t_0) = \frac{(1-\eta \mu t_0){\bar{G}}_{t_0-1}(1)}{1+t_0\mu } \le \frac{2\eta \mu }{\max (1,\theta /\theta _0)|{\bar{G}}^{\prime \prime }(0)|} \approx \frac{2d\eta }{\max (1,\theta /\theta _0)} \cdot \mu , \end{aligned}$$

where in the last step we assumed that *μ>0* is small. Note that $f(t_0)$ is a strictly decreasing function of $t_0$ with $f((\eta \mu )^{-1})=0$, whereas ${\bar{G}}_{t_0-1}(1)\ge d[1-\exp (-m)] (1-\mu )^{t_0-2}\approx d[1-\exp (-m)]\exp [-\mu (t_0-2)]$ for $t_0\ge 2$. Regarding $\eta ,d,\theta /\theta _0>0$ as fixed, and solving the inequality in (B.38) with respect to $t_0$, for each small *μ>0*, we deduce $t_0=O(\mu ^{-1})$, as was to be proved.

## C Grid-based Approximation of Allele Frequency Spectrum

In this appendix we analyze the grid-based approximation $\hat{\boldsymbol{ F}}(t)$ of the EAFS, defined in (97). Recall that in order to define $\hat{\boldsymbol{ F}}(t)$ we replace the set ${{{\mathcal {X}}}}(t)$ of *2N(t)+1* allele frequencies of generation *t*, with a coarser grid $\hat{{{{\mathcal {X}}}}}(t)$ of *K(t)≤ 2N(t)+1* allele frequencies, defined in (96). To specify more precisely this grid $\hat{{{{\mathcal {X}}}}}(t)$ of allele frequencies, assume that $2N(t)+1=2K_0(t)+qK_1(t)$, with *q* an odd integer and $K_0(t)$ an integer much smaller than *N*(*t*). Then put $K(t)=2K_0(t)+K_1(t)$. It is assumed that each one of the *K*(*t*) intervals that ${{{\mathcal {X}}}}(t)$ is divided into, contains an odd number of adjacent allele frequences from ${{{\mathcal {X}}}}(t)$. The first and last $K_0(t)$ intervals contain one single allele frequency, whereas the $K_1(t)$ middle intervals have a larger size *q*.

The matrix recursion (99) for computing $\hat{\boldsymbol{ F}}(t+1)$ from $\hat{\boldsymbol{ F}}(t)$, is a grid-based approximation of the corresponding matrix recursion (38) of the EAFS, between generations *t* and *t+1*. For ease of notation we drop generation number of the grid-based transition matrix $\hat{\boldsymbol{ P}}=\hat{\boldsymbol{ P}}(t)$ in (100) and rewrite (99) as

C.1 $$\begin{aligned} \hat{\boldsymbol{ F}}(t+1) = \hat{\boldsymbol{ F}}(t)\hat{\boldsymbol{ P}}+ 2\theta (t+1)\hat{\boldsymbol{ \delta }}_{\varepsilon (t+1)}. \end{aligned}$$

Our goal is to find explicit expressions for $\hat{\boldsymbol{ P}}=\hat{\boldsymbol{ P}}(t)$. Since the transition matrix $\boldsymbol{ P}=\boldsymbol{ P}(t)$ of the Markov chain *X(· )* is defined differently for monoecious and dioecious populations, and $\hat{\boldsymbol{ P}}$ is computed from $\boldsymbol{ P}$, the monoecious and dioecious cases are treated separately in Appendices C.1 and C.2.

### C. 1 Monoecious Population

Recall from (A.3) that the transition matrix of the Markov process *X(· )* for a monoecious population, between generations *t* and *t+1*, takes the form

C.2 $$\begin{aligned} \boldsymbol{ P}= \boldsymbol{ P}_1 \boldsymbol{ P}_{23}, \end{aligned}$$

where the elements of $\boldsymbol{ P}_1=(P_{1xz};\, x\in {{{\mathcal {X}}}}(t),z\in {{{\mathcal {Z}}}}(t))$ and $\boldsymbol{ P}_{23}=(P_{23zy};\, z\in {{{\mathcal {Z}}}}(t),y\in {{{\mathcal {X}}}}(t+1))$ are defined in (A.4) and (A.5), respectively. Thus we need to find a grid-based analogoue

C.3 $$\begin{aligned} \hat{\boldsymbol{ P}}= \hat{\boldsymbol{ P}}_1 \hat{\boldsymbol{ P}}_{23} \end{aligned}$$

of $\boldsymbol{ P}$, based on formula (101), with $\hat{\boldsymbol{ P}}_1$ and $\hat{\boldsymbol{ P}}_{23}$ the grid-based versions of $\boldsymbol{ P}_1$ and $\boldsymbol{ P}_{23}$. To this end, write the elements of $\hat{\boldsymbol{ P}}$ as

C.4 $$\begin{aligned} {\hat{P}}_{ab} = \sum _{c\in \hat{{{{\mathcal {Z}}}}}(t)} {\hat{P}}_{1ac}{\hat{P}}_{23,cb} \end{aligned}$$

for all $a\in \hat{{{{\mathcal {X}}}}}(t)$ and $b\in \hat{{{{\mathcal {X}}}}}(t+1)$, with $\hat{{{{\mathcal {Z}}}}}(t)$ a grid-based approximation of ${{{\mathcal {Z}}}}(t)$. Assuming that $|{{{\mathcal {Z}}}}(t)|=2N_{e}(t)+1=2K_{0e}(t)+q_e K_{1e}(t)$ for some positive integers $K_{0e}(t)$ and $K_{1e}(t)$, and some positive, odd integer $q_e$, this grid has size $|\hat{{{{\mathcal {Z}}}}}(t)|=K_e(t)=2K_{0e}(t)+K_{1e}(t)$, corresponding to $K_{0e}(t)$ sets of single allele frequencies close to 0, $K_{0e}(t)$ sets of single allele frequencies close to 1, and $K_{1e}(t)$ sets of adjacent, intermediate allele frequences, each of size $q_e$.

In the following two subsections we will introduce two different ways of approximating the transition probabilities ${\hat{P}}_{1ac}$ and ${\hat{P}}_{23,cb}$ that appear in (C.4).

#### C.1.1 Approximate Linear Combination of Exact Transition Probabilities

In this subsection we define the elements of matrices ${\hat{P}}_1$ and ${\hat{P}}_{23}$ as approximations

C.5 $$\begin{aligned} \begin{array}{rcl} {\hat{P}}_{1ac} & \approx & \sum _{x\in I_a}\sum _{z\in L_c} P_{1xz} /|I_a|,\\ {\hat{P}}_{23,cb} & \approx & \sum _{z\in L_c}\sum _{y\in J_b} P_{23,zy}/|L_c| \end{array} \end{aligned}$$

of linear combinations of the exact transition probabilities $P_{1xz}$ and $P_{23,zy}$, with $L_c\subset {{{\mathcal {Z}}}}(t)$ an interval that surrounds $c\in \hat{{{{\mathcal {Z}}}}}(t)$ and hence is part of the approximating grid $\hat{{{{\mathcal {Z}}}}}(t)$ of ${{{\mathcal {Z}}}}(t)$. In order to define ${\hat{P}}_{1ac}$ and ${\hat{P}}_{23,cb}$ in more detail, we will make use of the fact that *a*, *c*, and *b* are the mid points of $I_a=\{a_{-1},\ldots ,a_1\}$, $L_c=\{c_{-1},\ldots ,c_1\}$, and $J_b=\{b_{-1},\ldots ,b_1\}$, respectively. Let $|I_a|=a_1-a_{-1}+1$, $|L_c|=c_1-c_{-1}+1$ and $|J_b|=b_1-b_{-1}+1$ refer to the odd number of elements of $I_a$, $L_c$, and $J_b$. Since the right-hand sides of (C.5) involve summation over $|I_a||L_c|$ and $|L_c||J_b|$ terms, it is computationally infeasible to evaluate these sums when $|I_a|$, $|L_c|$, and $|J_b|$ are large. For this reason we will define ${\hat{P}}_{1ac}$ and ${\hat{P}}_{23,cb}$ in such a way the sums in (C.5) are taken over fewer terms.

When all individuals are breeders ($N(t)=N_e(t)$), it follows from (A.1) and (A.4) that *X(t)=Z(t)* and $P_{1xz}=1(x=z)$. It is natural then to use the same grids $\hat{{{{\mathcal {X}}}}}(t)=\hat{{{{\mathcal {Z}}}}}(t)$ for *X*(*t*) and *Z*(*t*), so that the right hand side of the upper part of (C.5) simplifies to

$$\begin{aligned} {\hat{P}}_{1ac} = 1(a=c). \end{aligned}$$

In the general case, we have to find computationally tractable approximations of (C.5). The simplest definition of ${\hat{P}}_{1ab}$ and ${\hat{P}}_{23,cb}$ is obtained by replacing $P_{1xz}$ and $P_{23,zy}$, on the right-hand sides of (C.5), by $P_{1ac}$ and $P_{23,cb}$, respectively. Thus we replace the transition probability $P_{1xz}$, for each pair $x\in I_a$, $z\in L_c$, by the transition probability $P_{1ab}$ that corresponds to the mid points of $I_a$ and $L_c$, and similarly we replace each $P_{23,zy}$ by a probability $P_{23,cb}$ that corresponds to a transition between the mid points of $L_c$ and $J_b$. This yields

C.6 $$\begin{aligned} \begin{array}{rcl} {\hat{P}}_{1ac} & = & |L_c| P_{1ac},\\ {\hat{P}}_{23,cb} & = & |J_b| P_{23,cb}. \end{array} \end{aligned}$$

A more refined approximation of the right-hand side of the upper part of (C.5) is obtained by replacing $P_{1,xz}$, for each pair $x\in I_a$ and $z\in L_c$, by a linear interpolation of the nine elements of $P_{1}$ that are defined by combining the three end/mid points of $I_a$ and $L_c$, in all $9=3^2$ possible ways. Similarly, an improved approximation of the right-hand side of the lower part of (C.5) is defined by replacing $P_{23,zy}$, for each pair $z\in L_c$ and $y\in J_b$, by a linear interpolation of the nine elements of $P_{23}$ that combine the three end/mid points of $L_c$ and $J_b$. After some computations (see below), this yields

C.7 $$\begin{aligned} \begin{array}{rcl} {\hat{P}}_{1ac} & = & |L_c| \sum _{\alpha \in \Gamma } \sum _{\gamma \in \Gamma } w_{\alpha |I_a|} w_{\gamma |L_c|}P_{1a_\alpha c_\gamma },\\ {\hat{P}}_{23,cb} & = & |J_b| \sum _{\gamma \in \Gamma } \sum _{\beta \in \Gamma } w_{\gamma |L_c|} w_{\beta |J_b|}P_{23,c_\gamma b_\beta }, \end{array} \end{aligned}$$

where

C.8 $$\begin{aligned} \Gamma = \{-1,0,1\}, \end{aligned}$$

$a_{-1}$, $a_0=a$, $a_1$ refer to the lower end point, mid point and upper end point of $I_a$, and similarly $b_{-1}$, $b_0=b$, $b_1$, and $c_{-1}$, $c_0=c$, $c_1$, denote lower end point, mid point and upper end point of $J_b$ and $L_c$, respectively. The two sums in (C.7) are weighted averages of 9 transition probabilities, with weights obtained from

C.9 $$\begin{aligned} \begin{array}{rcl} w_{0h} & =& (h-1)/(2h) + 1(h=1),\\ w_{-1,h} = w_{1h} & =& (1-w_{0h})/2 \end{array} \end{aligned}$$

for any odd number *h*. For instance, when $|I_a|$ and $|L_c|$ are large, the weights assigned to a term of the upper part of (C.7) are approximately $(1/2)^2=1/4$, *(1/2)· (1/4) = 1/8*, and $(1/4)^2=1/16$ for pairs $a_\alpha$, $c_\gamma$ such that two, one or none of its elements is a mid point of $I_a$ or $L_c$. Note also that $a_{-1}=a_0=a_1=a$ when $|I_a|=1$, and in this case summation over *α*, as well as the term $w_{\alpha |I_a|}$, can be skipped in (C.7). Similarly, summation over *β* and *γ* can be skipped in (C.7) when $|J_b|=1$ and $|L_c|=1$, respectively.

We will only derive the upper part of (C.7), since the lower part is motivated analogously. For each $x\in I_a$ and $z\in L_c$, we replace $P_{1xz}$ by a linear interpolation

C.10 $$\begin{aligned} {\hat{P}}_{1,xz} = \sum _{\alpha \in \Gamma } \sum _{\gamma \in \Gamma } v_{x \alpha }^{a|I_a|} v_{z \gamma }^{c|L_c|}P_{1,a_\alpha c_\gamma } \end{aligned}$$

of 9 different $P_{1,a_\alpha c_\gamma }$, whose second and third indices are end/mid points of $I_a$ and $L_c$, respectively. The weights of this linear interpolation depend on *x* and *z*, with

$$\begin{aligned} \begin{array}{rcl} v_{x,-1}^{a1} & =& 0,\\ v_{x0}^{a1} & =& 1,\\ v_{x1}^{a1} & =& 0 \end{array} \end{aligned}$$

when *h=1*,

$$\begin{aligned} \begin{array}{rcl} v_{x,-1}^{ah} & =& 1(x\le a) (a-x)/(a-(h-1)/2),\\ v_{x0}^{ah} & =& \min [(x-a+(h-1)/2)/((h-1)/2),(a+(h-1)/2-x)/((h-1)/2)],\\ v_{x1}^{ah} & =& 1(x\ge a) (x-a)/((h-1)/2) \end{array} \end{aligned}$$

for any odd number *h>1*, and with the weights $v_{z\gamma }^{ch}$ defined analogously. Note that $v_{x\alpha }^{ah}$ and $v_{z\gamma }^{ch}$ account for the fact that $a_{-1}=a=a_1$ when $h=|I_a|=1$, and $c_{-1}=c=c_1$ when $h=|L_c|=1$, so that no transition probability is duplicated in the summation (C.10). Replacing $P_{1,xz}$ by (C.10), on the right-hand side of the upper part of (C.5), after some computations (which involves switching the order of summation between *x*, *z* and $\alpha ,\gamma$) we obtain the right-hand side of the upper part of (C.7).

#### C.1.2 Normal Approximations

Define $m_{Zx}=E[Z(t)|X(t)=x]$ and $\sigma _{Zx}^2 = \mathrm{Var}[Z(t)|X(t)=x]$ as in (A.7) and (A.8), respectively. Let also ${\bar{L}}_c = [c_{-1}-1/(4N_e(t)),c_1+1/(4N_e(t))]$ be an interval that surrounds $c\in \hat{{{{\mathcal {Z}}}}}(t)$, containing all elements of $L_c=\{c_{-1},\ldots ,c_1\}$ defined in the paragraph below (C.5). In this subsection we use a normal approximation

C.11 $$\begin{aligned} {\hat{P}}_{1ac} \approx \sum _{x\in I_a} \Phi \left( ({\bar{L}}_c-m_{Zx})/\sigma _{Zx}\right) /|I_a| \end{aligned}$$

in order to define the transition probability ${\hat{P}}_{1ac}$ that appears in (C.4), with *Φ* the standard normal cumulative distribution function. The other transition probability ${\hat{P}}_{23,cb}$ of (C.4) is analogously based on a normal approximation

C.12 $$\begin{aligned} {\hat{P}}_{23,cb} \approx \sum _{z\in L_c} \Phi \left( ({\bar{J}}_b-m_{Xz})/\sigma _{Xz}\right) /|L_c|, \end{aligned}$$

with $m_{Xz}=E[X(t+1)|Z(t)=z]$ and $\sigma _{Xz}^2 = \mathrm{Var}[X(t+1)|Z(t)=z]$ defined as in (A.9) and (A.10), respectively, ${\bar{J}}_b = [b_{-1}-1/(4N(t+1)),b_1+1/(4N(t+1))]$ an interval that surrounds $b\in \hat{{{{\mathcal {X}}}}}(t+1)$, containing all elements of $J_b=\{b_{-1},\ldots ,b_1\}$ defined below (C.5).

When the number of elements of $I_a$ and $L_c$ are large, it is necessary to approximate the right-hand sides of (C.11) and (C.12). The simplest approximation is to replace *x* and *z* in the two sums of (C.11) and (C.12) by *a* and *c*, respectively, giving

C.13 $$\begin{aligned} \begin{array}{rcl} {\hat{P}}_{1ac} & =& \Phi \left( ({\bar{L}}_c-m_{Za})/\sigma _{Za}\right) ,\\ {\hat{P}}_{23,cb} & =& \Phi \left( ({\bar{J}}_b-m_{Xc})/\sigma _{Xc}\right) . \end{array} \end{aligned}$$

Slightly more accurate approximations

C.14 $$\begin{aligned} \begin{array}{rcl} {\hat{P}}_{1ac} & =& \sum _{\alpha \in \Gamma } w_{\alpha |I_a|}\Phi \left( ({\bar{L}}_c-m_{Za_\alpha })/\sigma _{Za_\alpha }\right) ,\\ {\hat{P}}_{23,cb} & =& \sum _{\gamma \in \Gamma } w_{\gamma |L_c|}\Phi \left( ({\bar{J}}_b-m_{Xc_\gamma })/\sigma _{Xc_\gamma }\right) \end{array} \end{aligned}$$

are obtained from a linear interpolation approximation of the terms of the two sums of the right-hand sides of (C.11) and (C.12), with *Γ*, $w_{\alpha h}$ and $w_{\gamma h}$ defined in (C.8) and (C.9).

### C. 2 Dioecious Population

The transition matrix (A.18) of the Markov process *X(· )* for a dioecious population, between generations *t* and *t+1*, can be written as

C.15 $$\begin{aligned} \boldsymbol{ P}= \boldsymbol{ P}_1 \boldsymbol{ P}_{23} \boldsymbol{ P}_3, \end{aligned}$$

where the elemens of $\boldsymbol{ P}_1=(P_{1x{\boldsymbol{z}}})$, $\boldsymbol{ P}_{23}=(P_{23{\boldsymbol{z}}{\boldsymbol{y}}})$, and $\boldsymbol{ P}_3=(P_{3{\boldsymbol{y}}y})$ are defined in (A.19) and (A.20), and (A.21), respectively. Thus we need to define a grid-based analogoue

C.16 $$\begin{aligned} \hat{\boldsymbol{ P}}= \hat{\boldsymbol{ P}}_1 \hat{\boldsymbol{ P}}_{23} \hat{\boldsymbol{ P}}_3 \end{aligned}$$

of this recursion, based on formula (101), with $\hat{\boldsymbol{ P}}_1=({\hat{P}}_{1ac})$, $\hat{\boldsymbol{ P}}_{23}=({\hat{P}}_{23cd})$, and $\hat{\boldsymbol{ P}}_3=({\hat{P}}_{3db})$ the grid-based approximations of $\boldsymbol{ P}_1$, $\boldsymbol{ P}_{23}$, and $\boldsymbol{ P}_3$. The elements of the matrix $\hat{\boldsymbol{ P}}$ are defined as

C.17 $$\begin{aligned} {\hat{P}}_{ab} = \sum _{c\in \hat{{{{\mathcal {Z}}}}}(t),d\in \hat{{{{\mathcal {Y}}}}}(t)} {\hat{P}}_{1ac}{\hat{P}}_{23,cd}{\hat{P}}_{3db} \end{aligned}$$

for all $a\in \hat{{{{\mathcal {X}}}}}(t)$ and $b\in \hat{{{{\mathcal {X}}}}}(t+1)$, where $\hat{{{{\mathcal {Z}}}}}(t)$ and $\hat{{{{\mathcal {Y}}}}}(t)$ are grid-based versions of the sets ${{{\mathcal {Z}}}}(t)$ and ${{{\mathcal {Y}}}}(t)$, defined in (A.16) and (A.17). For simplicity, in order to define the elements of the transition matrices $\hat{\boldsymbol{ P}}_1$, $\hat{\boldsymbol{ P}}_{23}$, and $\hat{\boldsymbol{ P}}_3$, on the right-hand side of (C.17), we only consider approximations of linear combinations of exact transition probabilities, as in Section C.1.1. In more detail, the elements of the right-hand side of (C.17) will be chosen so that

C.18 $$\begin{aligned} \begin{array}{rcl} {\hat{P}}_{1ac} & \approx & \sum _{x\in I_a}\sum _{{\boldsymbol{z}}\in L_c} P_{1,x{\boldsymbol{z}}} / |I_a|,\\ {\hat{P}}_{23,cd} & \approx & \sum _{{\boldsymbol{z}}\in L_c}\sum _{{\boldsymbol{y}}\in K_d} P_{23,{\boldsymbol{z}}{\boldsymbol{y}}} / |L_c|,\\ {\hat{P}}_{3db} & \approx & \sum _{{\boldsymbol{y}}\in K_d}\sum _{y\in J_b} P_{3{\boldsymbol{y}}y} / |K_d|, \end{array} \end{aligned}$$

where $I_a$ is the interval of ${{{\mathcal {X}}}}(t)$ that surrounds *a*, $L_c = L_{c_m}\times L_{c_f}$ the rectangle of ${{{\mathcal {Z}}}}(t)$ that surrounds $c=(c_m,c_f)$, $K_d = K_{d_m}\times K_{d_f}$ the rectangle of ${{{\mathcal {Y}}}}(t)$ that surrounds $d=(d_m,d_f)$, and $J_b$ the interval of ${{{\mathcal {X}}}}(t+1)$ that surrounds *b*. Put $I_a=\{a_{-1},\ldots ,a_1\}$, $J_b=\{b_{-1},\ldots ,b_1\}$, $L_{c_m} = \{c_{m,-1},\ldots ,c_{m1}\}$, $L_{c_f} = \{c_{f,-1},\ldots ,c_{f1}\}$, $K_{d_m}=\{d_{m,-1},\ldots ,d_{m1}\}$ and $K_{d_f}=\{d_{f,-1},\ldots ,d_{f1}\}$. We will assume that the number of elements $|I_a|$, $|J_b|$, $|L_{c_m}|$, $|L_{c_f}|$, $|K_{d_m}|$, and $|K_{d_f}|$ of these intervals are all odd. Note also that $|L_c|=|L_{c_m}||L_{c_f}|$ and $|K_d| = |K_{d_f}||K_{d_m}|$.

The simplest definition of ${\hat{P}}_{1ac}$ and ${\hat{P}}_{23,cd}$ is obtained by replacing the transition probabilities $P_{1,x{\boldsymbol{z}}}$ and $P_{23,{\boldsymbol{z}}{\boldsymbol{y}}}$ in the sums of the right-hand sides of (C.18) with transition probabilities $P_{1ac}$ and $P_{23,cd}$ between elements whose coordinates are mid points of the intervals or rectangles of each grid. For ${\hat{P}}_{3db}$ we can make use of the special form of the transition probability $P_{3{\boldsymbol{y}}y}$ in (A.21) and replace $P_{3{\boldsymbol{y}}y}$ by a transition probability $P_{3dy}$ for which only the first index is a mid point of a rectangle of a grid. Analogously to (C.6), this yields

C.19 $$\begin{aligned} \begin{array}{rcl} {\hat{P}}_{1ac} & =& |L_c| P_{1,ac},\\ {\hat{P}}_{23,cd} & = & |K_d| P_{23,cd},\\ {\hat{P}}_{3db} & =& 1((d_m+d_l)/2 \in J_b). \end{array} \end{aligned}$$

A more refined definition of ${\hat{P}}_{1ac}$ and ${\hat{P}}_{23,cd}$ is obtained by replacing each transition probability $P_{1,x{\boldsymbol{z}}}$ and $P_{23,{\boldsymbol{z}}{\boldsymbol{y}}}$ in the sums of the right-hand sides of (C.18) by a linear interpolation of transition probabillities between states that are perimeter or mid points of the intervals or rectangles that *x*, $\boldsymbol{ z}$ and $\boldsymbol{ y}$ belong to. Simlarly, a refined definition of $P_{3db}$ is derived by replacing $P_{3{\boldsymbol{y}}y}$ in (C.18) by a linear interpolation of transition probabilities between states that are perimeter or mid points of the rectangle that $\boldsymbol{ y}$ belongs to, and *y*. By a similar argument that led to (C.6), we find after some computations that

C.20 $$\begin{aligned} \begin{array}{rcl} {\hat{P}}_{ac} & =& |L_c| \sum _{\alpha \in \Gamma } \sum _{\gamma \in \Gamma ^2} w_{\alpha |I_a|}w_{\gamma _1|L_{c_m}|}w_{\gamma _2|L_{c_f}|}P_{1,a_\alpha c_{\gamma }},\\ {\hat{P}}_{23,cd} & =& |K_d|\sum _{\gamma \in \Gamma ^2} \sum _{\delta \in \Gamma ^2} w_{\gamma _1|L_{c_m}|}w_{\gamma _2|L_{c_f}|} w_{\delta _1|K_{d_m}|} w_{\delta _2|K_{d_f}|} P_{23,c_{\gamma },d_{\delta }},\\ {\hat{P}}_{3db} & =& \sum _{\delta \in \Gamma ^2} w_{\delta _1|K_{d_m}|} w_{\delta _2|K_{d_f}|} 1((d_{m,\delta _1}+d_{f,\delta _2})/2 \in J_b), \end{array} \end{aligned}$$

where *Γ* is defined in (C.8), $\gamma =(\gamma _1,\gamma _2)$, $\delta =(\delta _1,\delta _2)$, whereas $w_{-1h}$, $w_{0h}$ and $w_{1h}$ are weights defined in (C.9) for any odd number *h*. In the sums of (C.20), the mid points of the intervals $I_a$, $L_{c_m}$, $L_{c_f}$, $K_{d_m}$, $K_{d_f}$, and $J_b$ are denoted as $a=a_0$, $c_m=c_{m0}$, $c_f=c_{f0}$, $d_m=d_{m0}$, $d_f=d_{f0}$, and $b=b_0$, respectively. Then we introduced the notation

$$\begin{aligned} \begin{array}{rcl} c_{\gamma } & =& (c_{m\gamma _1},c_{f\gamma _2}),\\ d_{\delta } & =& (d_{m\delta _1},d_{f\delta _2}), \end{array} \end{aligned}$$

so that $c_{\gamma }$ and $d_{\delta }$ range over all nine vectors whose coordinates are end or mid points of $L_{c_m},L_{c_f}$ and $K_{d_m},K_{d_f}$, respectively, when *γ* and *δ* vary over $\Gamma ^2$.

### C.3 Normalization for Expected Total Allele Frequency 1

A natural requirement on $\hat{\boldsymbol{ F}}(t)$, the grid-based EAFS, is that the expected total allele frequency satisfies

C.21 $$\begin{aligned} \sum _{x\in \hat{{{{\mathcal {X}}}}}(t)} x{\hat{F}}_x(t) = \hat{\boldsymbol{ F}}(t)\hat{\boldsymbol{ g}}_{\mathrm{id}} = 1, \end{aligned}$$

where $g_{\mathrm{id}}(x)=x$ is the identity function that was introduced in Example 2, whereas $\hat{\boldsymbol{ g}}_{\mathrm{id}}=(g_{\mathrm{id}}(x); \, x\in \hat{{{{\mathcal {X}}}}}(t))^\prime$ is the corresponding column vector of length $|\hat{{{{\mathcal {X}}}}}(t)|$. Recall that the grid-based approximation $\hat{\boldsymbol{ P}}=\hat{\boldsymbol{ P}}(t)$ of the transition matrix $\boldsymbol{ P}=\boldsymbol{ P}(t)$ of the allele frequency Markov chain *X(· )* is needed in (C.1) for the time recursion of the grid-based EAFS $\hat{\boldsymbol{ F}}(t)$. The matrix $\hat{\boldsymbol{ P}}$ was derived separately for monoecious and dioecious populations in Sections C.1 and C.2. In either case the idenity

C.22 $$\begin{aligned} \hat{\boldsymbol{ P}}\hat{\boldsymbol{ g}}_{\mathrm{id}} = (1-\mu )\hat{\boldsymbol{ P}} \end{aligned}$$

might not hold. In analogy with (46)-(47) we deduce that if (C.22) is not satisfied, then (C.21) will typically fail at time *t+1*, even if (C.21) holds at time *t*. For this reason we modify (C.1) to

C.23 $$\begin{aligned} {\hat{F}}_x(t+1) = \left\{ \begin{array}{ll} (\hat{\boldsymbol{ F}}(t)\hat{\boldsymbol{ P}})_0, & x=0,\\ \frac{1-\mu }{\sum _{y\in \hat{{{{\mathcal {X}}}}}(t+1)} y (\hat{{\boldsymbol{F}}}(t)\hat{{\boldsymbol{P}}})_y} (\hat{\boldsymbol{ F}}(t)\hat{\boldsymbol{ P}})_x + 2\theta (t+1)1(x=\varepsilon (t+1)), & x\in \hat{{{{\mathcal {X}}}}}(t+1)\setminus \{0\}. \end{array}\right. \end{aligned}$$

Since $2\theta (t+1)\varepsilon (t+1)=\mu$, this modification of ${\hat{F}}_x$ will guarantee that (C.21) is satisfied at time *t+1*, given that this equation holds at time *t*.

### C.4 Numerical Examples

In this section we illustrate the numerical accuracy of the grid-based approximation of the expected allele frequency spectrum (EAFS) as well as some summary statistics of the EAFS. We consider two rather small monoecious populations of constant census sizes *N=20* and *N=30*, and study the EAFS during 11 generations (*t=0,1,… ,10*). Both populations are in genetic drift-mutation equilibrium during this period, so that for both populations the EAFS in (57), for positive allele frequencies, equals the equilibrium distributon given by (59) for *t=0,1,… ,10*. This equilibrium EAFS will be used as a benchmark.

We consider three scenarios, of which Scenario 1 is our benchmark that generates the exact equilibrium EAFS for *t=0,1,… ,10* for positive allele frequencies, and the exact expected number of lost alleles for allele frequency 0. The other two scenarios give rise to approximations (97) of this EAFS. Scenarios 1 and 2 have a full grid of all *2N+1* allele frequencies whereas Scenario 3 utilizes a sparser grid of allele frequencies. Additionally, Scenarios 2 and 3 make use of normal approximations (C.14) of transition probabilities. For the approximate Scenarios 2-3, the EAFS is always normalized to have a total allele frequency of 1, according to (C.21) and (C.23).

The numerical results for the two models are shown in Tables 4 and 5, respectively. Note in particular that the approximate EAFS in (97) does not start in drift-mutation equilibrium for positive allele frequencies, for the two approximate Scenarios 2-3. The reason is that the approximate EAFSs need to have their spectra normalized according to (C.23), so that (C.21) holds. This implies that the plug-in approximation of (59) (where $\boldsymbol{ P}$ is replaced by $\hat{\boldsymbol{ P}}$) will not generate the approximate equilibrium EAFS at *t=0*. Instead, one has to iterate (C.1) until convergence in order to attain this approximate equilibrium EAFS for the two approximate scenarios. Because of the high mutation rate *μ =0.2*, ten generations is a sufficiently large burn-in time to almost attain convergence. For this reason the numerical values for *t=10* represent the approximate equilibrium EAFS for positive allele frequencies fairly well, for Scenarios 2 and 3.

Not surprisingly, we see that the normal approximations of Scenarios 2 and 3 work better for the larger population of Table 5 than for the smaller population of Table 4, regardless of whether a full or sparse grid of allele frequencies is used.

**Table 4:** Numerical values of various quantities for a monoecious population with constant census size *N=20*, effective size $N_e=10$, and mutation rate *μ =0.2*, evaluated at generations *t=0* and *t=10*. These quantities are the expected number $F_x(t)$ of alleles with *n* copies and frequency *x=n/(2N)*, the expected total number of (present or historical) alleles $E[A_{n\mathrm{tot}}(t)]$, the expected total number of (present) alleles $E[A_{n}(t)]$, the expected gene diversity *E*[*H*(*t*)], and the expected triplet gene diversity $E[H_3(t)]$

	Scenario 1 (exact)		Scenario 2		Scenario 3	
	Full grid, no NA		Full grid, NA		Sparse grid, NA	
Quantity	*t=0*	*t=10*	*t=0*	*t=10*	*t=0*	*t=10*
$F_{0}(t)$	0.0000	80.0000	0.0000	80.5209	0.0000	77.9404
$F_{1/(2N)}(t)$	10.8799	10.8799	11.1008	10.9079	10.2696	10.7588
$F_{2/(2N)}(t)$	2.6488	2.6488	2.6858	2.6743	2.4852	2.5242
$F_{3/(2N)}(t)$	1.7925	1.7925	1.8111	1.8089	1.6769	1.6921
$F_{4/(2N)}(t)$	1.0868	1.0868	1.0927	1.0953	1.0124	1.0117
$F_{5/(2N)}(t)$	0.6668	0.6668	0.6659	0.6705	0.6187	0.6115
$E[A_{n\tiny \text{ tot }}(t)]$	18.4027	98.4027	18.6431	98.9856	17.4412	95.8619
$E[A_{n}(t)]$	18.4027	18.4027	18.6431	18.4647	17.4412	17.9215
*E*[*H*(*t*)]	0.8954	0.8954	0.8973	0.8962	0.8761	0.8800
$E[H_3(t)]$	0.7233	0.7233	0.7278	0.7251	0.6847	0.6935

It is assumed that the population is in drift-mutation equilibrium. All quantities are functions of the Expected Allele Frequency Spectrum (EAFS). Only Scenario 1 is based on exact compuation of the EAFS. The other two scenarios either make use of a sparse grid of allele frequencies and/or normal approximations (NAs) of transition probabilities. The number of grid points (allele frequencies *x* and *z*) of the full grid are $|{{{\mathcal {X}}}}|=2N+1=41$ and $|{{{\mathcal {Z}}}}|=2N_e+1=21$ before and after Step 1 of the reproduction cycle. The corresponding numbers for the sparse grid are $|\hat{{{{\mathcal {X}}}}}|=21 = 2\times 8 + 5$ ($K_0(t)=8$ allele frequency intervals of unit length, close to 0 and 1, respectively, and $K_1(t)=5$ allele frequency intervals of length *q=5* for intermediate allele frequencies) and $|\hat{{{{\mathcal {Z}}}}}|=15=2\times 6 + 3$ ($K_{0e}(t)=6$ allele frequency intervals of unit length, close to 0 and 1, respectively, and $K_{1e}(t)=3$ allele frequency intervals of length $q_e=3$ for intermediate allele frequencies). Note that the expected number $F_0(t)$ of lost alleles and the expected number $E[A_{n \mathrm{tot}}(t)]=F_0(t)+E[A_n(t)]$ of lost or present alleles do not converge as $t\rightarrow \infty$, since lost alleles accumulate over time

**Table 5:** lost alleles and the expected numbernstant census size *N=30*, effective size $N_e=15$, and mutation rate *μ =0.2*, evaluated at generations *t=0* and *t=10*. These quantities and the three scenarios are the same as in Table 4

	Scenario 1 (exact)		Scenario 2		Scenario 3	
	Full grid, no NA		Full grid, NA		Sparse grid, NA	
Quantity	*t=0*	*t=10*	*t=0*	*t=10*	*t=0*	*t=10*
$F_{0}(t)$	0.0000	120.0000	0.0000	120.7673	0.0000	119.9710
$F_{1/(2N)}(t)$	16.2836	16.2836	16.6121	16.3244	16.3679	16.2777
$F_{2/(2N)}(t)$	3.9147	3.9147	3.9695	3.9520	3.9113	3.9053
$F_{3/(2N)}(t)$	2.6455	2.6455	2.6747	2.6700	2.6355	2.6337
$F_{4/(2N)}(t)$	1.6042	1.6042	1.6142	1.6176	1.5906	1.5914
$F_{5/(2N)}(t)$	0.9843	0.9843	0.9843	0.9906	0.9700	0.9718
$E[A_{n\tiny \text{ tot }}(t)]$	27.4467	147.4467	27.8087	148.3078	27.4430	147.3284
$E[A_{n}(t)]$	27.4467	27.4467	27.8087	27.5405	27.4430	27.3574
*E*[*H*(*t*)]	0.9283	0.9283	0.9297	0.9289	0.9257	0.9255
$E[H_3(t)]$	0.8028	0.8028	0.8064	0.8044	0.7976	0.7969

The number of grid points (allele frequencies *x* and *z*) of the full grid are $|{{{\mathcal {X}}}}|=2N+1=61$ and $|{{{\mathcal {Z}}}}|=2N_e+1=31$ before and after Step 1 of the reproduction cycle. The corresponding numbers for the sparse grid are $|\hat{{{{\mathcal {X}}}}}|=33 = 2\times 13 + 7$ ($K_0(t)=13$ allele frequency intervals of unit length, close to 0 and 1, respectively, and $K_1(t)=7$ allele frequency intervals of length *q=5* for intermediate allele frequencies) and $|\hat{{{{\mathcal {Z}}}}}|=21=2\times 8 + 5$ ($K_{0e}(t)=8$ allele frequency intervals of unit length, close to 0 and 1, respectively, and $K_{1e}(t)=5$ allele frequency intervals of length $q_e=3$ for intermediate allele frequencies). Note that the expected number $F_0(t)$ of lost alleles and the expected number $E[A_{n \mathrm{tot}}(t)]=F_0(t)+E[A_n(t)]$ of lost or present alleles do not converge as $t\rightarrow \infty$, since the lost alleles accumulate over time
